# Epithelial–mesenchymal transition: The history, regulatory mechanism, and cancer therapeutic opportunities

**DOI:** 10.1002/mco2.144

**Published:** 2022-05-18

**Authors:** Zhao Huang, Zhe Zhang, Chengwei Zhou, Lin Liu, Canhua Huang

**Affiliations:** ^1^ State Key Laboratory of Biotherapy and Cancer Center West China Hospital, and West China School of Basic Medical Sciences & Forensic Medicine Sichuan University, and Collaborative Innovation Center for Biotherapy Chengdu 610041 China; ^2^ Department of Thoracic Surgery the Affiliated Hospital of Medical School of Ningbo University Ningbo China

**Keywords:** cancer progression, embryogenesis, EMT, redox signaling, targeted therapy

## Abstract

Epithelial–mesenchymal transition (EMT) is a program wherein epithelial cells lose their junctions and polarity while acquiring mesenchymal properties and invasive ability. Originally defined as an embryogenesis event, EMT has been recognized as a crucial process in tumor progression. During EMT, cell–cell junctions and cell–matrix attachments are disrupted, and the cytoskeleton is remodeled to enhance mobility of cells. This transition of phenotype is largely driven by a group of key transcription factors, typically Snail, Twist, and ZEB, through epigenetic repression of epithelial markers, transcriptional activation of matrix metalloproteinases, and reorganization of cytoskeleton. Mechanistically, EMT is orchestrated by multiple pathways, especially those involved in embryogenesis such as TGFβ, Wnt, Hedgehog, and Hippo, suggesting EMT as an intrinsic link between embryonic development and cancer progression. In addition, redox signaling has also emerged as critical EMT modulator. EMT confers cancer cells with increased metastatic potential and drug resistant capacity, which accounts for tumor recurrence in most clinic cases. Thus, targeting EMT can be a therapeutic option providing a chance of cure for cancer patients. Here, we introduce a brief history of EMT and summarize recent advances in understanding EMT mechanisms, as well as highlighting the therapeutic opportunities by targeting EMT in cancer treatment.

## Graphical abstract

Epithelial–mesenchymal transition contributes to multiple hallmarks of cancer, including metastasis activation, metabolic reprogramming, stemness acquisition, and chronic inflammation, which is regulated by complex signaling network and is highly context dependent. It provides both difficulties and opportunities for cancer treatment via targeting EMT.

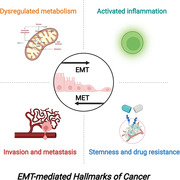

**Abbreviations**
bHLH: basic helix–loop–helix
ceRNA: competing endogenous RNA
circRNA: circular RNA
CSC: cancer stem cell
CTC: circulating tumor cell
DTP: drug‐tolerant persister
E‐cadherin: epithelial cadherin
ECM: extracellular matrix
EGF: epidermal growth factor
EMT: epithelial–mesenchymal transition
EMT‐TF: EMT‐associated transcription factor
EPSC: EMT‐promoting Smad complex
EZH2: enhancer of zeste homolog 2
F‐actin: filamentous actin
FGF: fibroblast growth factor
G‐actin: globular actin
GAP: GTPase‐activating protein
GDI: guanine nucleotide dissociation inhibitor
GEF: guanine nucleotide exchange factor
HDAC: histone deacetylase
HGF: hepatocyte growth factor
IL: interleukin
lncRNA: long noncoding RNA
LSD1: lysine‐specific histone demethylase 1
m6A: N6‐Methyladenosine
M‐cadherin: muscle cadherin
MDR: multidrug resistance
MET: mesenchymal‐epithelial transition
miRNA: microRNA
MMP: matrix metalloproteinase
NAC: N‐acetylcysteine
N‐cadherin: neuronal cadherin
ncRNA: noncoding RNA
OS: overall survival
P‐cadherin: placental cadherin
PRC1/2: polycomb repressive complex 1/2
R‐cadherin: retinal cadherin
ROS: reactive oxygen species
Snail1/2: zinc finger protein SNAI1/2
TAM: tumor‐associated macrophage
TGF: transforming growth factor
Twist1/2: Twist‐related protein 1/2
USP: ubiquitin‐specific protease
ZEB1/2: zinc finger E‐box‐binding homeobox 1/2
ZO: Zona occludens

## 1 INTRODUCTION

Epithelial–mesenchymal transition (EMT) describes a reversible transition process during which epithelial cells reduce their epithelial properties and gain mesenchymal characteristics.^1^ In the reverse process, MET, the transdifferentiated mesenchymal cells can revert back to epithelial state.^1^, ^2^ EMT was initially identified as an embryogenesis event, which is now recognized to be ubiquitous throughout every aspect of life activity, including wound healing, fibrosis, and tumor metastasis.^3^, ^4^, ^5^, ^6^, ^7^, ^8^ During EMT, epithelial cells lose their junction proteins, among them the epithelial cadherin (E‐cadherin) is the most known glue.^9^ Downregulation of E‐cadherin renders cells to be separated with each other, acquiring mesenchymal morphology and invasive capability. Besides, cytoskeleton is also reorganized in this process, which is associated with the formation of pseudopodia and consequent enhanced mobility as well as metastatic capacity.^10^, ^11^, ^12^ Moreover, matrix metalloproteinases (MMPs) secreted during EMT lead to destruction of matrix barrier, making cells ready to move.^13^, ^14^, ^15^, ^16^ Generally, these programs are orchestrated by a group of EMT‐associated transcription factors (EMT‐TFs), including zinc finger protein SNAI1 (Snail1), zinc finger protein SNAI2 (Snail2, also known as Slug), Twist‐related protein 1/2 (Twist1/2), and zinc finger E‐box‐binding homeobox 1/2 (ZEB1/2).^17^, ^18^, ^19^, ^20^, ^21^ These EMT‐TFs induce epigenetic silencing of epithelial marks such as E‐cadherin while activating mesenchymal marks such as N‐cadherin, vimentin, and MMPs, which proceeds EMT program.^22^, ^23^ The upstream signals regulating EMT can be various, many of which are embryogenesis‐related, including TGFβ, Wnt, Hedgehog, and Hippo.^3^, ^24^, ^25^, ^26^, ^27^, ^28^, ^29^, ^30^, ^31^ This fact addresses the intrinsic crosstalk between embryonic development and cancer metastasis.

In terms of cancer biology, EMT is one of the most notable hotspots for its crucial role in the regulation of metastasis, metabolic reprogramming, stemness, inflammation, chemoresistance, and other hallmarks of cancer.^2^, ^20^, ^32^, ^33^, ^34^, ^35^, ^36^, ^37^ In the clinic perspective, EMT is frequently observed in high‐grade tumor cases with poor prognosis.^27^, ^38^, ^39^, ^40^ It is well known that EMT leads to detachment of cancer cells from extracellular matrix (ECM) and subsequent entering into blood, resulting in the generation of circulating tumor cells (CTCs) thus promoting tumor metastasis.^41^ Interestingly, many EMT‐TFs can also activate the transcription of genes related with metabolic reprogramming, stemness, and inflammatory responses, indicating that EMT and many other hallmarks of cancer are inter‐connected.^42^, ^43^, ^44^, ^45^ Furthermore, given that EMT is a highly reversible process, a part of cancer cells is in an intermediate state between epithelial and mesenchymal phenotype (intermediate EMT, also known as hybrid EMT, partial EMT, or incomplete EMT) thus exhibiting plasticity and heterogeneity, which contribute to cancer progression and drug resistance.^2^, ^33^, ^46^, ^47^, ^48^, ^49^ These facts indicate that EMT promotes more aggressive behaviors of tumor but also implicate therapeutic opportunity. For example, loss of epithelial junctions indeed enhances invasive ability, but also makes cancer cells vulnerable to ferroptosis.^50^, ^51^ Therefore, EMT acts as a double‐sword in cancer, which implicates a promising anticancer strategy via selectively inhibiting prometastasis effect or boosting proferroptosis effect of EMT.^52^, ^53^, ^54^, ^55^

Here, we briefly review the history of EMT research and provide several prospects or visions for the future in this field. We summarize recent advances in understanding EMT phenotypes and mechanisms including the loss of junction proteins, reorganization of cytoskeleton, activation of EMT‐TFs, and signal transduction of multiple embryogenesis and redox pathways. We also discuss the impact of EMT on the metabolic reprogramming, stemness acquisition, and inflammatory microenvironment of tumors, and highlight the therapeutic intervention targeting EMT so as to provide new insights into the treatment of cancer.

## 2 A BRIEF RESEARCH HISTORY OF EMT

Elizabeth D Hay is the pioneer who discovered EMT in 1958, though this term was not formally used at that time.^56^, ^57^ She found that during the forelimb regeneration of Amblystoma larvae, the blastema cells can dedifferentiate, proliferate, and then redifferentiate into cartilage, thus contributing to the development of the limb.^56^ Three years later, she used an autoradiography method to label epidermis before amputating limbs, where she observed epidermis cells can migrate over the wound surface which is required for limb regeneration.^58^ These findings show similarity with EMT program and implicate EMT as an important development event during wound healing and tissue biogenesis. In 1966, Elizabeth D Hay and her colleagues reported that the tight junctions are upregulated at the advanced stages of development in chick embryogenesis, making the cells connected with each other thus functioning as a single tissue, rather than separated cell populations.^59^, ^60^ Now we know her observation resembles a MET process in which epithelial marks are reexpressed. The concept of EMT was not so popular until 1968, when Elizabeth D Hay was present at the 18th Hahnemann symposium in Baltimore. At this meeting, she introduced how epithelial cells transformed into mesenchymal cells during the development of neural tube. This speech addressed the importance of the epithelial–mesenchymal interactions during embryonic development and attracted attention of researchers of this field, leading to a rapid evolution of this filed. As a result, a variety of studies were conducted by different research groups to elucidate the roles of epithelial–mesenchymal interactions in organ biogenesis, including the development of heart valve, neural crest, Mullerian duct, intestinal brush border membrane, embryonic lungs, and so on.^61^, ^62^, ^63^, ^64^, ^65^ In 1982, Elizabeth D Hay used the term “epithelial–mesenchymal transformation” to describe the transformation into mesenchymal cells from epithelial cells under the three‐dimensional collagen gel condition, which repressed the apical–basal polarity of epithelial cells and enhanced their mobility.^66^ However, Elizabeth D Hay used another term “epithelial–mesenchymal transition” in 1995, when she summarized several EMT‐promoting genes and the reversed process MET in a review.^67^ After that, “epithelial–mesenchymal transformation” and “epithelial–mesenchymal transition” were both referred to EMT program with no substantial differences. In 2003, the term “epithelial–mesenchymal transition” was confirmed as the official name to describe EMT after the meetings of the EMT International Association, in order to distinguish EMT from malignant transformation used in oncology.^1^

After the extensive research of EMT phenotypes, scientists were curious as to which factors can induce EMT. In 1985, hepatocyte growth factor (HGF) was reported to act as the “scatter factor” to dissolve the junction proteins between epithelial cells, resulting in their morphologic changes and migration.^68^, ^69^ Besides, fibroblast growth factor (FGF) and transforming growth factor (TGF) were both found to induce EMT in rat bladder carcinoma cells in 1990, suggesting the role of EMT in cancer.^70^, ^71^ Another growth factor, epidermal growth factor (EGF), was also demonstrated to promote EMT in rat neonatal hepatocytes by upregulating the expression of vimentin, a crucial mesenchymal mark.^72^ These observations indicated that a variety of growth factors, at least including HGF, FGF, TGF, EGF, are potent inducers of EMT. Among then, TGFβ is probably the most investigated growth factor in EMT research. In 1990, TGFβ was found to dynamically express in mouse endocardial cells according to different embryonic stages, contributing to cardiac development via regulation of EMT.^73^ In the same year, TGFβ was also reported to alter morphology and activate migration in the chicken chorioallantoic membrane, resulting in microvascular angiogenesis.^74^ Given the secretory nature of TGFβ, it is not surprising that the EMT‐promoting function of TGFβ requires its receptor on cell membrane. In 1994, TGFβ was demonstrated to induce EMT in mouse mammary gland NMuMG cells, as evidenced by the decrease of epithelial markers, increase of mesenchymal marks and reorganized cytoskeleton.^75^ Importantly, truncation of Tsk7L type I receptor abolished these EMT phenotypes, indicating that this receptor is indispensable for the EMT‐promoting function of TGFβ.^75^ In addition to the receptor, the activation of TGFβ signaling also involves Smad proteins, suggesting that Smads play certain roles in TGFβ‐induced EMT.^76^ As expected, in 1999, TGFβ was found to promote the nuclear translocation of Smad2/3/4, leading to EMT in NMuMG cells.^77^ These findings indicate that various growth factors, especially TGFβ, are crucial signals activating EMT. Importantly, since 1990s, the roles of EMT in oncology were gradually noted. For instance, TGFβ‐induced EMT was demonstrated to enhance invasiveness of cancer cells, leading to tumor metastasis, which can be abrogated by its neutralizing antibodies.^78^ This observation is in line with the concept that cancer is to some extent a type of developmental disease, given the fact that they share common features such as aberrant EMT program.

Along with the rapid evolution of this field, the mechanisms of EMT were gradually uncovered in 1990s. Indeed, EMT is largely driven by several EMT‐TFs, which initiate complex transcriptional program to regulate EMT mark expression, cytoskeleton organization, pseudopodia formation, MMP secretion, as well as consequent cell migration and invasion. Snail is the first EMT‐TF identified in 1992, when it was found to be involved in the gastrulation during murine development.^79^ And after 2 years, Slug, also known as Snail2, was documented.^80^ Using the antisense oligonucleotides against Slug, EMT events were abolished in early chick embryos, indicating its key roles in vertebrate development.^80^ In 2001, Twist was identified as another EMT‐TF, facilitating the palatogenesis in embryonic rats.^81^ This finding provided an explanation for why mutations of Twist gene result in Saethre‐Chotzen syndrome, a developmental disease in human. In the same year, the zinc finger E‐box‐binding homeobox (ZEB) was demonstrated as an important EMT‐TF.^82^ In this study, ZEB was found to bind with the promoter of E‐cadherin, repressing its transcription. This effect mitigates E‐cadherin‐mediated intercellular adhesion, leading to cell invasion thus being involved in tumor progression.^82^ Actually, the epigenetic silencing of E‐cadherin was found to be a common mechanism shared by all these EMT‐TFs in 2000s, suggesting the central roles of EMT‐TFs in the loss of cell junctions during EMT.^83^, ^84^, ^85^, ^86^ Before long, EMT‐TFs were also reported to be capable of regulating the cytoskeleton organization, pseudopodia formation, and MMP secretion.^87^, ^88^, ^89^, ^90^, ^91^ These evidences indicate EMT‐TFs underlie the molecular basis of EMT program. In addition to their roles in embryonic development, these EMT‐TFs were also investigated in the context of cancer in 2000s. For example, Snail was found to be associated with the progression of poorly differentiated breast carcinoma in 2002.^92^ Two years later, the metastasis‐promoting role of Twist was demonstrated.^9^ Since then, the connection between EMT and cancer has been greatly appreciated.

**FIGURE 1:**
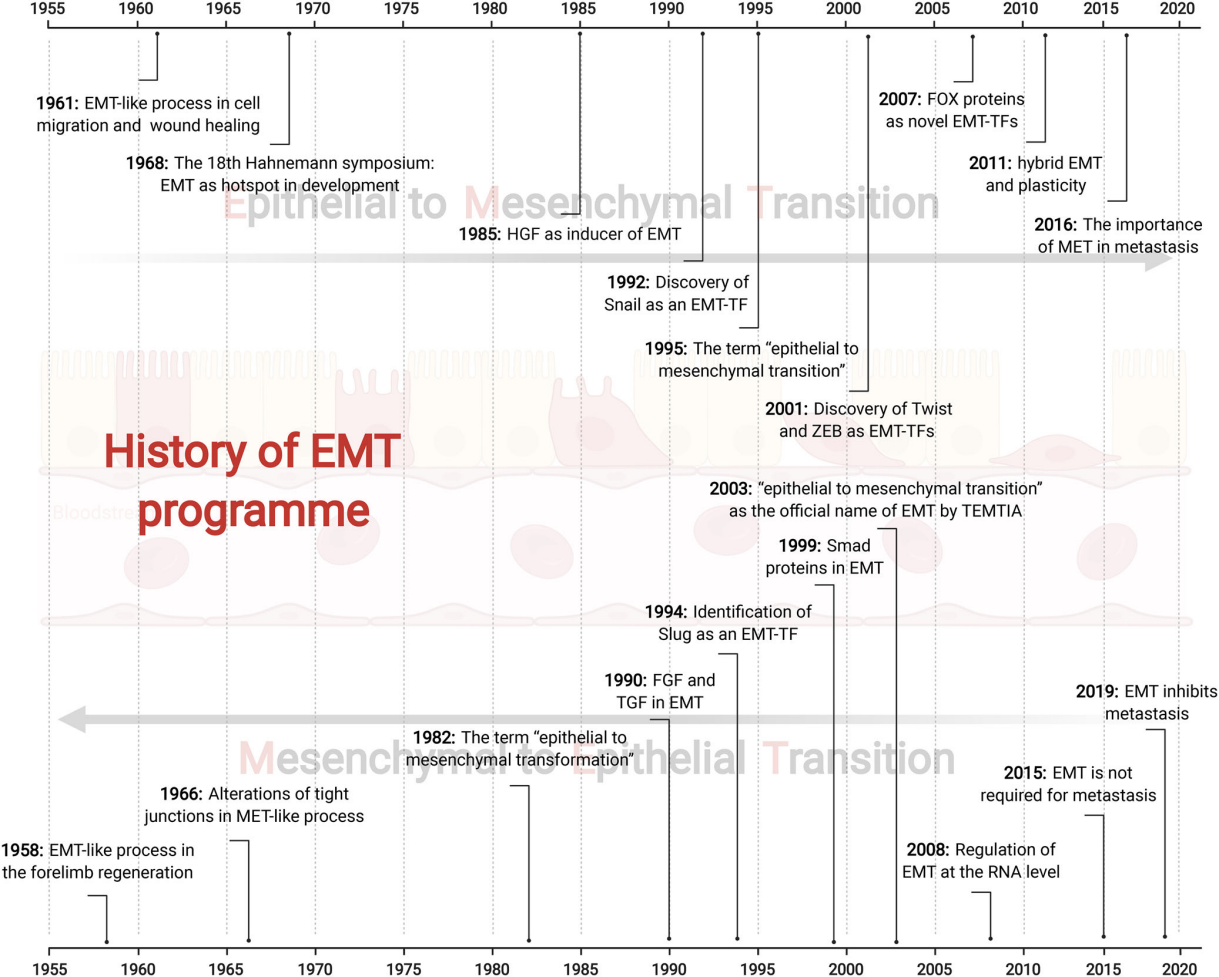
A brief history of EMT. The EMT phenotype was firstly discovered by Elizabeth D Hay, who is the pioneer for this field. From 1950s to1980s, EMT was largely investigated in the context of developmental biology with few mechanistic studies. During 1980s to 2010, the connection between EMT and cancer metastasis was intensively documented, and mechanistic studies revealed the central roles of EMT‐TFs in EMT program. From 2010s to now, new concepts of EMT is increasingly developed, which include novel EMT regulators, the hybrid EMT state, multiple cancer hallmarks induced by EMT, the tumor‐suppressive effects of EMT, and EMT‐based cancer therapeutic strategies

In recent years, the studies focused on EMT and cancer have been more comprehensive, in which several novel mechanisms and concepts were elucidated.^2^, ^20^ First, novel EMT‐TFs were characterized. In 2007, the Forkhead box (FOX) transcription factor FOXC2 was shown to promote breast cancer metastasis through activating EMT.^93^ This finding was followed by the characterization of other FOX family members during EMT, including FOXO3a, FoxF1, FOXA1, FOXA2, and FOXQ1 in the next years.^94^, ^95^, ^96^, ^97^ In addition to FOX proteins, a GATA transcription factor Serpent (Srp) was also found to regulate EMT through repressing E‐cadherin in Drosophila in 2011, and the similar function was also observed in the mammalian orthologs of Srp, GATA4, and GATA6.^98^ Moreover, other novel EMT‐regulating transcription factors, such as PRRX1 and Sox protein family members, were also reported.^99^, ^100^, ^101^, ^102^ Second, EMT was found to be regulated at the RNA level. In 2008, the RNA alternative splicing of p120 was reported to promote invasiveness via regulating EMT.^103^ Three years later, a high‐throughput analysis revealed the alternative splicing signature during EMT, and the key splicing mediators such as RBFOX, MBNL, CELF, hnRNP, and ESRP were also identified in this process.^104^ Moreover, microRNAs (miRNAs) were also demonstrated to regulate EMT either positively or negatively. For instance, miR‐200 and miR‐205 were shown to inhibit EMT through targeting ZEB1 and ZEB2, whereas miR‐9 downregulated the expression of E‐cadherin thereby facilitating EMT.^105^, ^106^ More recently, the circular RNAs (circRNAs) and their alternative splicing factor, Quaking, were presumed to be involved in the regulation of EMT.^107^ Third, EMT was realized as a hybrid process in which epithelial and mesenchymal characteristics coexist, instead of a binary process, in most cases.^108^ In 2011, the intermediate stages of EMT were defined in trophoblast stem cells. These cells expressed both epithelial and mesenchymal marks and acquired higher metastatic potential compared with cells in the beginning epithelial state and ending mesenchymal state.^109^ Following studies revealed that cells in hybrid EMT state were associated with stemness, plasticity, distant colonization, and anoikis resistance, contributing to drug resistance, immune suppression, and tumor recurrence.^110^, ^111^, ^112^, ^113^ Moreover, it has been recently shown that the loss of Fat1 promotes hybrid EMT state of cancer cells via CAMK2–CD44–SRC axis and EZH2–SOX2 axis, which upregulates the mesenchymal properties and maintains the epithelial characteristics, respectively.^114^ This finding provided new insights for understanding mechanisms underlying intermediate EMT state. Fourth, EMT is probably not required for metastasis. In 2015, two independent groups found that inhibition of EMT did not abrogate cancer metastasis, but improved drug sensitivity of tumors.^115^, ^116^ Their observations suggest that EMT is dispensable for metastasis; however, the combinational treatment of chemotherapies with EMT inhibition could be potential strategy for overcoming cancer drug resistance. Fifth, the importance of MET in tumor metastasis was gradually appreciated. In 2016, researchers found that mesenchymal cells arriving at distant organ readily underwent MET program to enter into an epithelial state, which is required for colonization in the final stage of metastasis.^117^ This observation was further supported by the finding in 2019, which described that E‐cadherin facilitates breast cancer metastasis.^118^ Together, these evidences indicate that EMT is a rapidly evolving field (Figure 1).

## 3 REORGANIZATION OF CELL JUNCTIONS AND CYTOSKELETON: KEY CHARACTERISTICS OF EMT

**FIGURE 2:**
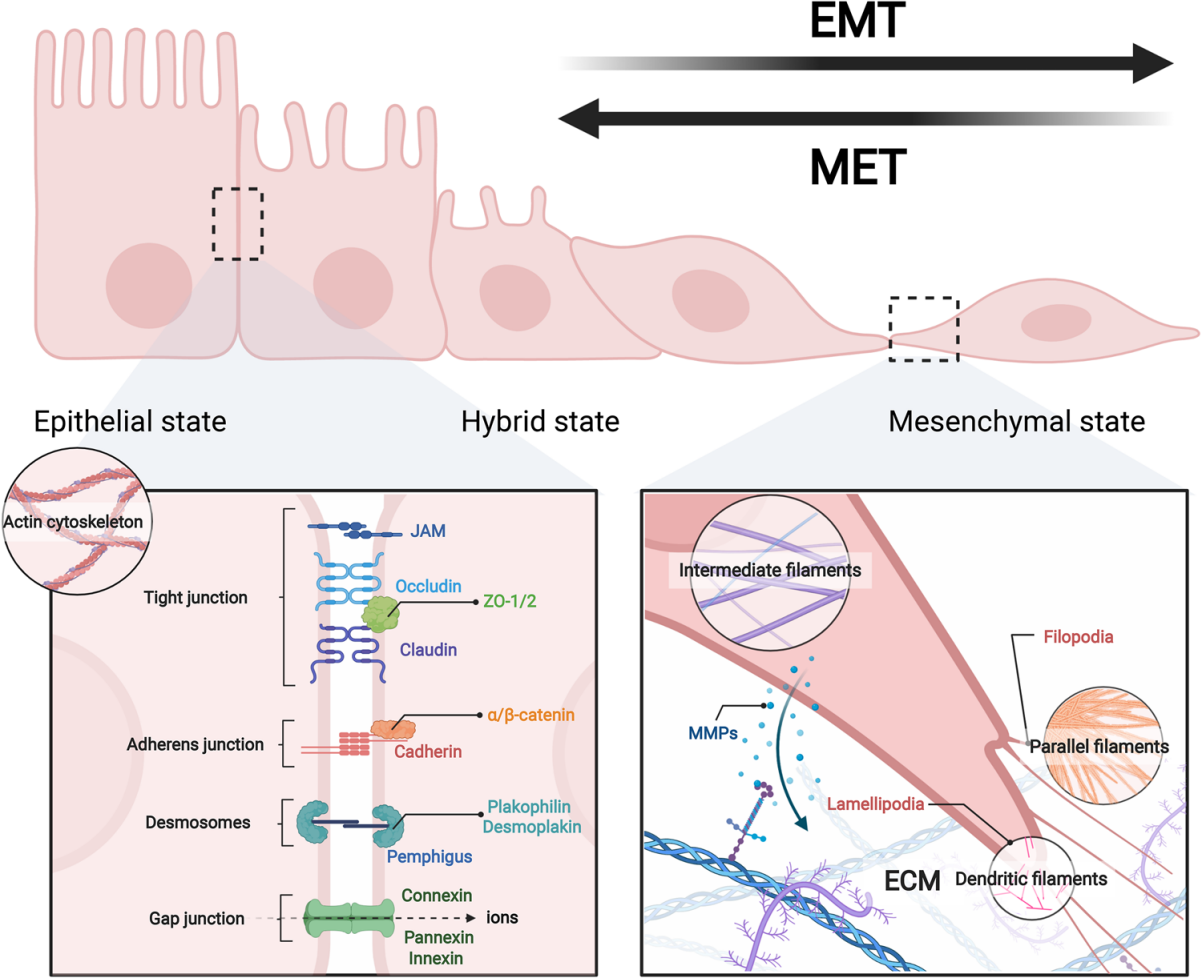
An overview of cellular phenotype changes during EMT. EMT is a highly reversible process with epithelial, hybrid, and mesenchymal states. In epithelial state, cells are hold together to preserve epithelial integrity via several junction structures, namely adherens junctions, tight junctions, desmosomes, and gap junctions, which is composed of several epithelial proteins including E‐cadherin, claudins, occludins, connexins, and many others. Disruption of these junctions leads to the entry of cancer cells into hybrid state and following mesenchymal state, in which cells express mesenchymal marks such as N‐cadherin, Vimentin, and MMPs. In addition, cells reorganize their cytoskeleton networks to support the formation of pseudopodia thereby facilitating metastasis

The integrity of epithelial tissues and the morphology of epithelial cells are maintained by specialized surface proteins and cytoskeleton. Surface proteins form cell–cell junctions and cell–matrix junctions, making epithelial cells as a whole thus restricting individual mobility. Deconstruction of cell junctions, including adherent junctions, tight junctions, desmosomes, and gap junctions, leads to separation of epithelial cells with each other and disassociation with basement membrane, as well as the loss of cell contact inhibition.^119^ These events result in loss of apical–basal polarity, thus facilitating metastasis. In addition to their well‐known metastasis‐suppressive functions, several junction proteins can also promote tumor dissemination, such as claudin‐11.^120^ This observation suggest that junction proteins play dual roles in tumor metastasis. Moreover, crosstalk exists among these different types of cell junctions. For instance, desmoplakin, one of the components of desmosomes, is able to maintain gap junctions through regulating Ras/MAPK signaling.^121^ As transmembrane proteins, junction proteins are commonly associated with Rho GTPases through their cytoplasmic domains, thereby regulating cytoskeleton organization.^122^ Cytoskeleton controls the morphology and mobility of cells, reorganization of which promotes morphological change of epithelial cells into a spindle‐like mesenchymal shape. During this process, pseudopodia is elongated to enable directional motility and consequent cell movement. In addition to these physical effects, emerging evidence showed that junction proteins and cytoskeleton can also function as signaling molecules to regulate signal transduction, thereby affecting invasiveness of cells.^123^, ^124^ Actually, cell junctions mediate cell–cell communications mediating nonautonomous behaviors of cells, whereas cytoskeleton might serve as scaffold to facilitate biochemical reactions via providing reaction places. Therefore, reorganization of cell junctions and cytoskeleton are key characteristics during EMT (Figure 2).

### 3.1 Altered junctional components

There are four common junction types that comprise the epithelial connection, namely adherent junctions, tight junctions, desmosomes, and gap junctions. One of the most known adherent junctions is the transmembrane protein E‐cadherin (epithelial cadherin, encoded by CDH1 gene), whose cytoplasmic region binds with β‐catenin and p120 catenin, thereby being associated with cytoskeleton. The extracellular fragment of E‐cadherin provides intercellular adhesion between opposing epithelial cells in a calcium‐dependent manner.^125^ In normal condition, E‐cadherin is one of the most important epithelial marks. Given the fact that most tumors originate from epithelial tissues, deregulation of E‐cadherin has been regarded as a hallmark of tumorigenesis. Indeed, in the context of neoplasm, E‐cadherin has long been proved as a tumor‐suppressor gene inhibiting cancer initiation and progression. Loss of E‐cadherin, often caused by epigenetic silencing or genetic mutations, is a common event in a wide range of tumors, including breast cancer, gastric cancer, colorectal cancer, liver cancer, lung cancer, and so on.^30^, ^126^, ^127^, ^128^, ^129^ Interestingly, E‐cadherin has been also shown considerable expression level in tumor metastases.^130^ Moreover, a recent study indicated that E‐cadherin is required for tumor metastasis by buffering oxidative stress in breast cancer.^118^ One possible explanation is that E‐cadherin has to be reexpressed in MET process, which facilitates the distal colonization in the late stage of metastasis. Other adherent junctions, such as N‐cadherin (neuronal cadherin, CDH2), P‐cadherin (placental cadherin, CDH3), R‐cadherin (retinal cadherin, CDH4), and M‐cadherin (muscle cadherin, CDH15), share similar structure but their functions are distinct.^131^ N‐cadherin is a mesenchymal mark promoting tumor metastasis, which is opposite to E‐cadherin.^132^ R‐cadherin is an epithelial mark resembling E‐cadherin, loss of which has been shown to facilitate EMT and tumor progression.^133^ Interestingly, the role of P‐cadherin can be either tumor promoting or tumor suppressive, depending on particular context.^134^ For instance, the high expression of P‐cadherin was correlated with the progression of lung cancer and ovarian cancer,^135^, ^136^ whereas other reports showed that P‐cadherin preserves epithelial barrier to inhibit the metastasis of melanoma.^137^, ^138^ These dual functions of P‐cadherin in regulating cancer metastasis might be attributed to the distinct characteristics of different cancer types. To be specific, in response to gonadotropin‐releasing hormone (GnRH), P‐cadherin induces the activation of IGF‐1R in a ligand‐independent manner, which phosphorylates p120 catenin, thereby promoting metastasis in ovarian cancer.^136^ However, other tumor types such as melanoma might not be relevant to GnRH, IGF‐1R, or p120 catenin. In other words, GnRH, IGF‐1R, or p120 catenin might be with very low basal level or loss of function in other cancers, leading to the disruption of this pro‐metastatic signaling, therefore P‐cadherin failed to activate metastasis in such cancers. In this context, P‐cadherin may serve as intercellular glue to prevent metastasis. Certainly, this postulation needs to be validated by substantial evidence.

Apart from adherent junctions, the epithelial integrity is also maintained by tight junctions, which consist of claudins, zonula occludens, and others. These tight junction proteins contain several family members. For instance, 27 members are characterized in claudin family, whereas only three members are found in zonula occluden family.^139^ Similar to adherent junctions, tight junctions also play crucial roles, either positive or negative, in the EMT phenotype and cancer progression. Claudin‐1 was shown to promote EMT and invasion of colorectal cancer through upregulating ZEB‐1, while inhibiting tumor metastasis in gastric cancer via mediating the tumor‐suppressive function of RUNX3.^140^, ^141^ Besides, it has been reported that claudin‐2 promotes tumor metastasis in breast, lung, and colorectal cancer, but is negatively associated with the high‐grade pancreatic cancer.^142^, ^143^, ^144^, ^145^, ^146^ Therefore, the specific roles of claudin proteins in EMT status and tumor progression are largely context dependent. Another tight junction proteins, Zona occludens (ZO), are also key components of epithelial tissue. There are three members found in ZO family, namely ZO‐1, ZO‐2, and ZO‐3. Among them, ZO‐1 is the most investigated one in the neoplastic context. For example, loss of ZO‐1 was reported to promote metastasis of breast cancer, colorectal cancer, liver cancer, pancreatic cancer, and so on.^147^, ^148^, ^149^, ^150^ These observations indicate that ZO‐1 mainly serves as a tumor suppressor, in contrast to the diverse functions of abovementioned claudin proteins. However, a recent study provided exceptional evidence describing that ZO‐1 can activate Rac‐1‐mediated cytoskeletal organization, thereby promoting metastasis in colorectal cancer.^151^ More intriguingly, different tight junction proteins can be associated with each other, thereby forming complex junction architecture. For instance, the cytoplasmic region of claudins is able to bind with the PDZ domain of zonula occludens.^152^ Though physical association is observed, the functional link between claudins and ZO proteins remains elusive.

Desmosomes represent another form of cell junctions, which consist of desmosomal cadherins (including desmogleins and desmocollins), armadillo proteins (including plakoglobins and plakophilins), and desmoplakin. These proteins form complex structure anchoring the intermediate filaments to the plasma membrane between neighboring cells, thus maintaining epithelial integrity.^153^ As tumor suppressors, downregulation of desmosomal components plays vital roles in EMT program and consequent cancer progression.^154^ For example, impaired desmosomes were shown to promote EMT and consequent tumor progression in invasive breast cancer.^155^ Besides, loss of desmosomes was observed in invasive pancreatic neuroendocrine tumors (PNET), and genetic deletion of desmoplakin enhanced tumor metastasis in the PNET mouse model.^156^ Interestingly, the proper functions of desmosomes require particular posttranslational modifications on several components. Palmitoylation of plakophilin was shown to play critical roles in the assembly of desmosomes, and dephosphorylation of plakophilin‐1 is able to promote epidermal carcinogenesis.^157^, ^158^ In fact, the tumor‐suppressive roles of desmosomes were also reported in several other tumor types, including liver cancer, breast cancer, and lung cancer.^159^, ^160^ Even so, the oncogenic roles of desmosomal components have been also reported. For instance, desmoglein‐3 is overexpressed in human head and neck cancer and associated with advanced tumor stage, and inhibition of desmoglein‐3 is sufficient to mitigate tumor progression both in vitro and in vivo.^161^ In addition to desmoglein‐3, the tumor‐promoting functions of several other desmosomal components were recently summarized elsewhere.^162^ This evidence suggest that desmosomes might not be simply regarded as tumor suppressive molecules, but rather multifunctional structure during tumor development.

Gap junctions are ion channels formed by connexin, pannexin, and innexin proteins, which provide both adherence and direct intercellular communication between neighboring cells.^163^ Among them, connexins are the most investigated channels. Similar to other junction proteins, connexins play dual roles in tumorigenesis. It has been reported that connexins are overexpressed in tumors, such as connexin‐26 in pancreatic cancer and colorectal cancer.^164^, ^165^ However, it has been also shown that connexins can serve as tumor suppressors, including connexin‐43 and connexin‐45 in colorectal cancer.^166^, ^167^ Moreover, high expression of connexins can predict either better or poor prognosis in cancer patients. For instance, overexpression of connexin‐43 prolongs the survival of patients with prostate cancer, breast cancer, and colorectal cancer,^166^, ^168^, ^169^ whereas accelerating death of patients with bladder cancer, esophageal squamous cell carcinoma, and oral squamous cell carcinoma.^170^, ^171^, ^172^ Not surprisingly, gap junction‐regulated EMT and tumor progression are largely dependent on their ion channel function, which provide communication between nearby cells, in addition to their adherent effect. It has been reported that gap junctions among U2OS cells failed to inhibit EMT, but gap junctions between U2OS cells and osteoblasts did, suggesting that gap junctions inhibit EMT through U2OS‐osteoblast communication rather than merely intercellular glue.^173^ Moreover, gap junction was shown to amplify potassium currents, thereby establishing electrochemical communication between neuron and glioma. This effect significantly promotes glioma progression, which can be abrogated by gap junction antagonists.^174^ Thus, targeting gap junctions can be a potential therapeutic strategy for cancer treatment.

### 3.2 Cytoskeleton reorganization

As mentioned above, cell–cell junctions are linked with actin cytoskeleton to form epithelial architecture, suggesting the vital roles of cytoskeleton organization during EMT program. Indeed, junction proteins are associated with Rho GTPases, which are dominantly responsible for the organization of actin cytoskeleton.^10^, ^175^ The activity of Rho GTPases is finely tuned by three classes of regulators, namely guanine nucleotide exchange factors (GEFs), GTPase‐activating proteins (GAPs), and guanine nucleotide dissociation inhibitors (GDIs).^176^ In response to adherent or growth factor signals, Rho GTPases are activated by the exchange of GDP with GTP, and this process is catalyzed by GEFs. In contrast, Rho GTPases can be inactivated by GAP‐mediated hydrolyzation of GTP into GDP. GDIs controls the subcellular localization of Rho GTPases by forming protein complex. To date, 20 Rho GTPase family members are identified, among which RhoA, Rac1, and Cdc42 are the most documented, especially in neoplastic context.^177^, ^178^ For example, RhoA has been found to be overexpressed in colorectal cancer, breast cancer, lung cancer, ovarian cancer, gastric cancer, and so on.^179^, ^180^, ^181^ The upregulation of Rac1 can be observed in prostate cancer, gastric cancer, breast cancer and leukemia.^179^, ^181^, ^182^, ^183^ Cdc42 was reported to be overexpressed in colorectal cancer, breast cancer, lung cancer, and melanoma.^179^, ^184^, ^185^, ^186^ Interestingly, RhoA gene was shown to be rarely amplified but frequently deleted in a wide range of tumors according to The Cancer Genome Atlas dataset, suggesting its tumor suppressive role which is somewhat contradictory to most literatures.^177^ Rho GTPases regulate actin polymerization through complex mechanisms, thereby organizing cytoskeleton. In this process, globular actin (G‐actin) is polymerized to form filamentous actin (F‐actin), whereas Arp2/3 complex is one of the key molecular machines enabling this.^187^ For example, Rac1 can bind with the nucleation promoting factor WAVE, which activates Arp2/3 to generate branched actin networks.^187^, ^188^ Similarly, Cdc42 interacts with N‐WASP, leading to the activation of Arp2/3 thus playing critical role in actin polymerization.^189^, ^190^ Aberrant formation of F‐actin in cancer cells is closely correlated with EMT and metastasis of a variety of tumors, including hepatocellular carcinoma, glioblastoma, pancreatic cancer, bladder cancer, breast cancer, and so on.^30^, ^191^, ^192^, ^193^, ^194^, ^195^, ^196^, ^197^ Besides, the antagonists for actin polymerization, latrunculin A/B, have been shown anticancer effects through disrupting the formation of F‐actin.^30^, ^198^, ^199^, ^200^ This evidence indicates that the organization of actin cytoskeleton network profoundly affects EMT and tumor progression.

Cytoskeleton reorganization frequently leads to the formation of membrane protrusions, namely lamellipodia, filopodia, invadopodias, and podosomes, thus contributing to the migration and invasion of tumor cells. Lamellipodia and filopodia are defined as sheet‐like and spike‐like extensions, respectively. Both protrusions are present on the leading edge of migrating cells, which determine the movement direction of cells.^201^ Invadopodia appears on the ventral surface of membrane, and often involved in the degradation of ECM via MMPs.^202^ Podosomes are similar to, but less effective in ECM degradation than invadopodia.^203^, ^204^ These membrane protrusions can be visualized from microscopy, thus being useful marks for EMT. Compelling evidence suggests the crucial roles of membrane protrusions in EMT and tumor progression. For instance, formation of filopodia encouraged by EMT program facilitates both initiation and metastatic colonization in breast cancer.^205^ Besides, invadopodia is correlated with the EMT program and consequent metastasis in a variety of tumors, including hepatocellular carcinoma, breast cancer, bladder cancer, and so on, suggesting invadopodia as a potential prognostic marker for tumor metastasis.^206^, ^207^, ^208^, ^209^, ^210^ Given their critical roles in tumor metastasis, inhibitors targeting these protrusions can be of therapeutic value. For example, lidocaine has been shown to reduce metastatic dissemination of breast cancer by inhibiting the formation of invadopodia.^211^ However, the formation and turnover of these membrane protrusions are highly dynamic and the duration ranges from minutes to hours, targeting these structures might be with off‐target effects thus waiting for further investigation.

## 4 ACTIVATION OF KEY TRANSCRIPTION FACTORS

**FIGURE 3:**
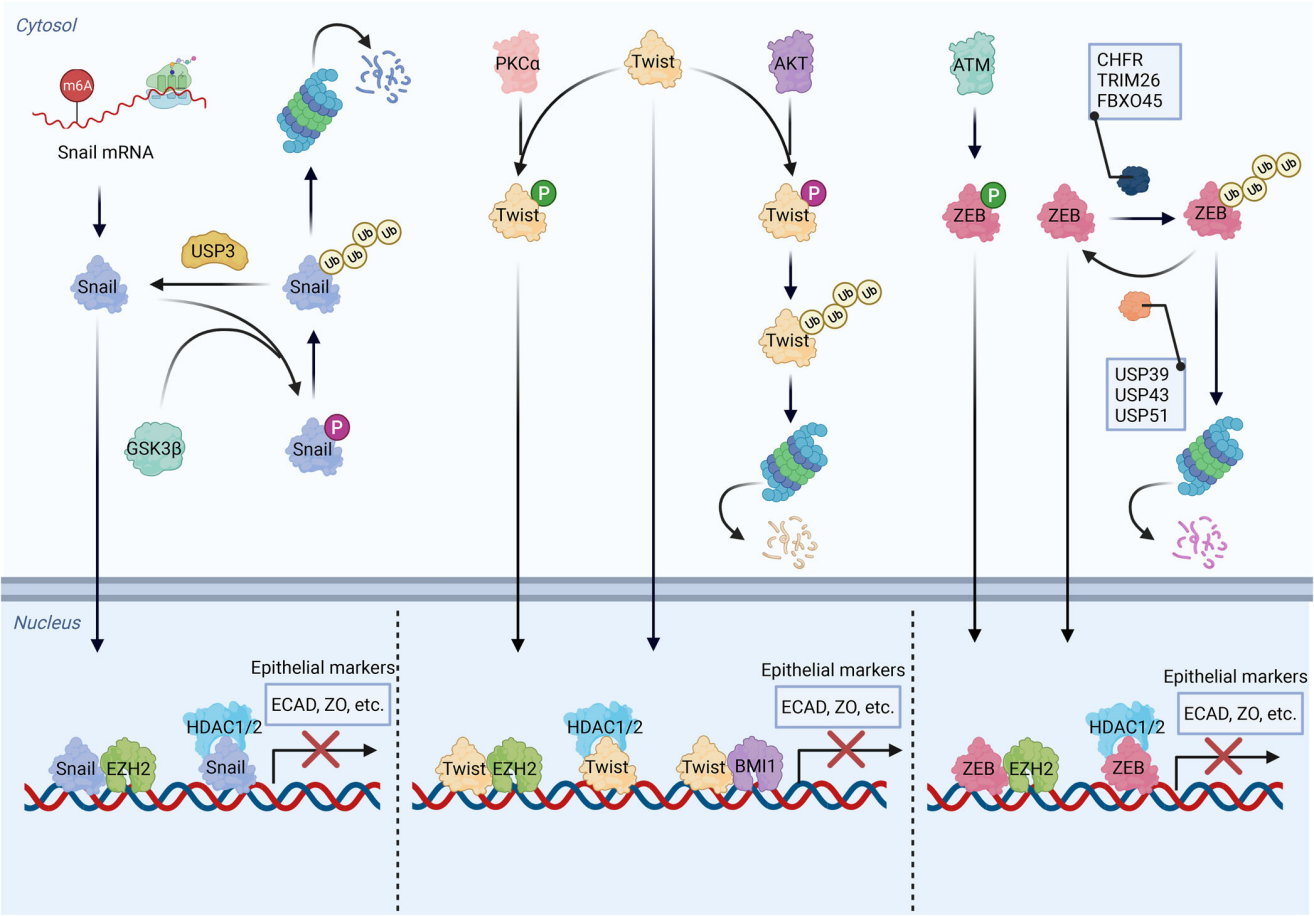
EMT‐TFs are key drivers of EMT program. The EMT program is controlled by several EMT‐TFs, including Snail, Twist, and ZEB. These EMT‐TFs are regulated at transcriptional and posttranslational levels, such as protein phosphorylation, ubiquitination, and RNA m6A modification. The m6A modification on EMT‐TF mRNAs can be recognized by different m6A readers, which facilitate the translation or promote RNA decay. The protein phosphorylation is coordinated by kinases and phosphatases, whereas the ubiquitination can be balanced via E3 ligases and deubiquitinases. When translocated into nucleus, EMT‐TFs bind with different epigenetic modifiers such as EZH2, HDAC1/2, BMI1 to form transcriptional complexes, thereby regulating EMT program

Cells that undergo EMT program are characterized by a global change in gene expression, resulting in loss of epithelial marks and gain of mesenchymal properties. This process is largely regulated at the transcription level by several EMT‐TFs, including Snail (encoded by SNAI1), Slug (SNAI2), Twist‐related proteins, zinc‐finger E‐box‐binding (ZEB) proteins, and others (Figure 3). During EMT, these EMT‐TFs are activated at the transcriptional level (e.g., transcribed by other TFs) and posttranslational level (e.g., phosphorylation and ubiquitination), which have been observed in various cancer thus can be potential therapeutic targets. One of the most known target genes of EMT‐TFs is CDH1, which encodes the important epithelial mark E‐cadherin. Nearly all EMT‐TFs can repress the transcription of E‐cadherin through similar mechanisms, suggesting the functional redundancy between them.^212^ However, these EMT‐TFs differ from each other in many aspects, such as individual structure, size, tissue specificity, binding partner, and target preference. Therefore, the specific, nonredundant functions of EMT‐TFs are gradually appreciated.^213^ Indeed, EMT‐TFs are spatiotemporally regulated thus contributing to distinct expression patterns in different cancer types, which is correlated with specific characteristics of different tumors such as drug sensitivities.^214^ The idea of nonredundant functions of EMT‐TFs can be supported by plenty of evidence. First, an EMT‐TF can be either oncogenic or tumor suppressive. For instance, the tumor‐promoting role of ZEB1 has been widely reported.^17^, ^215^, ^216^ However, ZEB1 can also function as a tumor suppressor to inhibit the progression of acute myeloid leukemia (AML).^217^ Moreover, in KRAS‐mutated lung cancer, ZEB1 was shown to inhibit tumor progression via repressing ERBB3.^218^ Given the fact that EMT‐TFs are oncogenic in most cases, those tumor suppressive effects directly demonstrate their nonredundant functions. Second, different EMT‐TFs can exhibit diverse expression pattern in the same cancer type. For example, Twist1 and ZEB1 were reported to be respectively overexpressed and downregulated in lung cancer.^218^ This finding indicates that Twist1 and ZEB1 regulate lung cancer through contrary ways. Third, different members in the same EMT‐TF protein family can play opposite roles. For instance, ZEB1 was shown to promote the progression of melanoma, whereas ZEB2 functions as a tumor suppressor to inhibit this process.^4^, ^219^ Fourth, different members in the same EMT‐TF protein family can be selectively activated by the same upstream signal. In response to proangiogenic factor SDF1α, endothelial Slug, but not Snail, is activated thus contributing to pathological angiogenesis and tumor growth.^220^ Moreover, one EMT‐TF can be regulated by another EMT‐TF. It has been shown that Snail can transiently repress the transcription of Twist1 in response to TGFβ stimuli, which is followed by Snail degradation, Twist1 reexpression, and consequent EMT as well as tumor metastasis in breast cancer.^221^ This evidence suggest that different EMT‐TFs can form complex regulatory network to coordinate the EMT program, and a single EMT‐TF may not be sufficient to dictate cancer metastasis in some circumstances.

### 4.1 Snail

Snail proteins include Snail1 (Snail) and Snail2 (Slug), which promote EMT and metastasis in a variety of cancer through the epigenetic regulation. Briefly, Snail harbors four zinc‐finger motifs in its carboxy‐terminal region, which bind with the E‐box DNA sequence of target genes.^20^ This event facilitates the recruitment of the polycomb repressive complex 2 (PRC2), which contains the methyltransferase enhancer of zeste homolog 2 (EZH2). The recruitment of PRC2 leads to DNA methylation as well as repressive histone modifications such as H3K9me2, H3K9me3, and H3K27me3 on the promoter of target genes, resulting in epigenetic silencing.^222^ A variety of junction proteins are repressed through this mechanism, including E‐cadherin, occludin, ZO‐1, claudin‐3, claudin‐5, claudin‐7, and so on.^83^, ^223^, ^224^, ^225^, ^226^ Interestingly, PRC2 also induces active marks, such as H3K4me3.^227^ This mark might be associated with the MET program, or the increase of mesenchymal proteins including N‐cadherin and MMP‐9.^228^, ^229^ In addition to PRC2, Snail also recruits other epigenetic modifiers such as histone deacetylases (HDACs) and lysine‐specific histone demethylase 1 (LSD1), thereby regulating EMT program.^230^, ^231^, ^232^ For example, Snail has been found to form protein complex with HDAC1 and HDAC2 at the promoter of E‐cadherin, leading to the deacetylation and consequent repression of E‐cadherin, which results in the EMT and migration of breast cancer, pancreatic cancer, and nasopharyngeal cancer.^233^, ^234^, ^235^, ^236^ To date, the mechanisms underlying the preference of Snail to different epigenetic modifiers remain unclear.

The expression and function of Snail can be regulated at transcriptional level, posttranscriptional level, and posttranslational level. The transcription of Snail involves other transcription factors, such as NF‐κB, YY1, and even Snail itself.^237^, ^238^, ^239^ Particularly, several transcription factors capable of regulating Snail are the components of embryogenesis‐related pathways, including YAP (component of Hippo pathway), Gli1 (Hedgehog), Smad (TGFβ), and many others.^240^, ^241^, ^242^ This connection between EMT and embryonic development signaling will be discussed in the following section. The regulation of Snail at the posttranscriptional level is evidenced by N6‐Methyladenosine (m6A) modification on its mRNA.^243^ Briefly, the methyltransferase‐like 3 (METTL3) induces m6A modification in Snail CDS, which can be recognized by YTH domain‐containing family protein 1 (YTHDF1).^243^ This event facilitates polysome‐mediated translation of Snail, leading to EMT and metastasis of tumor cells.^243^ Besides, it has been reported that UDP‐glucose enhances the stability of Snail mRNA, resulting in the overexpression of Snail and the metastasis of lung cancer.^244^ In terms of its posttranslational regulation, the most known mechanisms are phosphorylation and ubiquitination. For instance, GSK‐3β‐mediated phosphorylation of Snail at its first motif promotes its ubiquitination and subsequent degradation, whereas phosphorylation at its second motif leads to its cytoplasmic retention.^245^ In contrast, phosphorylation of Snail at Ser249 by the PAR‐atypical protein kinase C (aPKC) inhibits the ubiquitination of Snail, thus promoting tumor metastasis.^246^ Besides, the ubiquitin‐specific protease 3 (USP3) has been shown to stabilize Snail via deubiquitination.^247^ Interestingly, aforementioned transcription factor, NF‐κB, also regulates the ubiquitination of Snail.^248^ Another member of Snail protein family, Slug, is also regulated by ubiquitination. It has been shown that several deubiquitinases, including USP5, USP10, USP20, counteract the ubiquitination of Slug therefore enhancing its protein stability.^249^, ^250^, ^251^ Moreover, recent study showed that Slug can be SUMOylated through the interaction with Ubc9 and SUMO‐1, which enhances the transcriptional repression activity of Slug and promotes the progression of lung cancer.^252^

### 4.2 Twist

Twist protein family consists of two members Twist1 and Twist2, which belong to the basic helix–loop–helix (bHLH) transcription factors.^253^ Based on their structural similarities and functional redundancy, we mainly discuss Twist1 here although the differences between them have been reviewed elsewhere.^254^ As one of important EMT‐TFs, Twist1 has long been associated with cancer progression. For instance, knockout of Twist1 was shown to abrogate tumor metastasis in breast cancer.^255^ Similar to Snail, Twist1 represses the epithelial marks such as E‐cadherin, α‐catenin, γ‐catenin and upregulates mesenchymal marks including vimentin, fibronectin, N‐cadherin, leading to EMT and cancer progression.^9^, ^256^ Aforementioned epigenetic repressing complex, PRC2, can be also recruited by Twist1 to induce the H3K27me3 modification at Ink4A/Arf locus, thus preventing the senescence of mesenchymal stem cells.^257^ Besides, Twist1‐mediated EMT program involves other epigenetic modifiers, such as the methyltransferase SET8, the PRC1 component BMI1, and the NuRD transcriptional repressive complex. For example, during breast cancer metastasis, Twist1 interacts with SET8 thus inducing a H4K20 monomethylation mark at the promoter of E‐cadherin and N‐cadherin, which downregulates and upregulates the transcription, respectively.^258^ Besides, Twist recruits BMI1 to repress the transcription of E‐cadherin, and this effect is correlated with the poor prognosis of head and neck cancers.^259^ Furthermore, it has been reported that Twist1 can recruit NuRD complex, which contains HDAC1/2, to decrease the acetylation of H3K9 at Foxa1 promoter, thereby repressing the expression of Foxa1.^260^ This event contributes to the Twist1‐induced metastasis of breast cancer, but less responsible for Twist1‐induced EMT phenotype of breast cancer cells, which is interesting.^260^

Compelling evidence has suggested that Twist1 can be regulated at transcriptional level and posttranslational level. In neuroblastoma, both N‐Myc and c‐Myc proteins physically associate with the promoter of Twist1, thus activating its transcription.^261^ Other transcription factors, such as Sox12 and Sox13, can also transcribe Twist1 thus leading to EMT and metastasis of HCC cells.^262^, ^263^ In contrast, the bHLH transcription factors BHLHE40 and BHLHE41 inhibit the transcription of Twist1.^264^ The posttranslational modifications on Twist1 include phosphorylation and ubiquitination. For example, PTEN with K27‐linked polyubiquitination (PTEN^K27‐polyUB^) can dephosphorylate Twist1 at Ser123, resulting in the nuclear translocation of Twist1 and subsequent EMT phenotype.^265^ In terms of the ubiquitination, the E3 ligase Pirh2 is able to prime Twist1 for degradation.^266^ Interestingly, the RING‐finger E3 ligase RNF8 promotes the K63‐linked ubiquitination of Twist1 at K38, which enhances, but not decreases, the protein stability of Twist1, leading to its nuclear localization thereby playing critical roles in cancer drug resistance.^267^ It is worth noting that there is causal relationship between the phosphorylation and ubiquitination of Twist1, as evidenced by the facts that protein kinase Cα (PKCα)‐mediated phosphorylation of Twist1 at Ser144 diminishes the ubiquitination of Twist1, whereas AKT1‐mediated phosphorylation of Twist1 at Ser42, Tyr121, and Ser123 enhances its ubiquitination.^268^, ^269^ Sometimes, these posttranslational modifications regulate Twist1 activity through affecting protein–protein interaction. For instance, LYN‐mediated phosphorylation of Twist1 leads to the dissociation between Twist1 and its cytoplasmic anchor G3BP2, resulting in the nuclear translocation of Twist1.^270^, ^271^ This event underlies the nature of Twsit1 as a mechanomediator in response to mechanical cues such as matrix stiffness.^270^, ^271^ Besides, the association between Twist1 and another binding partner TGIF1 is able to inhibit Twist1, whereas this inhibitory effect can be abolished by the phosphorylation of TGIF1 in pancreatic ductal adenocarcinoma.^272^ Interestingly, Twist1 can bind with itself to form a homodimer, regulating fibroblast activation, cell migration, and embryonic development.^273^, ^274^

### 4.3 ZEB

ZEB is potent EMT inducer, which represses epithelial marks including E‐cadherin, ZO‐1, claudin‐1, desmoplakin, and is implicated in various type of cancer such as gastric cancer, leukemia, squamous cell carcinoma, liver cancer, colorectal cancer, and so on.^82^, ^140^, ^150^, ^159^, ^275^, ^276^, ^277^, ^278^ There are two members in vertebrate ZEB protein family, namely ZEB1 and ZEB2, which share structural and functional similarities but are also distinguished by considerable differences. For example, a switch from ZEB2 to ZEB1 promotes the progression of melanoma, suggesting these two ZEB proteins function in an opposite manner under this condition.^4^ These diverse effects of ZEB1 and ZEB2 can be explained by different epigenetic modifiers recruited by them. ZEB1 binds p300 to activate transcription, whereas ZEB2 interacts with C‐terminal‐binding protein (CTBP) to silence the target genes.^279^ In addition, ZEB1 has been shown to recruit the SWI/SNF chromatin‐remodeling protein BRG1, thereby repressing E‐cadherin independently of CTBP.^280^ Moreover, ZEB1 can also recruit NuRD complex, which contains HDAC1/2, to promote the EMT and tumor progression in pancreatic cancer and lung cancer.^281^, ^282^ Similarly, the NuRD complex can also recruited by ZEB2, thus regulating the metastasis of breast cancer and the differentiation of neural cells.^283^, ^284^

A variety of transcription factors have been found to activate ZEB1, such as Snail1, Twist, ETS1, and myocyte enhancer factor 2A (MEF2A).^285^, ^286^ In addition to this transcriptional regulation, ZEB1 is also regulated at the protein level, such as phosphorylation. For instance, ERK phosphorylates ZEB1 at Thr867, which inhibits its nuclear translocation, DNA binding, and function of transcriptional repression.^287^ This ERK–ZEB1 axis is involved in the progression of various tumors, including lung cancer, breast cancer, liver cancer, prostate cancer, ovarian cancer, and glioblastoma.^288^, ^289^, ^290^, ^291^, ^292^, ^293^ Interestingly, ERK also activates ZEB1 through upregulating aforementioned transcription factor ETS1, suggesting that ERK can regulate ZEB1 in either direct or indirect manner.^294^ Moreover, ZEB1 has been also reported to be phosphorylated by ATM at S585, which enhances its protein stability in breast cancer cells.^295^ Similar to other EMT‐TFs, ZEB1 can also be regulated by ubiquitination. For example, the E3 ligase tripartite motif‐containing 26 (TRIM26) downregulates ZEB1 through ubiquitination‐mediated protein degradation, whereas USP39 acts as a deubiquitinase to stabilize ZEB1.^296^ Thus, TRIM26 and USP39 function in an antagonistic manner to coordinate the fate of liver cancer.^296^ Besides, the ubiquitination of ZEB1 involves additional E3 ubiquitin ligases such as checkpoint with Forkhead and ring finger domains (CHFR), F‐box only protein 45 (FBXO45), and deubiquitinases including USP51 and USP43.^297^, ^298^, ^299^, ^300^ As to ZEB2, the regulation of protein stability is associated with E3 ubiquitin ligases FBXW7 and TRIM14.^301^, ^302^ These observations indicate that the regulation of ZEB proteins is rather complex in tumor progression.

### 4.4 Novel EMT‐regulating transcription factors

As a rapidly evolving field, more transcription factors have been identified as novel regulators of EMT, such as PRRX1 and Sox. PRRX1 cooperates with Twist1 to induce EMT during embryogenesis and tumor invasion, but overexpression of PRRX1 is associated with a favorable prognosis in breast cancer patients.^99^ Mechanistically, loss of PRRX1 induces MET, which is required for metastatic colonization of breast cancer cells.^99^ Moreover, PRRX1 loss‐mediated MET also confers cancer cells with stemness, leading to drug resistance thus further explaining why PRRX1 overexpression predicts good prognosis.^99^, ^303^ However, opposite finding showed that upregulated PRRX1 induces EMT, cancer stemness, metastasis, and poor prognosis in colorectal cancer, suggesting the role of PRRX1 is highly context‐dependent in different tumor types.^100^ This contradiction might be attributed to the diverse functions of PRRX1 isoforms, namely PRRX1b that activates EMT, whereas PRRX1a that activates MET, and this isoform switching underlies tumor invasion and metastatic colonization in pancreatic cancer.^304^ Sox protein family consists of over 20 members in vertebrates, many of which are involved in tumor initiation and progression.^305^ Numerous studies indicated that Sox proteins regulate EMT via classical EMT‐TFs. For instance, Sox13 transcriptionally activates Twist, thereby promoting EMT in liver cancer.^263^ Interestingly, several Sox proteins have been shown to modulate EMT through either direct or indirect way. Sox4 binds with the promoter of Slug to activate its transcription, leading to EMT in uterine carcinosarcoma.^306^ Alternatively, Sox4 can also directly transcribe N‐cadherin, thus inducing EMT independent of classical EMT‐TFs.^101^ Another Sox protein, Sox9, can directly bind with the promoters of claudin‐1 and ZEB1 to modulate their transcription, suggesting both direct and indirect regulation of EMT by Sox9.^102^

## 5 SIGNALINGS IN EMBRYONIC DEVELOPMENT LINK TO EMT

It is well acknowledged that carcinogenesis and embryonic development share remarkable similarities. A series of features of embryogenesis, including EMT, angiogenesis, ECM remodeling, cell differentiation, and migration, are also important hallmarks of cancer. Indeed, EMT is fine‐tuned during embryonic development for morphogenesis of organs, whereas tumor cells hijack this program for cancer progression. Therefore, tumor is to some extent considered a problem in the field of developmental biology. For instance, proregenerative glia progenitors perform spinal cord repair via EMT in response to spinal cord injury in zebrafish, whereas dysregulated brain development such as excessive interneuron generation promotes the formation of brain tumors in human.^307^, ^308^ Aforementioned EMT‐TFs that widely studied in the context of neoplasm, actually play critical roles in embryonic development. For example, Snail and PRRX1a govern the internal left–right (L/R) asymmetry, which is fundamental to the proper function of organs (e.g., the heart laterality) during the development of vertebrates.^309^, ^310^ Generally, embryonic development is regulated by several evolutionarily conserved signaling pathways, including TGFβ, Wnt, Hedgehog, and Hippo.^311^, ^312^, ^313^, ^314^ These signalings have to be restricted when developmental processes are completed, which is the prerequisite for the proper morphology and function of organisms. Aberrant reactivation of these pathways in a well‐mature organ, however, frequently contributes to tumor development. Compelling evidence indicate that EMT program in cancer cells are largely regulated by embryogenesis‐related signalings, suggesting EMT as an intrinsic link between embryonic development and tumor progression.

### 5.1 TGFβ

**FIGURE 4:**
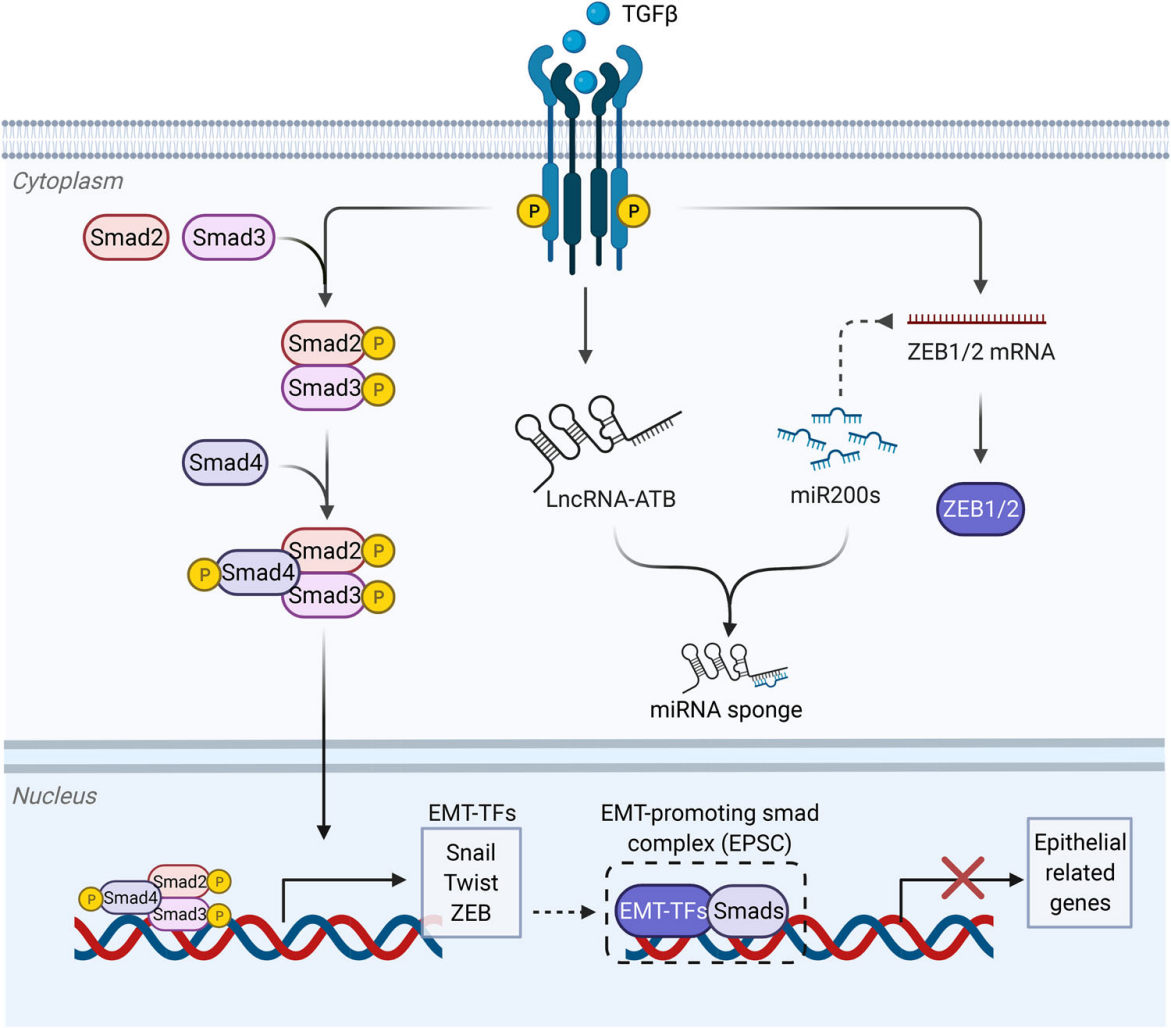
The crosstalk between EMT and TGFβ signaling. Activated TGFβ signaling leads to the nuclear translocation of Smad2/3/4 complex, which directly recognizes the promoters of EMT‐TFs to initiate their transcription. In turn, EMT‐TFs can bind with Smad proteins to form EMT‐promoting Smad complex (EPSC), thus regulating the transcription of epithelial and mesenchymal marks. Besides, TGFβ signaling also regulate the expression of EMT‐TFs through noncoding RNAs

TGFβ signaling is activated by the interaction between TGFβ ligands and receptors (type I and type II) on the cell membrane. Next, type I receptor is phosphorylated by type II receptor, which then phosphorylates Smad proteins, the key transcription factors mediating the biological consequence of this signaling. There are eight members in Smad protein family with different roles. Briefly, Smad1/2/3/4/5/8 positively regulate TGFβ signaling through the formation of activated Smad complexes, which translocate into nucleus to initiate gene transcription. In contrast, Smad6/7 negatively regulate TGFβ signaling via preventing the formation of activated Smad complexes and facilitating the degradation of TGFβ receptors.^315^ In the context of neoplasm, although TGFβ signaling can be both oncogenic and tumor suppressive, a variety of tumors benefit from activated TGFβ signaling, and targeting TGFβ signaling can be a promising therapeutic strategy for cancer treatment.^316^, ^317^ One of the mechanisms is that TGFβ signaling regulates EMT program of tumor cells, thus promoting cancer progression (Figure 4). For example, TGFβ–Smad signaling has been shown to activate the expression of Snail1, leading to EMT and proliferation of lung cancer cells.^24^ Not surprisingly, Smad proteins play central roles in TGFβ‐mediated EMT program. Indeed, Smads physically associate with EMT‐TFs such as Snail, Twist, ZEB to form an EMT‐promoting Smad complex (EPSC; e.g., Snail1–Smad3/4 complex), which represses the transcription of epithelial marks while increasing the expression of mesenchymal marks.^224^, ^318^ Besides, TGFβ induces long noncoding RNA (lncRNA)‐ATB, which acts as a competing endogenous RNA (ceRNA) to competitively bind with miR‐200s. This effect increases the expression of ZEB1/2, leading to EMT and metastasis of liver cancer.^319^ In contrast, several tumor suppressors exhibit their antimetastatic function through inhibiting TGFβ signaling, including circular RNA circPTK2 and lncRNA SMASR.^320^, ^321^ Interestingly, TGFβ signaling has been also demonstrated tumor‐suppressive via a lethal EMT.^322^ This observation is in line with the context‐dependent roles of TGFβ signaling.

### 5.2 Wnt

**FIGURE 5:**
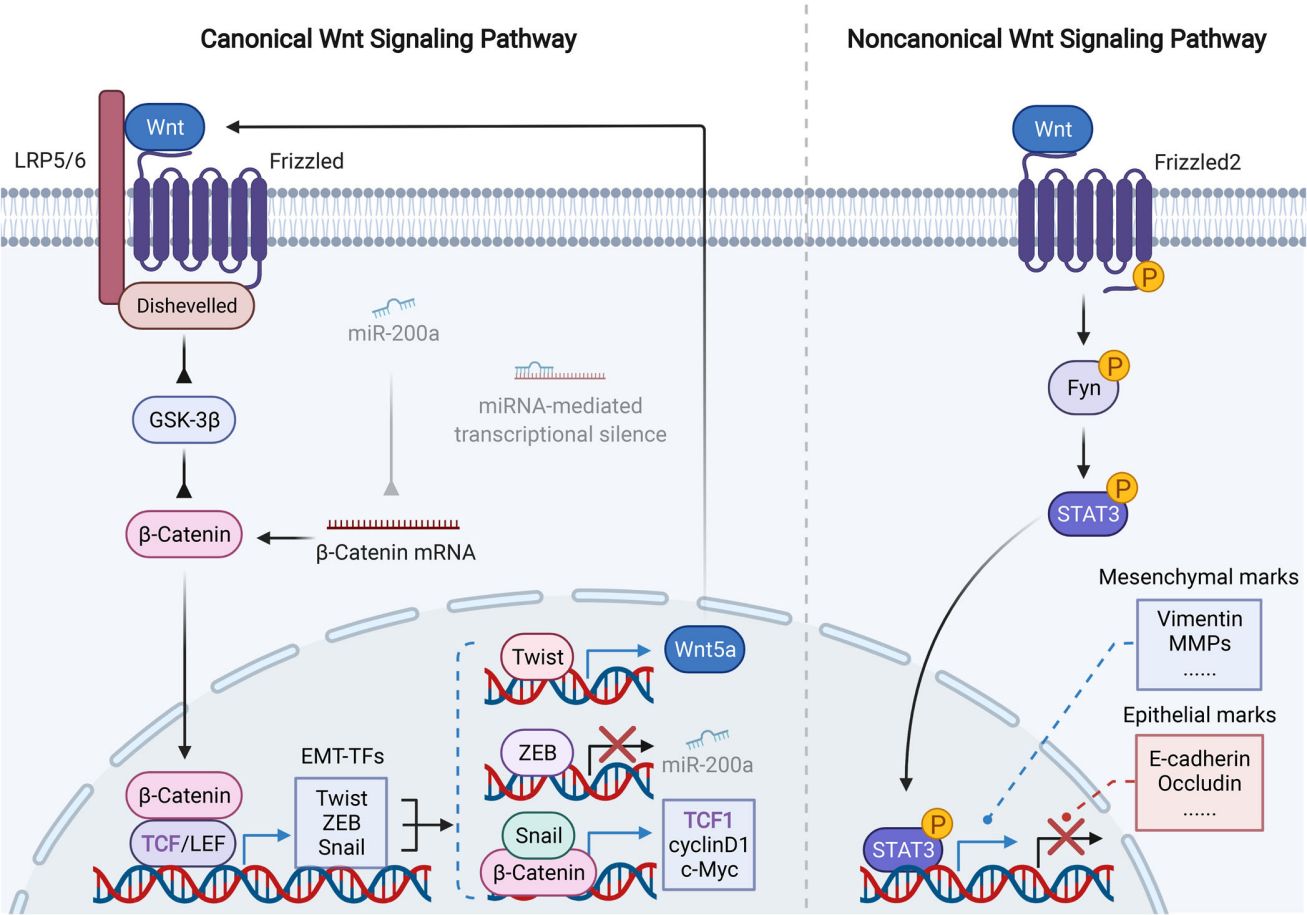
Regulation of EMT via canonical or noncanonical Wnt pathway. In canonical Wnt pathway, nuclear β‐catenin/LEF/TCF transcriptional complex activates the transcription of EMT‐TFs, thus regulating EMT. In turn, EMT‐TFs affect Wnt signaling to form feedback loops. For example, Snail/β‐catenin complex activates transcription of TCF1, which is the key transcription factor of Wnt signaling. ZEB represses the transcription of miR‐200a, leading to the reactivation of β‐catenin. Twist can transcribe Wnt5a, which activates Wnt receptor Frizzled. In noncanonical Wnt pathway, activation of Frizzled2 leads to the phosphorylation and consequent nuclear translocation of STAT3, which upregulates mesenchymal marks and represses epithelial marks independent of β‐catenin

Wnt signaling exert its biological effects mainly through the control of transcription cofactor β‐catenin. Briefly, in the quiescent state, β‐catenin is ubiquitinated and inhibited by GSK‐3β complex. When activated, Wnt proteins bind with the Frizzled receptor, leading to the recruitment of coreceptor LRP5/6. This event leads to the activation of Dishevelled (Dvl), which inhibits GSK‐3β thus protecting β‐catenin from degradation. Then, stabilized β‐catenin translocates into nucleus, where it interacts with transcription factor LEF/TCF to activate transcription.^323^, ^324^, ^325^ In terms of oncology, Wnt signaling is overall oncogenic, which allows malignant proliferation and metastasis of cancer cells, and this process involves the induction of EMT program^326^ (Figure 5). For instance, Her2 positive early disseminated cancer cells can enter into a partial EMT state via activation of Wnt signaling, thereby initiating metastasis in breast cancer.^327^ Mechanistically, the EMT‐promoting function of Wnt pathway is largely attributed to β‐catenin/LEF/TCF‐mediated transcription control. This is supported by the fact that β‐catenin/TCF4 can bind to the promoter of ZEB1 and increase its transcription, leading to the EMT and metastasis of colorectal cancer.^328^ Similarly, β‐catenin/TCF3 and β‐catenin/LEF1 interact with and activate the promoter of Snail and Twist, respectively.^329^, ^330^, ^331^ As a consequent, Snail can physically associated with β‐catenin to form a transcription complex, which activates Wnt target genes independent of TCFs.^332^ In contrast, autophagic degradation of β‐catenin has been shown to inhibit EMT and abrogate the metastasis of colorectal cancer.^333^ Interestingly, Wnt signaling can also promote EMT through a noncanonical way independent of β‐catenin. For example, the Wnt receptor Frizzled2 drives EMT and tumor metastasis in liver cancer via Fyn and Stat3, and this process is not affected by pharmacological inhibition of β‐catenin.^27^ In addition, EMT‐TFs can in turn activate Wnt signaling, forming a positive feedback loop to proceed EMT program. It has been shown that ZEB1 can repress the expression of miR200A, leading to the reactivation of β‐catenin.^334^, ^335^ Besides, Twist binds with the promoter of Wnt5a to increase its transcription, which activates Wnt signaling thus promoting breast cancer metastasis.^336^

### 5.3 Hedgehog

**FIGURE 6:**
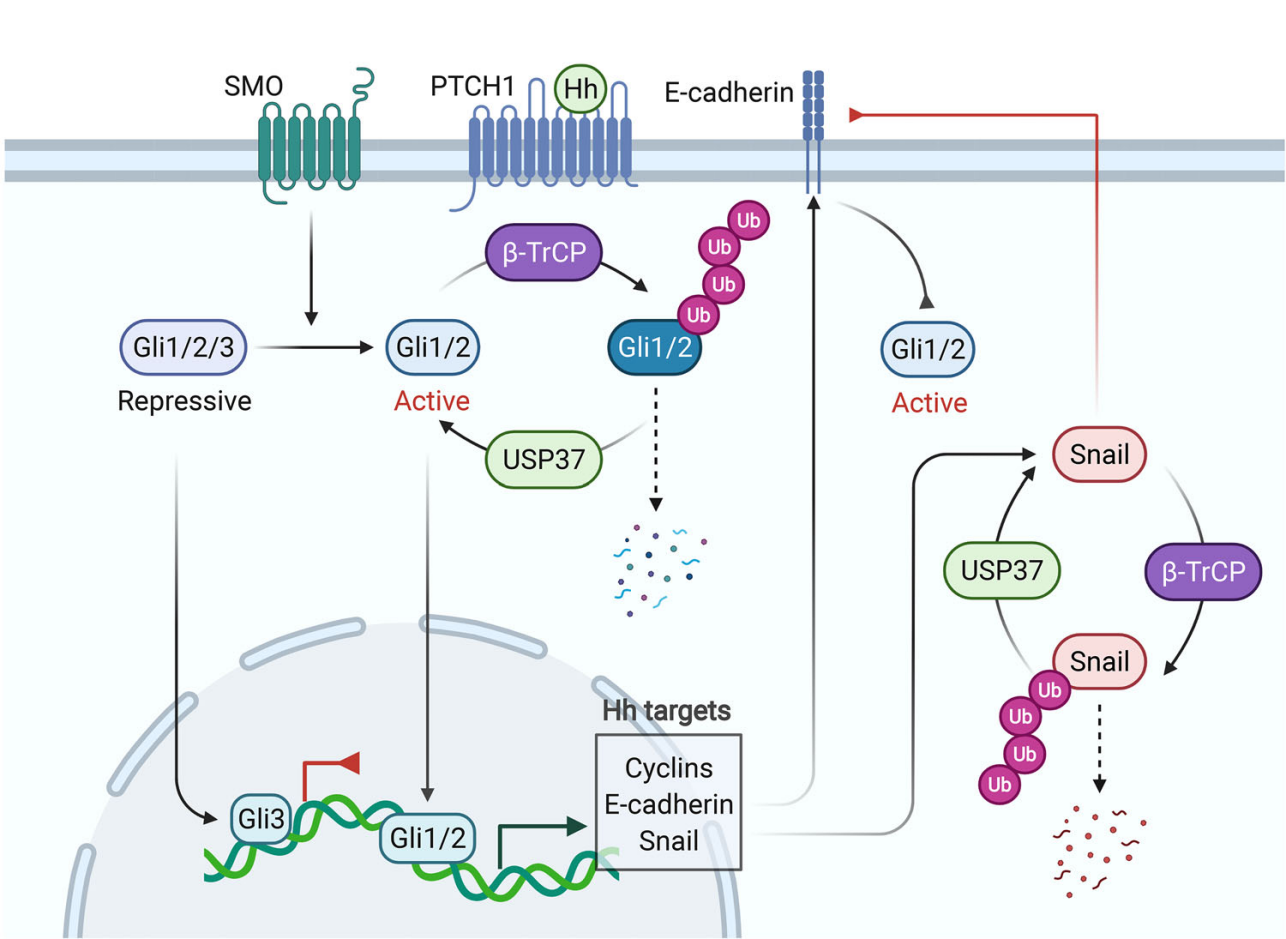
Hedgehog signaling regulates EMT and cancer progression. Hedgehog (Hh)‐mediated inhibition of PTCH1 leads to the activation of SMO, which promotes the activation of the transcription factor Gli1/2. The target genes of Gli1/2 include several EMT associated proteins such as Snail and E‐cadherin. Besides, Snail and Gli share the same protein turnover system, namely β‐TrCP‐mediated protein degradation and USP37‐induced protein stabilization. In turn, cellular EMT status affects Hedgehog signaling, as evidenced by loss of E‐cadherin has been shown to activate Gli1/2

Hedgehog signaling is activated by the binding of hedgehog ligands with the receptor Patched (PTCH1). This binding leads to the activation of membrane GPCR‐like protein Smoothened (SMO). In the absence of activated SMO, the transcription factor complex Gli1/2/3 is processed by proteasome, during which the active Gli1/2 is degraded, whereas the repressive Gli3 is preserved, resulting in transcriptional repression. When SMO is activated in response to Hedgehog signals, Gli1/2/3 is differentially processed to yield an active Gli1/2, which translocates into nucleus to initiate transcription.^337^, ^338^ Similar to other developmental pathways, Hedgehog signaling is tightly correlated with EMT program (Figure 6). For instance, aberrant activation of Hedgehog signaling promotes EMT of immature ductular cells, the accumulation of which results in fibrosis, cirrhosis and other liver diseases.^29^, ^339^, ^340^ In the field of oncology, Hedgehog signaling has long been implicated in cancer progression, at least partially due to its critical role in the regulation of EMT program of cancer cells.^341^, ^342^, ^343^ In human cholangiocarcinoma tissues, Hedgehog ligand is highly expressed to repress E‐cadherin, leading to a EMT phenotype and elevated viability of tumor cells.^344^ Besides, Hedgehog signaling‐mediated EMT, characterized by the overexpression of vimentin, Snail, N‐cadherin, and repression of E‐cadherin, ZO‐1, has been also reported in the metastasis of bladder cancer and breast cancer.^345^, ^346^ Inhibition of Hedgehog signaling with the administration of Vismodegib, the antagonist for PTCH1, is able to suppress EMT and cell proliferation of castration‐resistant prostate cancer.^347^ Compelling evidence suggest that Hedgehog signaling promotes EMT via the key transcription factor Gli. Indeed, the EMT‐TF Snail is a transcriptional target of Gli1.^241^ Stabilization of Gli1, which is mediated by the deubiquitinase USP37, has been shown to activate EMT and increase invasiveness in breast cancer.^348^ Interestingly, USP37 also catalyzes the deubiquitination of Snail.^349^ Moreover, both Gli and Snail can be ubiquitinated by E3 ligase β‐TrCP.^245^, ^350^ Therefore, EMT program and Hedgehog signaling are connected via sharing protein turnover machinery. In turn, loss of E‐cadherin or activation of EMT‐TFs can activate Gli, thus probably forming a positive signaling circuit to sustain EMT phenotype.^351^, ^352^ Briefly, EMT‐TFs activate Six1, which stimulate Hedgehog signaling in neighboring tumor cells, but how E‐cadherin deficiency activates Gli remains elusive.^351^, ^352^ Although most literatures indicate that Hedgehog signaling positively regulates the EMT program and tumor progression, the contrary findings were also reported that describe that Gli1 binds with the promoter of E‐cadherin to activate its transcription, thereby inhibiting EMT.^353^ Besides, downregulation of Gli1 results in the disassembly of adherens junctions, leading to EMT and cell migration in pancreatic ductal adenocarcinoma.^353^ This observation suggests a context‐dependent role of Hedgehog signaling in EMT and cancer progression.

### 5.4 Hippo

**FIGURE 7:**
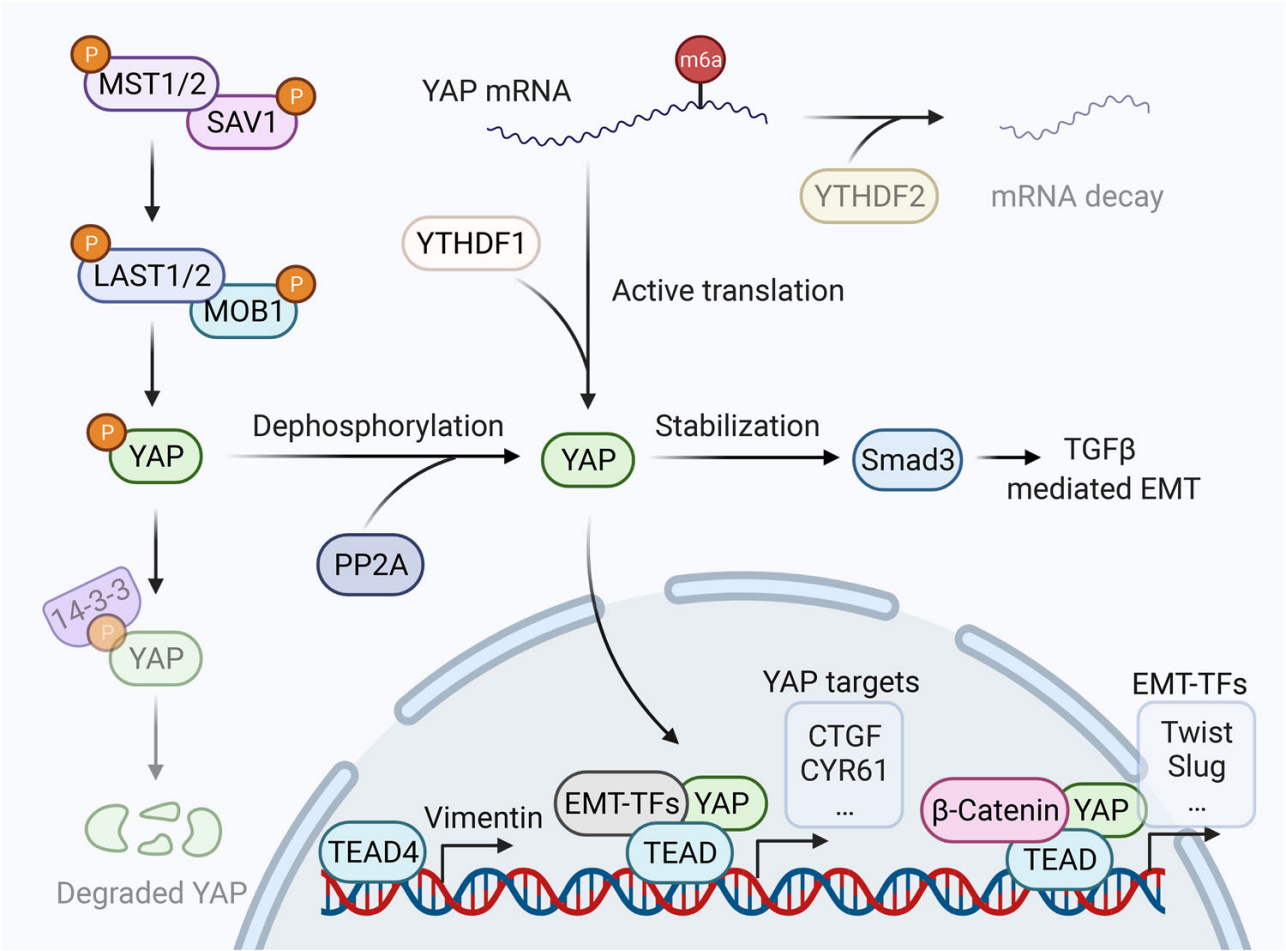
The connection between EMT and Hippo signaling. When Hippo signaling is inactivated, YAP and TEAD form complex in nucleus to direct transcription. EMT‐TFs can bind with YAP/TEAD complex, leading to a functional switch of EMT‐TFs from epigenetic repressors into activators, resulting in the overexpression of YAP target genes to facilitate tumor progression. Besides, Hippo signaling can regulate EMT via the interplay with other developmental pathways, such as TGFβ signaling and Wnt signaling. For instance, YAP can stabilize Smad3, the component of TGFβ signaling, thus promoting TGFβ‐mediated EMT. Besides, YAP/TEAD complex can be associated with β‐catenin to form a novel transcriptional complex, which activates the transcription of EMT‐TFs. Moreover, TEAD4 can transcriptionally activate the mesenchymal protein vimentin independent of YAP

The core of Hippo signaling is a kinase cascade that negatively regulates the activity of transcription cofactors YAP/TAZ. Given the oncogenic nature of YAP/TAZ, the Hippo signaling is generally regarded as a tumor‐suppressive pathway. Briefly, active Hippo signaling is characterized by phosphorylation of MST1/2–SAV1 complex, which phosphorylates LATS1/2–MOB1 complex. Then, phosphorylated LATS1/2 induce the phosphorylation of YAP/TAZ, leading to their cytoplasmic retention or ubiquitin‐mediated degradation. When Hippo signaling is inactivated, dephosphorylated YAP/TAZ can translocate into nucleus, where they interact with transcription factors TEAD1/2/3/4 to initiate transcription.^354^, ^355^ YAP/TAZ have been demonstrated potent EMT inducers promoting the progression of a variety of cancer (Figure 7). For instance, YAP is able to promote EMT and tumor metastasis by downregulating E‐cadherin and remodeling cytoskeleton in renal cancer and nasopharyngeal carcinoma.^356^, ^357^ Mechanistically, YAP interacts with several EMT‐TFs, including ZEB1, Snail, and Slug to form a transcriptional complex. This event results in a functional switch of EMT‐TFs from transcriptional repressors to transcriptional activators, which activates tumor‐promoting genes involved in EMT, tissue regeneration, and cancer metastasis.^216^, ^358^, ^359^, ^360^, ^361^ During EMT, the activation of YAP can be attributed to multiple mechanisms. For example, the catalytic subunit of protein phosphatase 2A (PP2Ac)‐mediated dephosphorylation of YAP facilitates YAP nuclear translocation, leading to EMT and metastasis of HCC.^362^ Besides, excessive formation of filamentous actin (F‐actin) leads to the dephosphorylation of LATS1, resulting in YAP stabilization and consequent liver cancer metastasis.^30^ Interestingly, YAP can either positively or negatively regulate the formation of F‐actin, suggesting this process is highly context‐dependent.^363^, ^364^ Besides, the activity of YAP can also be regulated at the RNA level. N6‐methyladenosine (m6A) modification on the YAP mRNA can be recognized by YTHDF1 and YTHDF2, which facilitates the translation and decay of YAP mRNA, respectively.^365^ Moreover, Hippo signaling can be cross‐linked with other developmental pathways, such as TGFβ and Wnt, to coordinate EMT program. It has been shown that YAP can prevent GSK3β‐mediated Smad3 degradation, thereby promoting Smad3‐induced EMT.^366^ Furthermore, YAP is physically associated with β‐catenin to form a transcriptional complex with TEAD4 in nucleus, leading to the overexpression of EMT‐TFs including Slug and Twist in breast cancer.^367^ It is worth noting that TEAD4 can regulate EMT and promote metastasis by directly transcribing vimentin without the binding with YAP in colorectal cancer, which is interesting.^368^

## 6 REGULATION OF EMT BY REDOX SIGNALING

In cancer cells, active metabolic patterns result in accumulation of reactive oxygen species (ROS), including hydroxyl free radicals, superoxide, and hydrogen peroxide, leading to oxidative stress. On one hand, ROS promote malignant transformation via inducing DNA damage and genomic instability, therefore being regarded as oncogenic molecules.^369^ Besides, ROS also sustain the proliferation of tumor cells and drive more aggressive phenotype.^370^, ^371^ However, excessive accumulation of ROS leads to cell death, suggesting that oxidative stress regulates cancer in contradictory ways.^372^ For example, oxidative stress can promote ferroptosis in melanoma to inhibit tumor metastasis, whereas administration of antioxidants has been shown to facilitate the metastasis of lung cancer.^373^, ^374^ In order to preserve rapid proliferation and aggressive behavior while avoiding death, tumor cells achieve redox equilibrium via increasing their antioxidant capacity. This process is enabled by the activation of antioxidant transcription factors (e.g., NRF2, NF‐κB, p53, FOXO, etc.),^375^, ^376^, ^377^, ^378^ the expression of antioxidant enzymes (e.g., SODs, CAT, PRDXs, TRXs, GPXs, etc.),^379^, ^380^, ^381^, ^382^, ^383^ and the production of small antioxidant molecules (e.g., GSH, vitamin C, vitamin E, etc.).^384^, ^385^, ^386^ Therefore, targeting oxidative stress is a potential anticancer strategy.^387^, ^388^, ^389^ Particularly, oxidative stress regulates cancer initiation and progression at least partially through modulating EMT program. For instance, ROS stimulate the expression of Snail, thus promoting EMT in breast cancer.^13^ Similarly, ROS accumulation induced by lipid peroxidation, GSH depletion, and SLC7A11 deficiency can initiate EMT in lung cancer cells.^390^ In consistence with these, treatment of antioxidants, such as N‐acetylcysteine (NAC), curcumin, resveratrol, has been shown to inhibit EMT in a variety of tumor cells.^391^, ^392^, ^393^ It is worth noting that ROS also promote MET in cancer cells. For example, 2‐deoxyglucose‐induced ROS accumulation promotes the phenotype transition of mesenchymal breast cancer stem cells (CSCs) into epithelial breast CSCs.^394^ This evidence suggests that EMT program is profoundly affected by cellular redox status.

**FIGURE 8:**
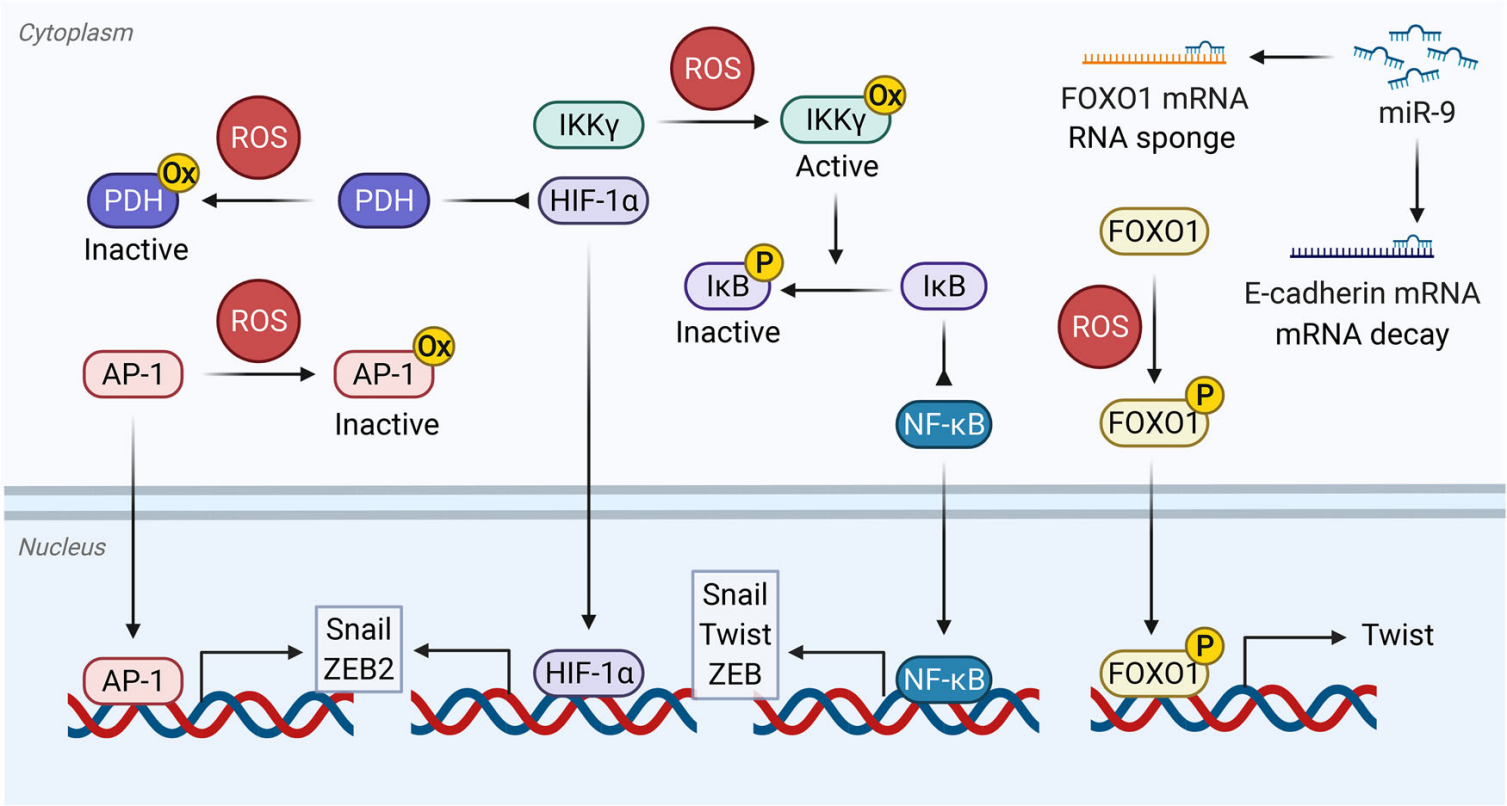
Regulation of EMT by oxidative stress and redox signaling. Redox signaling modulates EMT program via the regulation of redox sensors, including AP‐1, PHD, IKKγ, FOXO1, and many others. Briefly, these redox sensors can be either activated or inactivated via cysteine oxidation, thereby positively or negatively regulate the transcription of EMT‐TFs. Moreover, FOXO1 mRNA acts as ceRNA which binds with miR‐9, protecting E‐cadherin mRNA from miR‐9‐mediated degradation. This effect inhibits EMT program independent of EMT‐TFs

Mechanistic studies have revealed that ROS are not only toxic molecules that randomly cause damages, but also serve as secondary messengers to regulate signaling transduction.^395^ This is enabled by a number of ROS‐sensitive proteins, termed redox sensors.^396^ In response to the stimulation of ROS, certain cysteine residues on redox sensors can be oxidized at their sulfhydryl to generate cysteine sulphonate or to form disulfide bonds, leading to the conformational changes, formation of protein complex, and consequent acquisition of new biological functions.^397^ These processes can be reversed by antioxidant machinery, thus fine‐tuning cell behaviors called redox signaling.^398^ Compelling evidence suggest the critical roles of redox signaling in cancer progression. For example, redox modification of pyruvate kinase M2 (PKM2) at Cys358 causes a decrease of PKM2 enzymatic activity, leading to a metabolic reprogramming of cancer cells which supports tumor growth under oxidative stress.^399^ In terms of EMT program, redox signaling regulates the functions of junctions proteins, actin cytoskeleton, and EMT‐TFs, thereby affecting cancer progression (Figure 8).^400^ Aforementioned developmental pathways that control EMT program are actually crosslinked with redox signaling. For instance, TGFβ‐induced EMT can be enhanced or abolished by the treatment of hydrogen peroxide (H_2_O_2_) or NAC, respectively.^401^ Besides, ROS has been shown to activate Wnt signaling and subsequent EMT during wound healing.^402^ Furthermore, the Hippo component TAZ is a typical redox sensor, whose cysteines can undergo S‐glutathionylation in response to ROS.^403^ This redox modification improves the protein stability of TAZ, which facilitates its nuclear translocation and subsequent transcription of target genes.^403^ Together, these evidence suggest that redox signaling plays critical roles in EMT.

### 6.1 Redox regulation of cell junctions and cytoskeleton

ROS increase endothelial permeability through disrupting the integrity of cellular junctions between endothelial cells. For instance, ROS induced by the metabolic intermediate, 4‐hydroxy‐2‐nonenal, has been shown to modulate adherens junctions, tight junctions and integrins, leading to dysfunction of endothelial barrier.^404^ In line with it, another study showed that protein tyrosine phosphatase SHP2 is a critical factor preserving the integrity of endothelial barrier.^405^ In response to lipopolysaccharide (LPS)‐induced oxidative stress, SHP2 is oxidized at its Cys459, leading to its inactivation.^405^ This event activates Fyn‐related kinase, which phosphorylates several junction proteins, resulting in the disruption of endothelial adherens junction.^405^ Besides, ROS‐mediated endothelial permeability also involves the S‐glutathionylation of Rac1, a small Rho GTPase that regulates cytoskeleton.^406^ Metabolic stress‐induced S‐glutathionylation of Rac1 at Cys81 and Cys157 can inactivate Rac1, leading to the reorganization of cytoskeleton network and consequent vascular permeability in the aorta.^406^ Interestingly, β‐actin can be oxidized at Cys374, suggesting a direct regulation of cytoskeleton by redox signaling.^407^ In intestinal epithelial cells, the protein level of E‐cadherin can be decreased in response to TNF‐α‐induced oxidative stress, while its mRNA level is not changed.^408^ This observation suggests that ROS‐mediated loss of E‐cadherin might be at least partially independent of conventional epigenetic mechanisms by EMT‐TFs.^408^ In addition to E‐cadherin, many other EMT marks have been shown to be regulated by oxidative stress, including claudins, occludin, zonula occludens, α‐SMA, and vimentin.^409^, ^410^

### 6.2 Regulation of EMT‐TFs by redox sensors

As mentioned above, EMT‐TFs play crucial roles in EMT program. Though most EMT‐TFs do not seem to undergo direct redox modification, they are actually regulated by other redox sensors. The transcription factor, activator protein‐1 (AP‐1), is a typical redox sensor involved in EMT program. In response to oxidative stress, cysteines between its subunits are oxidized to form intermolecular disulfide bond, which decreases its DNA binding affinity.^411^, ^412^, ^413^ AP‐1 has been shown to directly bind with the promoter of Snail and ZEB2, thus activating their transcription, leading to EMT and metastasis of skin cancer, cervical cancer, and breast cancer.^414^, ^415^, ^416^, ^417^ Besides, AP‐1 can also physically interact with Snail and Twist, forming a transcriptional complex to induce EMT.^418^, ^419^ Another redox sensor, prolyl hydroxylase (PHD), can be inactivated by the oxidation of cysteines in its catalytic domain.^420^ This event leads to the activation of HIF‐1α, which transcribes ZEB1 and Twist to induce EMT and promote tumor metastasis.^421^, ^422^, ^423^ HIF‐1α can also improve the protein stability of Snail, thereby promoting cancer metastasis.^424^ In addition, the redox sensor IKKγ (also known as NEMO) can be activated via ROS‐induced disulfide bond formation between Cys54 and Cys347.^425^ Activated IKKγ phosphorylates IκB, leading to the dissociation between IκB and NF‐κB. This effect results in the activation of NF‐κB, which promotes the transcription of several EMT‐TFs.^426^ Moreover, FOX, class O (FOXO) proteins are a group of redox sensitive transcription factors, which play vital roles in cellular antioxidant defense.^378^, ^427^ In response to ROS, FOXO4 and transportin‐1 can form protein complex through intermolecular disulfide bond, promoting the nuclear translocation of FOXO4, which is required for the transcription of SOD2 and subsequent ROS elimination.^428^ Simultaneously, FOXO proteins are largely involved in cancer progression, notably the EMT program.^429^ For example, hypoxia‐induced oxidative stress promotes the phosphorylation and activation of FOXO1, which directly binds with the promoter of Twist to increase its transcription, leading to EMT and metastasis of prostate cancer.^430^ Besides, FOXO1 can also upregulate ZEB1 during EMT process.^431^ Another FOXO family member, FOXO3a, has been shown to inhibit β‐catenin via upregulating miR‐34 or directly protein binding.^432^ This effect abolishes the β‐catenin‐mediated transcription of ZEB1, thus repressing EMT in prostate cancer.^432^ Interestingly, FOXO proteins might regulate EMT independent of classical EMT‐TFs. For instance, FOXO1 mRNA serves as a competitive endogenous RNA (ceRNA), whose 3′UTR region can be targeted by miR‐9.^433^ This event protects E‐cadherin mRNA from miR‐9‐mediated degradation, resulting in maintenance of E‐cadherin expression thus inhibiting breast cancer metastasis.^433^

## 7 EMERGING ROLES OF EMT IN HALLMARKS OF CANCER

Cancer cells acquire distinct hallmarks in different stages during progression, including rapid proliferation, resisting cell death, deregulating cellular energetics, tumor‐promoting inflammation, acquiring stemness, inducing angiogenesis, activating metastasis, and many others.^434^ Among them, tumor metastasis is the canonical consequence of EMT program which has been widely reported. Even so, new concepts have also emerged in recent years. For instance, tumor cells in hybrid EMT state, but not completed mesenchymal state, possess highest metastatic capability.^108^ In line with this, expression of the epithelial mark E‐cadherin, which is conventionally thought to inhibit metastasis, actually promotes metastasis in some circumstances.^118^ Moreover, it has been reported that EMT is not required for metastasis.^115^, ^116^ This evidence indicate that EMT‐regulated tumor metastasis is highly context‐dependent. Here, we mainly focus on the contribution of EMT on other cancer hallmarks, including metabolic reprogramming, CSC property, and tumor‐promoting inflammation.

### 7.1 Metabolic reprogramming

Cancer cells undergo metabolic reprogramming to meet the energy and nutrient demand, which enables the sustaining proliferation in adverse condition. One of the well‐known metabolic reprograming is the “Warburg effect” describing that tumor cells utilize glycolysis rather than oxidative phosphorylation even in a normoxia condition, termed as “aerobic glycolysis.”^435^ During EMT process, tumor cells reprogram their glucose metabolism and lipid metabolism to maintain aggressive behaviors. Specifically, the key EMT‐TF Snail can regulate the transcription of enzymes in glucose metabolism. For instance, Snail represses the expression of phosphofructokinase PFKP, thus switching the glucose flux toward pentose phosphate pathway.^436^ Besides, Snail‐mediated silencing of fructose‐1,6‐biphosphatase induces glycolysis, which maintains stemness and invasiveness of basal‐like breast cancer cells.^437^ However, it has been also reported that metastatic cancer cells prefer oxidative phosphorylation to glycolysis, which might be due to the decease of glucose uptake and consequent energy crisis in CTCs.^438^ Therefore, tumor cells probably tend to utilize oxidative phosphorylation to achieve more efficient ATP production rather than glycolysis during EMT.^439^ This concept is supported by the finding that nuclear phosphoglycerate kinase 1 drives EMT and metastasis by oxidative phosphorylation in Smad4‐deficient pancreatic ductal adenocarcinoma cells.^440^ Notably, aberrant glucose metabolism in turn affects EMT through regulating EMT‐TFs, such as Snail. It has been shown that hyperglycemic condition induces an O‐GlcNAc modification at Ser112 on Snail1, thereby enhancing its protein stability to facilitate EMT program in breast cancer cells.^441^

Lipid metabolism is closely related with cancer metastasis, as evidenced by the finding that the metabolic shift toward fatty acid oxidation is critical for tumor metastasis to lymph nodes.^442^ Besides, the formation of lipid droplets has been found to promote metastasis of tumor cells.^443^ Given the direct connection between EMT and metastasis, it is not surprising that lipid metabolism plays vital roles during EMT program. Indeed, EMT‐like morphological changes require the alterations in membrane lipid composition, which is enabled at least partially through the fatty acid translocase CD36.^444^, ^445^ In addition, the formation of invadopodia, a classical EMT‐like morphological alteration, is induced by the reprogramming of lipid metabolism during the progression of liver cancer.^446^ Apart from regulation of membrane dynamics, lipid metabolism also regulates the fate of EMT cells through ferroptosis, a cell death form induced by lipid peroxidation. Cancer cells in completed mesenchymal state that lack of E‐cadherin expression largely suffer from severe oxidative stress, making them highly vulnerable to ferroptosis.^118^ Nevertheless, lymph protects those metastatic cancer cells from ferroptosis by reliving oxidative stress, which is dependent on the abundant oleic acid, a kind of unsaturated fatty acid.^373^ This evidence suggests that metabolism with different lipid substrates can diversely regulate ferroptosis, cell survival, and metastasis during EMT. Furthermore, different lipid metabolism enzymes also regulate metastasis in contrary ways. For example, the lipid metabolism enzyme ATP‐citrate lyase can promote the metastasis of colorectal cancer and liver cancer.^447^, ^448^ In contrast, another lipid metabolism enzyme, acyl‐CoA synthetase long‐chain family member 4, sensitizes cancer cells to ferroptosis by esterifying arachidonic acid and adrenic acid into phosphatidylethanolamine.^449^ According to the profound impacts of lipid metabolism in EMT and metastasis of cancer, targeting lipid metabolism can be a potential therapeutic strategy for cancer treatment. For instance, inhibiting lipid metabolism by simvastatin‐based nanomedicine has been shown to reverse EMT and overcome cancer drug resistance.^450^

### 7.2 Cancer stem cell property

The capacity of tumor initiation is partly driven by the activation of the EMT program because active EMT‐TFs confer numerous properties of stemness on those with cell plasticity. Indeed, aberrant expression of EMT‐TFs, such as Twist1, SIP‐1, and Snail, in cancer cells contributes to the acquisition of stem‐cell features, as evidenced by the increased self‐renewal ability in vitro and tumor propagation potential in vivo.^42^, ^437^, ^451^, ^452^ Moreover, ZEB1, as an EMT activator, has been identified to enhance tumorigenic and colonization capacity by stabilizing the stem cell marker Sox2 in a miR‐200c‐mediated manner in pancreatic cancer derived from a KPC model with mutant Kras and p53.^17^ Similar results have been demonstrated in glioblastoma, where ZEB1 contributes to tumor initiation by orchestrating a series of stemness effectors, including SOX2, OLIG2, and CD133/PROM1.^452^ Additionally, the results from breast cancer indicate that the EMT regulators ZEB1/2 and Bmi1 promote stemness toward tumorigenesis and metastasis via TET‐Family‐dependent epigenetic modulation. Mechanistically, miR‐22 induces chromatin remodeling by decreasing 5hmC levels by inhibiting the TET family, therefore leading to epigenetic silencing of miR‐200, which is involved in the expression of ZEB1/2 and Bmi1.^453^ Recently, a novel signaling axis, the basic helix–loop–helix (bHLH) transcription factor E2A with Snail1, was confirmed to participate in the maintenance of breast cancer stemness, facilitating tumor initiation, metastasis, and drug resistance.^454^ It has also been reported that deletion of the protocadherin FAT1 induces a hybrid EMT state in skin squamous cell carcinoma and lung tumors by triggering not only CAMK2–CD44–SRC axis‐mediated YAP1 activation and ZEB1 expression but also EZH2‐regulated SOX2 expression, thus resulting in maximal stemness and accelerated cancer progression.^114^

In addition to the involvement of tumorigenesis, EMT‐mediated stemness is also identified as a key driver of acquired drug resistance and subsequent tumor relapse after drug holidays.^35^ Accumulated evidence reveals that cancer cells in EMT activation seem to undergo dedifferentiation similar to stem cells, showing upregulation of antiapoptotic and drug efflux proteins.^455^, ^456^ For instance, multidrug resistance (MDR), especially to gemcitabine, in pancreatic cancer is closely correlated with the activation of the EMT‐like transcription factor ZEB1.^457^ In detail, solute carrier family 39 member 4, which functions to modulate the intracellular zinc concentration, induces the expression of the zinc‐dependent EMT protein ZEB1 by stimulating STAT3 activation. Consequently, ZEB1‐induced ITG A3/B1 triggers integrin α3β1 signaling and subsequent c‐Jun‐N‐terminal kinase signaling, therefore inhibiting the expression of gemcitabine transporter ENT1.^457^ Similarly, SRF/MRTF‐mediated signaling is involved in the mesenchymal transition of melanoma with RAC1P29S by upregulation of the EMT‐TFs Snai2 and Jun, driving a low apoptosis rate and BRAFi resistance.^458^

Of note, various cancer therapies, including chemo‐, radio‐, and immunotherapy, can also result in an EMT‐mediated stress adoption mechanism, which was recently defined as one feature of the drug‐tolerant persister (DTP) state.^459^ These residual cells with EMT characteristics govern the nongenetic mechanisms to enter the dormant state against a wide spectrum of antiproliferation treatments.^460^ For example, Matthew and colleagues indicate that high‐mesenchymal persister cancer cells derived from drug treatments hold more resistance to tyrosine kinase inhibitor therapy and chemotherapy.^461^ Furthermore, the features of EMT activation, upregulation of vimentin, and downregulation of E‐cadherin have also been examined in MDR persister cells, which develop DTP phenotypes after canonical MDR reversal treatment.^462^ Notably, observations in patients with breast cancer or mice with lung cancer suggest a strong underlying connection among EMT, stemness, and drug resistance by analyzing the expression of EMT markers in posttherapy specimens, showing an activated EMT program and elevated chemoresistance‐related proteins.^115^, ^463^

### 7.3 Tumor‐promoting inflammation

The progression of cancer is largely dependent on the interaction between tumors and tumor microenvironment, especially local inflammatory status. Inflammatory microenvironment is composed by a variety of cells, including tumor cells, stromal cells, tumor‐associated macrophages (TAM), cancer‐associated fibroblasts, myeloid‐derived suppressive cells, T cells, monocytes, dendritic cells, adipocytes, and so on. Inflammatory response is required for immunosurveillance to kill cancer cells; however, it can also promote cancer progression. For example, macrophages can be polarized into M2 tumor‐promoting ones. During cancer progression, EMT program can regulate the function of inflammatory and immune cells, whereas several inflammatory cytokines are potent inducers of EMT.^45^ As such, EMT program and inflammatory response are affected by each other thus forming complex network, which leads to cancer regression or progression.

Mounting evidence indicate that EMT can be regulated by inflammation. For instance, pancreatitis has been found to promote EMT of premalignant pancreatic epithelial cells, leading to their dissemination to liver, and this process can be abolished by the administration of immunosuppressive agent dexamethasone.^464^ Generally, inflammation‐induced EMT is largely attributed to proinflammatory cytokines, such as NF‐κB, interleukins (ILs), interferons, chemokines, and others. The level of IL‐22 is increased during pancreatitis, which promotes EMT of pancreatic ductal adenocarcinoma cells and subsequent tumor progression.^465^ Another IL, IL‐6, facilitates the transformation of normal liver stem cells into metastatic CSCs by inducing EMT.^466^ Interestingly, IL‐35 derived from M2 TAMs has been reported to promote MET of metastatic tumor cells, thus resulting in distant colonization.^467^ Similar tumor‐promoting effects were also observed in other proinflammatory factors, such as M2 TAM‐derived chemokine CCL‐22 and natural killer cell‐derived interferon IFN‐γ, both of which promote the progression of liver cancer via induing EMT.^468^, ^469^ Mechanistically, these cytokines might regulate the expression of EMT‐TFs, thus affecting EMT program. For instance, the pro‐inflammatory cytokine NF‐κB is able to inhibit the ubiquitination and degradation of Snail, thereby mediating inflammation‐induced tumor metastasis.^248^

In contrast to the large amount of literature describing inflammation‐regulated EMT, the investigation of EMT‐regulated inflammation and immune response is rather limited. It has been shown that EMT can change the antigens present in tumor surface, recruit M2 TAMs instead of M1 macrophages, thus contributing to the immunosuppression in breast cancer, and this process is probably enabled by the release of cytokine granulocyte‐macrophage colony‐stimulating factor.^470^, ^471^ Besides, the EMT‐TF ZEB2 can regulate cytokine signaling thus playing critical role in hematopoietic differentiation and myeloid diseases.^276^ Interestingly, tumor cells undergoing EMT have been reported to increase susceptibility to natural killer cells, which enhance immunosurveillance to inhibiting metastasis in lung cancer.^472^ This observation suggests that inflammatory or immune responses are not always tumor promoting, but can provide beneficial for cancer patients in some circumstances if not being hijacked by tumor cells.

## 8 EMT‐BASED CLINICAL APPLICATIONS IN ONCOLOGY

### 8.1 Clinical diagnosis by identifying EMT markers

Given the crucial role of the EMT program in tumorigenesis and development, the identification of EMT‐involved biomarkers has accordingly emerged as a diagnostic approach, which is used to match personalized therapies or adjust the treatment intensity. Indeed, an increasing number of clinical cases have indicated that EMT multi‐marker combination analysis by immunohistochemistry or immunofluorescence is a key part of tumor stratification or companion diagnostics for patients with different tumor types.^473^, ^474^ (Table 1)

**TABLE 1:** Implications of several EMT markers in the diagnosis of cancer

EMT markers	Sample types	Synergetic markers	Clinical symptoms	Cancer types
miR‐200c	Tissue biopsy	ZEB1, E‐cadherin, and vimentin	Liver metastases	Colorectal cancer
ZEB1	Tissue biopsy	CDH11, MMP2	Invasive disease	Breast cancer
E‐cadherin	Tissue biopsy	N‐cadherin, vimentin	Biochemical recurrence	Prostate cancer
β‐catenin	Tissue biopsy	E‐cadherin, vimentin, CD133, CD44v6, and so on	Metastases and therapy adaptation	Prostatic, colorectal, breast cancer, and so on
Twist1	Liquid biopsies	Akt2, PI3Kα, and ALDH1	Metastases and therapy adaptation	Breast cancer
PI3Kα and/or Akt‐2	Liquid biopsies	ALDH1	Metastases and therapy adaptation	Colorectal cancer

In colorectal cancer, miR‐200c, a known EMT mediator that has been investigated in preclinical models, is examined in 45 pairs of primary tumor tissues and corresponding matched liver metastases. Expectedly, altered expression of miR‐200c, which has low expression in the invasive front of primary cancer tissues and high expression in liver metastasis tissues, showed a strong association with various EMT‐related proteins, such as ZEB1, E‐cadherin, and vimentin, suggesting a potential metastatic indicator for patients.^475^ In line with this, the results from a longitudinal follow‐up study based on 185 stage II/III colorectal cancer patients solidly demonstrated a relationship between a high combined EMT score and poor prognosis.^476^ Furthermore, several EMT genes, including CDH11, MMP2, and ZEB1, have been identified with higher expression in invasive breast cancer than in pure ductal carcinoma in situ, indicating that EMT activation is related to a high risk of invasive disease in all subtypes of breast cancer, especially in ER‐negative disease.^477^ A study investigating prostate cancer patients treated pre‐ and postradiotherapy revealed that downregulation of E‐cadherin combined with upregulation of N‐cadherin and vimentin was correlated with biochemical recurrence, providing an independent predictive factor for treatment response to radiotherapy.^478^ Recently, Navas and colleagues proposed a validated, high‐resolution digital microscopic immunofluorescence assay for assessing the EMT phenotype of various human tumor specimens. Their study revealed that β‐catenin+ cancer cells accompanied by the induction of E‐cadherin and vimentin exist in various metastatic patients. Notably, the expression of stemness markers, such as CD133, CD44v6, ALDH1, and NANOG, is also examined in some cases, implying a standardized assessment for clinical monitoring of EMT‐mediated therapy adaptation.^474^

In addition to tissue biopsy, liquid biopsies are another promising method for clinical diagnosis and can also be used to analyze EMT characteristics of tumors, mainly CTCs from the blood of cancer patients. For example, a follow‐up study comprising 226 blood samples of 39 patients with metastatic breast cancer showed that the expression rates of EMT‐related proteins and the stemness marker ALDH1 were 62 and 44%, respectively, in CTCs of patients with no response to therapy. In contrast, the expression rates in responders are 10 and 5%, respectively.^479^ Likewise, through investigating related protein expression in CTCs isolated from blood, Ning et al. demonstrated that the coexpression pattern of EMT markers (PI3Kα and/or Akt‐2) and stemness markers (ALDH1) predicts significantly shortened progression‐free survival and overall survival (OS) in metastatic colorectal cancer patients.^480^ To date, the clinical trials of CTCs with EMT features are ongoing or have already been activated for diagnosing or treating patients with breast, prostate, pancreatic, and colorectal cancers (NCT04265274, NCT04021394, NCT04323917, and NCT04323813, respectively).

### 8.2 Therapeutic interventions targeting the EMT program

With the increasing in‐depth knowledge of detailed mechanisms modulating the EMT program, the identification of novel agents targeting this program has been an irreversible trend. Thus far, three reasonable strategies aimed at preventing the initiation of EMT activation, selectively eliminating mesenchymal‐like cancer cells, and reversing the EMT process by inducing the MET program all seem to be promising for controlling EMT‐mediated tumorigenesis and therapeutic tolerance. However, which approach is the best one with long‐term efficacy in the clinic remains controversial. In this section, we present various EMT‐targeted drugs based on the approaches mentioned above and list the related clinical trials (Table 2).

**TABLE 2:** Cancer therapeutic strategies based on targeting EMT

Drugs or combination	Targets or pathways	Cancer types	Intervene strategies	Clinical trials
Galunisertib, gemcitabine	TGFβ signaling	Pancreatic cancer	Inhibiting EMT induction	NCT01373164
Galunisertib, sorafenib	TGFβ signaling	Hepatocellular carcinoma	Inhibiting EMT induction	NCT01246986
Antisense	TGFβ2 mRNA	Gliomas	Inhibiting EMT induction	NCT00431561
Tepotinib, cetuximab	HGFR (Met)	Colorectal cancer	Inhibiting EMT induction	NCT04515394
Tepotinib	HGFR (Met)	NSCLC, hepatocellular carcinoma	Inhibiting EMT induction	NCT01982955, NCT01988493
Capmatinib	c‐Met	Glioblastoma multiforme, colorectal cancer, and so on	Inhibiting EMT induction	NCT02386826
Cabozantinib	AXL	Several solid tumors	Inhibiting EMT induction	NCT03170960
ADH‐1	N‐cadherin	Several solid tumors	Killing cells undergoing EMT	NCT01825603, NCT00421811
Pritumumab	Vimentin	brain cancer	Killing cells undergoing EMT	NCT04396717
Mocetinostat, nivolumab	HDAC‐mediated ZEB1	NSCLC	Killing cells undergoing EMT	NCT02954991
All‑trans retinoic acid	Activation of MET	Breast cancer, head and neck cancer, neuroblastoma	Reversing the EMT process	NCT05016349, NCT03370367, NCT03042429
Tazemetostat	EZH2	Prostate cancer, lymphomas, and so on	Reversing the EMT process	NCT04179864, NCT01897571

#### 8.2.1 Strategies to inhibit EMT induction

Intervention with TGFβ signaling, the confirmed pathways involved in EMT induction by several preclinical studies, is currently the most recognized strategy. Galunisertib (LY2157299) is a small molecule inhibitor targeting TGFβR1 and has been investigated in various advanced tumors.^481^, ^482^, ^483^ Importantly, the anticancer effect of galunisertib is at least partially attributed to its capability of inhibiting EMT. For example, galunisertib was revealed to show a marked sensibilization to enzalutamide treatment in prostate cancer due to that galunisertib‐mediated TGFβ signaling inhibition prevented EMT process.^481^ To date, more than 10 clinical trials of galunisertib have been completed for treating metastatic or recurrent cancer (https://clinicaltrials.gov/). Among them, a clinical trial for advanced or metastatic unresectable pancreatic cancer suggests that galunisertib combined with gemcitabine has a promising anticancer effect on patients, as evidenced by the prolonged OS (NCT01373164).^484^ Similarly, the therapeutic effect of galunisertib combined with sorafenib was confirmed in a phase II study regarding advanced hepatocellular carcinoma, showing acceptable safety and a pronounced improvement in OS (NCT01246986).^485^ Notably, there also exist many similar small molecule inhibitors depending on TGFβR1 inhibition, such as EW‐7195, LY580276, and SD‐208, displaying potential anticancer effects by blocking TGFβ pathways.^486^ In addition to small molecular inhibitors, antisense therapy targeting TGFβ2 mRNA has been revealed to have comparable effects, and related clinical trials have been ongoing (NCT00431561).^487^ Nonetheless, it will be counterproductive to perform a blind targeting of TGFβ signaling, which has multifaceted biological functions in tumorigenesis and development.^488^ Indeed, in the early stages of cancer, induction of TGFβ signaling actually limits the proliferation of tumor cells.^489^ Thus, rational patient stratification and optimized drug administration will be key to the effective use of these therapeutic regimens.

The HGF–HGF receptor (also known as Met) axis is another well‐known mediator of EMT induction.^490^ Excessive activation of the signaling axis is often attributed to point mutation or amplification of the HGFR gene, therefore triggering EMT‐induced cell motility and conferring resistance to a series of anticancer agents in cancer.^491^ As such, substantial efforts have been made to develop HGF‐HGFR signaling inhibitors; furthermore, an increasing number of clinical studies are evaluating the anticancer effect of c‐Met inhibitors in different types of cancer.^492^ For example, scientists from the EMD Serono Research & Development Institute recently combined the selective c‐Met inhibitor tepotinib with cetuximab for treating metastatic colorectal cancer in a Phase II clinical trial (NCT04515394). Moreover, the effects of tepotinib were also identified in patients with EGFR‐mutant non‐small‐cell lung cancer (NCT01982955)^493^ or with advanced hepatocellular carcinoma (NCT01988493),^494^ as observed by improved anticancer activity and time to progression, respectively. Capmatinib, another selective c‐Met inhibitor, has also been approved for treating Met exon 14‐altered non‐small‐cell lung cancer.^491^ Furthermore, a phase I clinical trial of capmatinib is ongoing for the treatment of glioblastoma multiforme, gliosarcoma, colorectal cancer, and renal cell carcinoma (NCT02386826).

In addition, the potential therapeutic agents for blocking EMT induction include other target inhibitors, such as COX‐2 inhibitors and AXL inhibitors. The COX‐2 selective antagonist celecoxib was revealed to prevent EMT‐mediated malignant transformation by modulating β‐catenin nuclear localization, the vimentin/E‐cadherin proportion and EMT‐TFs (Slug, Snail and ZEB1) in colorectal cancer cells and oral squamous cell carcinoma.^495^, ^496^ Additionally, inhibition of AXL by the multikinase inhibitor cabozantinib reverses EMT‐associated drug resistance in renal cell carcinoma and non‐small‐cell lung cancer.^497^, ^498^ Since 2017, the City of Hope Comprehensive Cancer Center has been conducting a phase I clinical trial of combination therapy of cabozantinib and atezolizumab, treating more than 10 different types of advanced or metastatic solid tumors (NCT03170960). Recently, their study reported that cabozantinib combined with atezolizumab showed encouraging efficacy and acceptable tolerability in advanced clear cell and nonclear cell renal cell carcinoma.^499^

#### 8.2.2 Strategies to kill cells undergoing EMT

Compared with prevention of EMT induction, a promising alternative strategy is to selectively target EMT‐induced mesenchymal‐like cancer cells by therapeutically inhibiting the functions of EMT‐specific markers. For example, the novel pentapeptide ADH‐1 was identified as an N‐cadherin antagonist, which breaks extracellular N‐cadherin adhesion, therefore disturbing the interaction with FGFR‐1 and accelerating the degradation of FGFR‐1. ADH‐1 treatment markedly enhances the cytotoxicity of chemotherapy.^500^ Notably, the clinical applications of cisplatin, gemcitabine hydrochloride, or melphalan combined with ADH‐1 have been completed and display considerable promise (NCT01825603 and NCT00421811). Furthermore, vimentin, another classic EMT marker, has been revealed as the direct target of withaferin A, which reduces EMT‐associated cancer metastasis by triggering the degradation of vimentin intermediate filaments.^501^, ^502^ Pritumumab, a natural IgG1κ antibody derived from cervical carcinoma patients, is also an antagonist of vimentin and was originally evaluated in a clinical trial of patients with brain cancer in Japan.^503^ Recently, a clinical trial of pritumumab in brain cancer therapy has been conducted again, implying promising clinical therapeutic potential (NCT04396717). Beyond the direct suppression of these EMT effectors, targeting EMT‐TFs is an alternative approach against EMT‐induced mesenchymal‐like carcinoma. Indeed, inhibition of the EMT activator ZEB1 by the HDAC inhibitor mocetinostat has been shown to impede drug resistance to oncotherapy in lung and pancreatic cancer.^504^, ^505^ Moreover, a phase II clinical trial in which mocetinostat was combined with nivolumab for treating advanced or metastatic non‐small‐cell lung cancer is ongoing (NCT02954991). Similarly, pharmacologic inhibition of another EMT‐TF, Snail, can also prevent EMT‐derived malignancy. Lee and colleagues provided a small chemical inhibitor named GN25 and GN29, which showed significant anticancer effects by specifically breaking p53‐Snail binding in KRAS‐driven human cancer.^506^

Importantly, EMT‐driven mesenchymal phenotype of cancer cells seems to hold unique therapeutic vulnerabilities. For example, the aforementioned study reports that mutation‐induced FAT1 function defects result in Hippo signaling inhibition and ZEB1 expression by activating the CAMK2‐CD44‐SRC axis, thus conferring mesenchymal state‐related stemness and metastasis. Interestingly, compared to FAT1 wild‐type cancer cells, the loss of FAT1 dramatically enhances the drug sensitivity of cancer cells to dasatinib, saracatinib (SRC inhibitors), and KN93 (CAMK2 inhibitor).^114^ Similarly, Kosuke et al. indicated that treatment with the EGFR inhibitor osimertinib causes acquired resistance by promoting the EMT process in lung cancer. These cells with a mesenchymal‐like phenotype activate the ATR–CHK1–AURKB axis, simultaneously showing obvious therapeutic vulnerability. AURKB inhibitors and ATR/CHK1 inhibitors.^507^ Additionally, the sustainment of endoplasmic reticulum (ER) homeostasis and redox balance is also confirmed as an indispensable requirement for EMT subpopulation survival in various types of cancer, suggesting that ER or oxidative stress inducers can selectively kill these clusters.^394^, ^508^ Recently, a novel strategy based on cell plasticity, aiming to force trans‐differentiation of EMT‐related breast cancer cells into postmitotic adipocytes, seems to work. Ronen and colleagues combined rosiglitazone (antidiabetic drug) with trametinib (MEK inhibitor) to suppress the metastasis of breast cancer.^509^ With the in‐depth understanding of EMT‐driven characteristics, high‑throughput screening approaches have been used to identify therapeutic vulnerabilities in carcinoma cells with EMT‐induced phenotypes.^510^, ^511^, ^512^ Nevertheless, the clinical application of a similar strategy for treating EMT‐mediated metastasis and drug resistance still requires further exploration and validation.

#### 8.2.3 Strategies to reverse the EMT process

Since EMT induction leads to elevated metastatic potential and stemness, activation of MET, a reverse process of EMT, would be a reasonably alternative therapeutic option. This strategy, by forcing differentiation of mesenchymal‐like cancer cells into regain epithelial features, resembles “differentiation therapy” treated for AML, whereby all‑trans retinoic acid treatment breaks the poorly differentiated state of AML with potential EMT traits, eventually resulting in differentiation‐mediated apoptosis.^217^, ^513^, ^514^ Notably, all‑trans retinoic acid treatment for solid cancers has been universally used in preclinical and clinical studies. In breast cancer cells and paclitaxel‐resistant colorectal cancer cells, all‑trans retinoic acid has been shown to reverse the EMT process, thus decreasing the motility of cancer cells both in vitro and in vivo.^515^, ^516^ Furthermore, all‑trans retinoic acid also displays dramatic anticancer potential in phase II and phase III clinical trials of various cancer patients (NCT05016349, NCT03370367, and NCT03042429).

In addition to retinoic acid, which removes EMT‐modulated malignant phenotypes by directly inducing differentiation, several epigenetic‐associated drugs have been identified as potential inducers of the MET program in preclinical models by preventing EMT activation‐mediated metastasis or drug resistance.^517^ Pattabiraman et al. indicated that the intracellular second messenger adenosine 3′,5′‐monophosphate induces epigenetic reprogramming by triggering the activation of protein kinase A and the subsequent phosphorylation of histone demethylase PHF2. Thus, PHF2 decreases histone methylation in mesenchymal cancer cells, in turn inducing a MET process and compromising tumor initiation, implying promising clinical therapeutic potential of inhibitors of histone methyltransferases.^518^ Indeed, tazemetostat, a kind of EZH2 histone methyltransferase inhibitor, has rapidly progressed into Phase II or Phase III clinical trials (NCT04179864 and NCT01897571). Additionally, epigenetic regulation‐mediated reprogramming of metabolic patterns in cancer cells can control cell state transitions. For example, Loo and colleagues reported that rechanneling fatty acid β‐oxidation toward lipid storage by retinoids reverses the mesenchymal‐like phenotype to an epithelial‐like phenotype in breast cancer, accompanied by a loss of EMT‐driven metastasis ability. Mechanistically, fatty acid β‐oxidation in the mesenchymal cell state exhibits epigenetic controls of EMT genes via upregulation of acetyl‐CoA‐dependent histone acetylation.^519^ In particular, morphological screening based on an organoid platform from a recent study has identified multiple class I HDAC inhibitors and bromodomain inhibitors as potential inducers of the MET program.^520^ However, MET program plays a key role in metastatic colony formation.^48^ Thus, the opportune moment of MET induction treatment is undoubtedly the most important. Nonetheless, such an “EMT reverse strategy” still holds a very attractive perspective for clinical application.

## 9 CONCLUSIONS AND FUTURE PERSPECTIVES

EMT underlies various physiological and pathological processes, including embryonic development, wound healing, tissue fibrosis, and cancer progression. This event is largely driven by a class of EMT‐TFs, whose functions are context dependent, therefore EMT status cannot be evaluated based on a single or a very few marks.^1^ To this end, EMT status is encouraged to be assessed by certain “EMT signatures” containing dozens of related genes including epithelial marks, mesenchymal marks, EMT‐TFs, and upstream or downstream factors.^521^, ^522^, ^523^ In addition to the reliability, EMT signatures can also reveal the hybrid EMT state (also known as partial EMT, incomplete EMT, or intermediate EMT), which is a common event, whereas absolute epithelial or mesenchymal state are rarely observed during cancer progression. Moreover, it will be more convincing to evaluate EMT status using gene signature in conjunction with morphological changes, such as the presence of a spindle‐like shape or the formation of membrane protrusions.

Classical EMT‐TFs including Snail, Twist, and ZEB are potent inducers of EMT, which has been extensively investigated. Nevertheless, as a rapid evolving field, novel EMT regulators are gradually identified, notably noncoding RNAs (e.g., microRNAs and lncRNAs).^524^, ^525^ These EMT‐regulating microRNAs include miR‑1, miR‐22, miR‑29, miR‑30, miR‑34, miR‐125, miR‐130, miR‐182, miR‑192, miR‑200, miR‑203, miR‐205, miR‐216, miR‐217, miR‐331, miR‑365, miR‐506, miR‐517, miR‐1199, and many others.^105^, ^453^, ^475^, ^526^, ^527^, ^528^, ^529^, ^530^, ^531^, ^532^, ^533^, ^534^, ^535^, ^536^, ^537^, ^538^, ^539^, ^540^ However, most these microRNAs regulate EMT through targeting classical EMT‐TFs, and lncRNAs affect EMT via serving as molecular sponge targeting those microRNAs. For example, miR‐205 has been shown to prevent EMT via repressing of ZEB in the metastasis of breast cancer.^105^ Meanwhile, lncRNA PNUTS competitively binds with miR‐205, leading to the upregulation of ZEB and consequent EMT during tumor progression.^541^ Therefore, regulation of EMT by ncRNAs is probably better to be a part of classical EMT‐TF pathways, instead of a novel mechanism. Nevertheless, several microRNAs have been found to regulate EMT independent of EMT‐TFs. For instance, miR‐9 directly targets E‐cadherin mRNA for degradation, leading to a EMT phenotype and consequent tumor metastasis in breast cancer.^106^ Besides, miR‐22 can also directly target E‐cadherin, enhancing the invasiveness of prostate cancer cells.^542^ Moreover, lncRNA NEAT1 facilitates the formation of FOXN3–NEAT1–SIN3A complex, which directly represses the transcription of GATA3 and ZO‐1, leading to EMT.^543^ These observations suggest a direct regulatory mechanism of EMT by ncRNAs, but more evidence is still required for ascertain it. Recently, a large number of transcription factors and miRNAs were identified as potential novel regulators of EMT, but as well, the possibility that these novel regulators act through classical EMT‐TFs was not precluded.^544^ More intriguingly, E‐cadherin has been shown to be degraded in response to oxidative stress. TNF‐α‐induced oxidative stress can modulate the phosphorylation of E‐cadherin/β‐catenin complex, thus facilitating the degradation of E‐cadherin without altering the mRNA level of E‐cadherin.^408^ Administration of NAC restores the E‐cadherin protein expression.^545^ Furthermore, E‐cadherin has been shown to be degraded through autophagy.^546^, ^547^ Hence, the regulation of EMT at the protein level might be at least partially different from the well‐known epigenetic mechanisms induced by EMT‐TFs, which deserves further investigation.

In terms of cancer therapy, EMT is an attractive target due to the fact that EMT is closely linked with the hallmarks of cancer such as metastasis, metabolism, stemness, drug sensitivity and immune microenvironment. Generally, EMT gives rise to a more aggressive phenotype in a majority of cancer, thus inhibiting EMT can be a potential therapeutic strategy. However, the reversed process of EMT, namely MET, is also required for distant colonization of metastatic cancer cells. Moreover, incomplete inhibition of EMT program might result in the hybrid EMT state, which is charactered with high plasticity, stemness, invasiveness, and drug resistant property, leading to a more refractory malignancy. In contrast, EMT sometimes provides opportunity for cancer treatment, as evidenced by the fact that tumor cells in complete mesenchymal state are highly susceptible to ferroptosis. This evidence suggests that strategies targeting EMT hold therapeutic potential but still need in‐depth understanding of the mechanisms of EMT.

## CONFLICT OF INTEREST

Canhua Huang is an editorial board member of MedComm. Author Canhua Huang was not involved in the journal's review of, or decisions related to, this manuscript. The other authors have no conflicts of interest to declare.

## ETHICS STATEMENT

Not applicable.

## AUTHOR CONTRIBUTIONS

C. H., L. L., and C. Z. conceived the manuscript; C. H., Z. H., and Z. Z. wrote and edited the manuscript. All authors read and approved the final manuscript. Z. H. and Z. Z. contributed equally to this work.

## Data Availability

Not applicable.

## References

[1] Yang J , Antin P , Berx G , et al. Guidelines and definitions for research on epithelial‐mesenchymal transition. Nat Rev Mol Cell Biol. 2020;21(6):341‐352. 32300252 10.1038/s41580-020-0237-9 PMC7250738

[2] Dongre A , Weinberg RA . New insights into the mechanisms of epithelial‐mesenchymal transition and implications for cancer. Nat Rev Mol Cell Biol. 2019;20(2):69‐84. 30459476 10.1038/s41580-018-0080-4

[3] Gonzalez DM , Medici D . Signaling mechanisms of the epithelial‐mesenchymal transition. Sci Signal. 2014;7(344):re8. 25249658 10.1126/scisignal.2005189 PMC4372086

[4] Caramel J , Papadogeorgakis E , Hill L , et al. A switch in the expression of embryonic EMT‐inducers drives the development of malignant melanoma. Cancer Cell. 2013;24(4):466‐480. 24075834 10.1016/j.ccr.2013.08.018

[5] Fazilaty H , Rago L , Kass Youssef K , et al. A gene regulatory network to control EMT programs in development and disease. Nat Commun. 2019;10(1):5115. 31712603 10.1038/s41467-019-13091-8 PMC6848104

[6] Owusu‐Akyaw A , Krishnamoorthy K , Goldsmith LT , Morelli SS . The role of mesenchymal‐epithelial transition in endometrial function. Hum Reprod Update. 2019;25(1):114‐133. 30407544 10.1093/humupd/dmy035

[7] Cheng F , Shen Y , Mohanasundaram P , et al. Vimentin coordinates fibroblast proliferation and keratinocyte differentiation in wound healing via TGF‐beta‐Slug signaling. Proc Natl Acad Sci USA. 2016;113(30):E4320‐E4327. 27466403 10.1073/pnas.1519197113 PMC4968728

[8] Kalluri R , Neilson EG . Epithelial‐mesenchymal transition and its implications for fibrosis. J Clin Invest. 2003;112(12):1776‐1784. 14679171 10.1172/JCI20530 PMC297008

[9] Yang J , Mani SA , Donaher JL , et al. Twist, a master regulator of morphogenesis, plays an essential role in tumor metastasis. Cell. 2004;117(7):927‐939. 15210113 10.1016/j.cell.2004.06.006

[10] Yilmaz M , Christofori G . EMT, the cytoskeleton, and cancer cell invasion. Cancer Metastasis Rev. 2009;28(1‐2):15‐33. 19169796 10.1007/s10555-008-9169-0

[11] Li A , Morton JP , Ma Y , et al. Fascin is regulated by slug, promotes progression of pancreatic cancer in mice, and is associated with patient outcomes. Gastroenterology. 2014;146(5):1386‐1396. e1‐17. 24462734 10.1053/j.gastro.2014.01.046 PMC4000441

[12] Shankar J , Messenberg A , Chan J , Underhill TM , Foster LJ , Nabi IR . Pseudopodial actin dynamics control epithelial‐mesenchymal transition in metastatic cancer cells. Cancer Res. 2010;70(9):3780‐3790. 20388789 10.1158/0008-5472.CAN-09-4439

[13] Radisky DC , Levy DD , Littlepage LE , et al. Rac1b and reactive oxygen species mediate MMP‐3‐induced EMT and genomic instability. Nature. 2005;436(7047):123‐127. 16001073 10.1038/nature03688 PMC2784913

[14] Stallings‐Mann ML , Waldmann J , Zhang Y , et al. Matrix metalloproteinase induction of Rac1b, a key effector of lung cancer progression. Sci Transl Med. 2012;4(142):142ra95. 10.1126/scitranslmed.3004062 PMC3733503 22786680

[15] Horejs CM , St‐Pierre JP , Ojala JRM , et al. Preventing tissue fibrosis by local biomaterials interfacing of specific cryptic extracellular matrix information. Nat Commun. 2017;8:15509. 28593951 10.1038/ncomms15509 PMC5472175

[16] Zhang Q , Liu S , Parajuli KR , et al. Interleukin‐17 promotes prostate cancer via MMP7‐induced epithelial‐to‐mesenchymal transition. Oncogene. 2017;36(5):687‐699. 27375020 10.1038/onc.2016.240 PMC5213194

[17] Krebs AM , Mitschke J , Lasierra Losada M , et al. The EMT‐activator Zeb1 is a key factor for cell plasticity and promotes metastasis in pancreatic cancer. Nat Cell Biol. 2017;19(5):518‐529. 28414315 10.1038/ncb3513

[18] Ye X , Tam WL , Shibue T , et al. Distinct EMT programs control normal mammary stem cells and tumour‐initiating cells. Nature. 2015;525(7568):256‐260. 26331542 10.1038/nature14897 PMC4764075

[19] Goossens S , Vandamme N , Van Vlierberghe P , Berx G . EMT transcription factors in cancer development re‐evaluated: beyond EMT and MET. Biochim Biophys Acta Rev Cancer. 2017;1868(2):584‐591. 28669750 10.1016/j.bbcan.2017.06.006

[20] Lamouille S , Xu J , Derynck R . Molecular mechanisms of epithelial‐mesenchymal transition. Nat Rev Mol Cell Biol. 2014;15(3):178‐196. 24556840 10.1038/nrm3758 PMC4240281

[21] Evdokimova V , Tognon C , Ng T , et al. Translational activation of snail1 and other developmentally regulated transcription factors by YB‐1 promotes an epithelial‐mesenchymal transition. Cancer Cell. 2009;15(5):402‐415. 19411069 10.1016/j.ccr.2009.03.017

[22] Meng J , Chen S , Han JX , et al. Twist1 regulates vimentin through Cul2 circular RNA to promote EMT in hepatocellular carcinoma. Cancer Res. 2018;78(15):4150‐4162. 29844124 10.1158/0008-5472.CAN-17-3009

[23] Weiss MB , Abel EV , Mayberry MM , Basile KJ , Berger AC , Aplin AE . TWIST1 is an ERK1/2 effector that promotes invasion and regulates MMP‐1 expression in human melanoma cells. Cancer Res. 2012;72(24):6382‐6692. 23222305 10.1158/0008-5472.CAN-12-1033 PMC3531871

[24] Su J , Morgani SM , David CJ , et al. TGF‐beta orchestrates fibrogenic and developmental EMTs via the RAS effector RREB1. Nature. 2020;577(7791):566‐571. 31915377 10.1038/s41586-019-1897-5 PMC7450666

[25] Scheel C , Eaton EN , Li SH , et al. Paracrine and autocrine signals induce and maintain mesenchymal and stem cell states in the breast. Cell. 2011;145(6):926‐940. 21663795 10.1016/j.cell.2011.04.029 PMC3930331

[26] Oh SH , Swiderska‐Syn M , Jewell ML , Premont RT , Diehl AM . Liver regeneration requires Yap1‐TGFbeta‐dependent epithelial‐mesenchymal transition in hepatocytes. J Hepatol. 2018;69(2):359‐367. 29758331 10.1016/j.jhep.2018.05.008 PMC6349217

[27] Gujral TS , Chan M , Peshkin L , Sorger PK , Kirschner MW , MacBeath G . A noncanonical Frizzled2 pathway regulates epithelial‐mesenchymal transition and metastasis. Cell. 2014;159(4):844‐856. 25417160 10.1016/j.cell.2014.10.032 PMC4243058

[28] Murillo‐Garzon V , Gorrono‐Etxebarria I , Akerfelt M , et al. Frizzled‐8 integrates Wnt‐11 and transforming growth factor‐beta signaling in prostate cancer. Nat Commun. 2018;9(1): 1747. 29717114 10.1038/s41467-018-04042-w PMC5931552

[29] Omenetti A , Porrello A , Jung Y , et al. Hedgehog signaling regulates epithelial‐mesenchymal transition during biliary fibrosis in rodents and humans. J Clin Invest. 2008;118(10):3331‐3342. 18802480 10.1172/JCI35875 PMC2542850

[30] Huang Z , Zhou JK , Wang K , et al. PDLIM1 inhibits tumor metastasis through activating hippo signaling in hepatocellular carcinoma. Hepatology. 2020;71(5):1643‐1659. 31509262 10.1002/hep.30930

[31] Aharonov A , Shakked A , Umansky KB , et al. ERBB2 drives YAP activation and EMT‐like processes during cardiac regeneration. Nat Cell Biol. 2020;22(11):1346‐1356. 33046882 10.1038/s41556-020-00588-4

[32] Mittal V . Epithelial mesenchymal transition in tumor metastasis. Annu Rev Pathol. 2018;13:395‐412. 29414248 10.1146/annurev-pathol-020117-043854

[33] Nieto MA , Huang RY , Jackson RA , Thiery JP . Emt: 2016. Cell. 2016;166(1):21‐45. 27368099 10.1016/j.cell.2016.06.028

[34] Lambert AW , Weinberg RA . Linking EMT programmes to normal and neoplastic epithelial stem cells. Nat Rev Cancer. 2021;21(5):325‐338. 33547455 10.1038/s41568-021-00332-6

[35] Shibue T , Weinberg RA . EMT, CSCs, and drug resistance: the mechanistic link and clinical implications. Nat Rev Clin Oncol. 2017;14(10):611‐629. 28397828 10.1038/nrclinonc.2017.44 PMC5720366

[36] Brabletz T , Kalluri R , Nieto MA , Weinberg RA . EMT in cancer. Nat Rev Cancer. 2018;18(2):128‐134. 29326430 10.1038/nrc.2017.118

[37] Fiori ME , Di Franco S , Villanova L , Bianca P , Stassi G , De Maria R . Cancer‐associated fibroblasts as abettors of tumor progression at the crossroads of EMT and therapy resistance. Mol Cancer. 2019;18(1):70. 30927908 10.1186/s12943-019-0994-2 PMC6441236

[38] Shaul YD , Freinkman E , Comb WC , et al. Dihydropyrimidine accumulation is required for the epithelial‐mesenchymal transition. Cell. 2014;158(5):1094‐1109. 25171410 10.1016/j.cell.2014.07.032 PMC4250222

[39] Wu X , Li X , Fu Q , et al. AKR1B1 promotes basal‐like breast cancer progression by a positive feedback loop that activates the EMT program. J Exp Med. 2017;214(4):1065‐1079. 28270406 10.1084/jem.20160903 PMC5379972

[40] Brown RL , Reinke LM , Damerow MS , et al. CD44 splice isoform switching in human and mouse epithelium is essential for epithelial‐mesenchymal transition and breast cancer progression. J Clin Invest. 2011;121(3):1064‐1074. 21393860 10.1172/JCI44540 PMC3049398

[41] Yu M , Bardia A , Wittner BS , et al. Circulating breast tumor cells exhibit dynamic changes in epithelial and mesenchymal composition. Science. 2013;339(6119):580‐584. 23372014 10.1126/science.1228522 PMC3760262

[42] Mani SA , Guo W , Liao MJ , et al. The epithelial‐mesenchymal transition generates cells with properties of stem cells. Cell. 2008;133(4):704‐715. 18485877 10.1016/j.cell.2008.03.027 PMC2728032

[43] Karvelsson ST , Sigurdsson A , Seip K , et al. EMT‐derived alterations in glutamine metabolism sensitize mesenchymal breast cells to mTOR inhibition. Mol Cancer Res. 2021;19(9):1546‐1558. 34088869 10.1158/1541-7786.MCR-20-0962

[44] Schwab A , Siddiqui A , Vazakidou ME , et al. Polyol pathway links glucose metabolism to the aggressiveness of cancer cells. Cancer Res. 2018;78(7):1604‐1618. 29343522 10.1158/0008-5472.CAN-17-2834

[45] Suarez‐Carmona M , Lesage J , Cataldo D , Gilles C . EMT and inflammation: inseparable actors of cancer progression. Mol Oncol. 2017;11(7):805‐823. 28599100 10.1002/1878-0261.12095 PMC5496491

[46] Brabletz S , Schuhwerk H , Brabletz T , Stemmler MP . Dynamic EMT: a multi‐tool for tumor progression. EMBO J. 2021;40(18):e108647. 34459003 10.15252/embj.2021108647 PMC8441439

[47] Jolly MK , Mani SA , Levine H . Hybrid epithelial/mesenchymal phenotype(s): the ‘fittest’ for metastasis?. Biochim Biophys Acta Rev Cancer. 2018;1870(2):151‐157. 29997040 10.1016/j.bbcan.2018.07.001

[48] Bakir B , Chiarella AM , Pitarresi JR , Rustgi AK . EMT, MET, plasticity, and tumor metastasis. Trends Cell Biol. 2020;30(10):764‐776. 32800658 10.1016/j.tcb.2020.07.003 PMC7647095

[49] Aiello NM , Kang Y . Context‐dependent EMT programs in cancer metastasis. J Exp Med. 2019;216(5):1016‐1026. 30975895 10.1084/jem.20181827 PMC6504222

[50] Lee J , You JH , Kim MS , Roh JL . Epigenetic reprogramming of epithelial‐mesenchymal transition promotes ferroptosis of head and neck cancer. Redox Biol. 2020;37:101697. 32896720 10.1016/j.redox.2020.101697 PMC7484553

[51] Lin CC , Yang WH , Lin YT , et al. DDR2 upregulation confers ferroptosis susceptibility of recurrent breast tumors through the Hippo pathway. Oncogene. 2021;40(11):2018‐2034. 33603168 10.1038/s41388-021-01676-x PMC7988308

[52] Zhang N , Ng AS , Cai S , Li Q , Yang L , Kerr D . Novel therapeutic strategies: targeting epithelial‐mesenchymal transition in colorectal cancer. Lancet Oncol. 2021;22(8):e358‐e368. 34339656 10.1016/S1470-2045(21)00343-0

[53] Ramesh V , Brabletz T , Ceppi P . Targeting EMT in cancer with repurposed metabolic inhibitors. Trends Cancer. 2020;6(11):942‐950. 32680650 10.1016/j.trecan.2020.06.005

[54] Davis FM , Stewart TA , Thompson EW , Monteith GR . Targeting EMT in cancer: opportunities for pharmacological intervention. Trends Pharmacol Sci. 2014;35(9):479‐488. 25042456 10.1016/j.tips.2014.06.006

[55] Erin N , Grahovac J , Brozovic A , Efferth T . Tumor microenvironment and epithelial mesenchymal transition as targets to overcome tumor multidrug resistance. Drug Resist Updat. 2020;53:100715. 32679188 10.1016/j.drup.2020.100715

[56] Hay ED . The fine structure of blastema cells and differentiating cartilage cells in regenerating limbs of Amblystoma larvae. J Biophys Biochem Cytol. 1958;4(5):583‐591. 13587554 10.1083/jcb.4.5.583 PMC2224557

[57] Lachat C , Peixoto P , Hervouet E . Epithelial to mesenchymal transition history: from embryonic development to cancers. Biomolecules. 2021;11(6):782. 34067395 10.3390/biom11060782 PMC8224685

[58] Hay ED , Fischman DA . Origin of the blastema in regenerating limbs of the newt Triturus viridescens. An autoradiographic study using tritiated thymidine to follow cell proliferation and migration. Dev Biol. 1961;3:26‐59. 13712434 10.1016/0012-1606(61)90009-4

[59] Trelstad RL , Revel JP , Hay ED . Tight junctions between cells in the early chick embryo as visualized with the electron microscopy. J Cell Biol. 1966;31(1):C6‐10. 5971977 10.1083/jcb.31.1.c6 PMC2107036

[60] Trelstad RL , Hay ED , Revel JD . Cell contact during early morphogenesis in the chick embryo. Dev Biol. 1967;16(1):78‐106. 6035571 10.1016/0012-1606(67)90018-8

[61] Markwald RR , Fitzharris TP , Manasek FJ . Structural development of endocardial cushions. Am J Anat. 1977;148(1):85‐119. 842477 10.1002/aja.1001480108

[62] Newgreen DF , Ritterman M , Peters EA . Morphology and behaviour of neural crest cells of chick embryo in vitro. Cell Tissue Res. 1979;203(1):115‐140. 509508 10.1007/BF00234333

[63] Dyche WJ . A comparative study of the differentiation and involution of the Mullerian duct and Wolffian duct in the male and female fetal mouse. J Morphol. 1979;162(2):175‐209. 537099 10.1002/jmor.1051620203

[64] Kedinger M , Simon PM , Grenier JF , Haffen K . Role of epithelial–mesenchymal interactions in the ontogenesis of intestinal brush‐border enzymes. Dev Biol. 1981;86(2):339‐347. 6793427 10.1016/0012-1606(81)90191-3

[65] Bluemink JG , Van Maurik P , Lawson KA . Intimate cell contacts at the epithelial/mesenchymal interface in embryonic mouse lung. J Ultrastruct Res. 1976;55(2):257‐270. 1271517 10.1016/s0022-5320(76)80071-8

[66] Greenburg G , Hay ED . Epithelia suspended in collagen gels can lose polarity and express characteristics of migrating mesenchymal cells. J Cell Biol. 1982;95(1):333‐339. 7142291 10.1083/jcb.95.1.333 PMC2112361

[67] Hay ED , Zuk A . Transformations between epithelium and mesenchyme: normal, pathological, and experimentally induced. Am J Kidney Dis. 1995;26(4):678‐690. 7573028 10.1016/0272-6386(95)90610-x

[68] Stoker M , Perryman M . An epithelial scatter factor released by embryo fibroblasts. J Cell Sci. 1985;77:209‐223. 3841349 10.1242/jcs.77.1.209

[69] Stoker M , Gherardi E , Perryman M , Gray J . Scatter factor is a fibroblast‐derived modulator of epithelial cell mobility. Nature. 1987;327(6119):239‐242. 2952888 10.1038/327239a0

[70] Valles AM , Boyer B , Badet J , Tucker GC , Barritault D , Thiery JP . Acidic fibroblast growth factor is a modulator of epithelial plasticity in a rat bladder carcinoma cell line. Proc Natl Acad Sci USA. 1990;87(3):1124‐1128. 2153969 10.1073/pnas.87.3.1124 PMC53423

[71] Gavrilovic J , Moens G , Thiery JP , Jouanneau J . Expression of transfected transforming growth factor alpha induces a motile fibroblast‐like phenotype with extracellular matrix‐degrading potential in a rat bladder carcinoma cell line. Cell Regul. 1990;1(13):1003‐1014. 2134746 10.1091/mbc.1.13.1003 PMC361698

[72] Pagan R , Martin I , Llobera M , Vilaro S . Epithelial‐mesenchymal transition of cultured rat neonatal hepatocytes is differentially regulated in response to epidermal growth factor and dimethyl sulfoxide. Hepatology. 1997;25(3):598‐606. 9049205 10.1002/hep.510250318

[73] Akhurst RJ , Lehnert SA , Faissner A , Duffie E . TGF beta in murine morphogenetic processes: the early embryo and cardiogenesis. Development. 1990;108(4):645‐656. 1696875 10.1242/dev.108.4.645

[74] Yang EY , Moses HL . Transforming growth factor beta 1‐induced changes in cell migration, proliferation, and angiogenesis in the chicken chorioallantoic membrane. J Cell Biol. 1990;111(2):731‐741. 1696268 10.1083/jcb.111.2.731 PMC2116177

[75] Miettinen PJ , Ebner R , Lopez AR , Derynck R . TGF‐beta induced transdifferentiation of mammary epithelial cells to mesenchymal cells: involvement of type I receptors. J Cell Biol. 1994;127(6 Pt 2):2021‐2036. 7806579 10.1083/jcb.127.6.2021 PMC2120317

[76] Heldin CH , Miyazono K , ten Dijke P . TGF‐beta signalling from cell membrane to nucleus through SMAD proteins. Nature. 1997;390(6659):465‐471. 9393997 10.1038/37284

[77] Piek E , Moustakas A , Kurisaki A , Heldin CH , ten Dijke P . TGF‐(beta) type I receptor/ALK‐5 and Smad proteins mediate epithelial to mesenchymal transdifferentiation in NMuMG breast epithelial cells. J Cell Sci. 1999;112(Pt 24):4557‐4568. 10574705 10.1242/jcs.112.24.4557

[78] Oft M , Heider KH , Beug H . TGFbeta signaling is necessary for carcinoma cell invasiveness and metastasis. Curr Biol. 1998;8(23):1243‐1252. 9822576 10.1016/s0960-9822(07)00533-7

[79] Smith DE , Franco del Amo F , Gridley T . Isolation of Sna, a mouse gene homologous to the Drosophila genes snail and escargot: its expression pattern suggests multiple roles during postimplantation development. Development. 1992;116(4):1033‐1039. 1295727 10.1242/dev.116.4.1033

[80] Nieto MA , Sargent MG , Wilkinson DG , Cooke J . Control of cell behavior during vertebrate development by Slug, a zinc finger gene. Science. 1994;264(5160):835‐839. 7513443 10.1126/science.7513443

[81] Bloch‐Zupan A , Hunter N , Manthey A , Gibbins J . R‐twist gene expression during rat palatogenesis. Int J Dev Biol. 2001;45(2):397‐404. 11330859

[82] Comijn J , Berx G , Vermassen P , et al. The two‐handed E box binding zinc finger protein SIP1 downregulates E‐cadherin and induces invasion. Mol Cell. 2001;7(6):1267‐1278. 11430829 10.1016/s1097-2765(01)00260-x

[83] Cano A , Perez‐Moreno MA , Rodrigo I , et al. The transcription factor snail controls epithelial‐mesenchymal transitions by repressing E‐cadherin expression. Nat Cell Biol. 2000;2(2):76‐83. 10655586 10.1038/35000025

[84] Batlle E , Sancho E , Franci C , et al. The transcription factor snail is a repressor of E‐cadherin gene expression in epithelial tumour cells. Nat Cell Biol. 2000;2(2):84‐89. 10655587 10.1038/35000034

[85] Hajra KM , Chen DY , Fearon ER . The SLUG zinc‐finger protein represses E‐cadherin in breast cancer. Cancer Res. 2002;62(6):1613‐1618. 11912130

[86] Bolos V , Peinado H , Perez‐Moreno MA , Fraga MF , Esteller M , Cano A . The transcription factor Slug represses E‐cadherin expression and induces epithelial to mesenchymal transitions: a comparison with Snail and E47 repressors. J Cell Sci. 2003;116(Pt 3):499‐511. 12508111 10.1242/jcs.00224

[87] Morita T , Mayanagi T , Sobue K . Dual roles of myocardin‐related transcription factors in epithelial mesenchymal transition via slug induction and actin remodeling. J Cell Biol. 2007;179(5):1027‐1042. 18056415 10.1083/jcb.200708174 PMC2099179

[88] Zhang K , Chen D , Jiao X , et al. Slug enhances invasion ability of pancreatic cancer cells through upregulation of matrix metalloproteinase‐9 and actin cytoskeleton remodeling. Lab Invest. 2011;91(3):426‐438. 21283078 10.1038/labinvest.2010.201 PMC3125102

[89] Park SH , Cheung LW , Wong AS , Leung PC . Estrogen regulates Snail and Slug in the down‐regulation of E‐cadherin and induces metastatic potential of ovarian cancer cells through estrogen receptor alpha. Mol Endocrinol. 2008;22(9):2085‐2098. 18550773 10.1210/me.2007-0512 PMC5419456

[90] Miyoshi A , Kitajima Y , Sumi K , et al. Snail and SIP1 increase cancer invasion by upregulating MMP family in hepatocellular carcinoma cells. Br J Cancer. 2004;90(6):1265‐1273. 15026811 10.1038/sj.bjc.6601685 PMC2409652

[91] Miyoshi A , Kitajima Y , Kido S , et al. Snail accelerates cancer invasion by upregulating MMP expression and is associated with poor prognosis of hepatocellular carcinoma. Br J Cancer. 2005;92(2):252‐258. 15668718 10.1038/sj.bjc.6602266 PMC2361838

[92] Blanco MJ , Moreno‐Bueno G , Sarrio D , et al. Correlation of Snail expression with histological grade and lymph node status in breast carcinomas. Oncogene. 2002;21(20):3241‐3246. 12082640 10.1038/sj.onc.1205416

[93] Mani SA , Yang J , Brooks M , et al. Mesenchyme Forkhead 1 (FOXC2) plays a key role in metastasis and is associated with aggressive basal‐like breast cancers. Proc Natl Acad Sci USA. 2007;104(24):10069‐10074. 17537911 10.1073/pnas.0703900104 PMC1891217

[94] Belguise K , Guo S , Sonenshein GE . Activation of FOXO3a by the green tea polyphenol epigallocatechin‐3‐gallate induces estrogen receptor alpha expression reversing invasive phenotype of breast cancer cells. Cancer Res. 2007;67(12):5763‐5770. 17575143 10.1158/0008-5472.CAN-06-4327

[95] Nilsson J , Helou K , Kovacs A , et al. Nuclear Janus‐activated kinase 2/nuclear factor 1‐C2 suppresses tumorigenesis and epithelial‐to‐mesenchymal transition by repressing Forkhead box F1. Cancer Res. 2010;70(5):2020‐2029. 20145151 10.1158/0008-5472.CAN-09-1677

[96] Song Y , Washington MK , Crawford HC . Loss of FOXA1/2 is essential for the epithelial‐to‐mesenchymal transition in pancreatic cancer. Cancer Res. 2010;70(5):2115‐2125. 20160041 10.1158/0008-5472.CAN-09-2979 PMC2831111

[97] Qiao Y , Jiang X , Lee ST , Karuturi RK , Hooi SC , Yu Q . FOXQ1 regulates epithelial‐mesenchymal transition in human cancers. Cancer Res. 2011;71(8):3076‐3086. 21346143 10.1158/0008-5472.CAN-10-2787

[98] Campbell K , Whissell G , Franch‐Marro X , Batlle E , Casanova J . Specific GATA factors act as conserved inducers of an endodermal‐EMT. Dev Cell. 2011;21(6):1051‐1061. 22172671 10.1016/j.devcel.2011.10.005

[99] Ocana OH , Corcoles R , Fabra A , et al. Metastatic colonization requires the repression of the epithelial‐mesenchymal transition inducer Prrx1. Cancer Cell. 2012;22(6):709‐724. 23201163 10.1016/j.ccr.2012.10.012

[100] Takahashi Y , Sawada G , Kurashige J , et al. Paired related homoeobox 1, a new EMT inducer, is involved in metastasis and poor prognosis in colorectal cancer. Br J Cancer. 2013;109(2):307‐311. 23807160 10.1038/bjc.2013.339 PMC3721401

[101] Vervoort SJ , Lourenco AR , van Boxtel R , Coffer PJ . SOX4 mediates TGF‐beta‐induced expression of mesenchymal markers during mammary cell epithelial to mesenchymal transition. PLoS One. 2013;8(1):e53238. 23301048 10.1371/journal.pone.0053238 PMC3536747

[102] Ma Y , Shepherd J , Zhao D , et al. SOX9 is essential for triple‐negative breast cancer cell survival and metastasis. Mol Cancer Res. 2020;18(12):1825‐1838. 32661114 10.1158/1541-7786.MCR-19-0311 PMC7718423

[103] Yanagisawa M , Huveldt D , Kreinest P , et al. A p120 catenin isoform switch affects Rho activity, induces tumor cell invasion, and predicts metastatic disease. J Biol Chem. 2008;283(26):18344‐18354. 18407999 10.1074/jbc.M801192200 PMC2440599

[104] Shapiro IM , Cheng AW , Flytzanis NC , et al. An EMT‐driven alternative splicing program occurs in human breast cancer and modulates cellular phenotype. PLoS Genet. 2011;7(8):e1002218. 21876675 10.1371/journal.pgen.1002218 PMC3158048

[105] Gregory PA , Bert AG , Paterson EL , et al. The miR‐200 family and miR‐205 regulate epithelial to mesenchymal transition by targeting ZEB1 and SIP1. Nat Cell Biol. 2008;10(5):593‐601. 18376396 10.1038/ncb1722

[106] Ma L , Young J , Prabhala H , et al. miR‐9, a MYC/MYCN‐activated microRNA, regulates E‐cadherin and cancer metastasis. Nat Cell Biol. 2010;12(3):247‐256. 20173740 10.1038/ncb2024 PMC2845545

[107] Conn SJ , Pillman KA , Toubia J , et al. The RNA binding protein quaking regulates formation of circRNAs. Cell. 2015;160(6):1125‐1134. 25768908 10.1016/j.cell.2015.02.014

[108] Pastushenko I , Blanpain C . EMT transition states during tumor progression and metastasis. Trends Cell Biol. 2019;29(3):212‐226. 30594349 10.1016/j.tcb.2018.12.001

[109] Jordan NV , Johnson GL , Abell AN . Tracking the intermediate stages of epithelial‐mesenchymal transition in epithelial stem cells and cancer. Cell Cycle. 2011;10(17):2865‐2873. 21862874 10.4161/cc.10.17.17188 PMC3218599

[110] Huang RY , Wong MK , Tan TZ , et al. An EMT spectrum defines an anoikis‐resistant and spheroidogenic intermediate mesenchymal state that is sensitive to e‐cadherin restoration by a src‐kinase inhibitor, saracatinib (AZD0530). Cell Death Dis. 2013;4:e915. 24201814 10.1038/cddis.2013.442 PMC3847320

[111] Pastushenko I , Brisebarre A , Sifrim A , et al. Identification of the tumour transition states occurring during EMT. Nature. 2018;556(7702):463‐468. 29670281 10.1038/s41586-018-0040-3

[112] Luond F , Sugiyama N , Bill R , et al. Distinct contributions of partial and full EMT to breast cancer malignancy. Dev Cell. 2021;56(23):3203‐3221. e11. 34847378 10.1016/j.devcel.2021.11.006

[113] Lu W , Kang Y . Epithelial‐mesenchymal plasticity in cancer progression and metastasis. Dev Cell. 2019;49(3):361‐374. 31063755 10.1016/j.devcel.2019.04.010 PMC6506183

[114] Pastushenko I , Mauri F , Song Y , et al. Fat1 deletion promotes hybrid EMT state, tumour stemness and metastasis. Nature. 2021;589(7842):448‐455. 33328637 10.1038/s41586-020-03046-1 PMC7612440

[115] Fischer KR , Durrans A , Lee S , et al. Epithelial‐to‐mesenchymal transition is not required for lung metastasis but contributes to chemoresistance. Nature. 2015;527(7579):472‐476. 26560033 10.1038/nature15748 PMC4662610

[116] Zheng X , Carstens JL , Kim J , et al. Epithelial‐to‐mesenchymal transition is dispensable for metastasis but induces chemoresistance in pancreatic cancer. Nature. 2015;527(7579):525‐530. 26560028 10.1038/nature16064 PMC4849281

[117] Beerling E , Seinstra D , de Wit E , et al. Plasticity between epithelial and mesenchymal states unlinks EMT from metastasis‐enhancing stem cell capacity. Cell Rep. 2016;14(10):2281‐2288. 26947068 10.1016/j.celrep.2016.02.034 PMC4802222

[118] Padmanaban V , Krol I , Suhail Y , et al. E‐cadherin is required for metastasis in multiple models of breast cancer. Nature. 2019;573(7774):439‐444. 31485072 10.1038/s41586-019-1526-3 PMC7365572

[119] Mendonsa AM , Na TY , Gumbiner BM . E‐cadherin in contact inhibition and cancer. Oncogene. 2018;37(35):4769‐4780. 29780167 10.1038/s41388-018-0304-2 PMC6119098

[120] Li CF , Chen JY , Ho YH , et al. Snail‐induced claudin‐11 prompts collective migration for tumour progression. Nat Cell Biol. 2019;21(2):251‐262. 30664792 10.1038/s41556-018-0268-z

[121] Kam CY , Dubash AD , Magistrati E , et al. Desmoplakin maintains gap junctions by inhibiting Ras/MAPK and lysosomal degradation of connexin‐43. J Cell Biol. 2018;217(9):3219‐3235. 29959233 10.1083/jcb.201710161 PMC6123000

[122] Hall A . Rho GTPases and the actin cytoskeleton. Science. 1998;279(5350):509‐514. 9438836 10.1126/science.279.5350.509

[123] Garcia MA , Nelson WJ , Chavez N . Cell‐Cell junctions organize structural and signaling networks. Cold Spring Harb Perspect Biol. 2018;10(4). 10.1101/cshperspect.a029181 PMC5773398 28600395

[124] Moujaber O , Stochaj U . The cytoskeleton as regulator of cell signaling pathways. Trends Biochem Sci. 2020;45(2):96‐107. 31812462 10.1016/j.tibs.2019.11.003

[125] Menke A , Giehl K . Regulation of adherens junctions by Rho GTPases and p120‐catenin. Arch Biochem Biophys. 2012;524(1):48‐55. 22583808 10.1016/j.abb.2012.04.019

[126] Ciriello G , Gatza ML , Beck AH , et al. Comprehensive molecular portraits of invasive lobular breast cancer. Cell. 2015;163(2):506‐519. 26451490 10.1016/j.cell.2015.09.033 PMC4603750

[127] Guilford P , Hopkins J , Harraway J , et al. E‐cadherin germline mutations in familial gastric cancer. Nature. 1998;392(6674):402‐405. 9537325 10.1038/32918

[128] Huels DJ , Ridgway RA , Radulescu S , et al. E‐cadherin can limit the transforming properties of activating beta‐catenin mutations. EMBO J. 2015;34(18):2321‐2333. 26240067 10.15252/embj.201591739 PMC4570519

[129] Ceteci F , Ceteci S , Karreman C , et al. Disruption of tumor cell adhesion promotes angiogenic switch and progression to micrometastasis in RAF‐driven murine lung cancer. Cancer Cell. 2007;12(2):145‐159. 17692806 10.1016/j.ccr.2007.06.014

[130] Li CI , Anderson BO , Daling JR , Moe RE . Trends in incidence rates of invasive lobular and ductal breast carcinoma. JAMA. 2003;289(11):1421‐1424. 12636465 10.1001/jama.289.11.1421

[131] van Roy F . Beyond E‐cadherin: roles of other cadherin superfamily members in cancer. Nat Rev Cancer. 2014;14(2):121‐134. 24442140 10.1038/nrc3647

[132] Tanaka H , Kono E , Tran CP , et al. Monoclonal antibody targeting of N‐cadherin inhibits prostate cancer growth, metastasis and castration resistance. Nat Med. 2010;16(12):1414‐1420. 21057494 10.1038/nm.2236 PMC3088104

[133] Agiostratidou G , Li M , Suyama K , et al. Loss of retinal cadherin facilitates mammary tumor progression and metastasis. Cancer Res. 2009;69(12):5030‐5508. 19491271 10.1158/0008-5472.CAN-08-4007 PMC4382672

[134] Vieira AF , Paredes J . P‐cadherin and the journey to cancer metastasis. Mol Cancer. 2015;14:178. 26438065 10.1186/s12943-015-0448-4 PMC4595126

[135] Imai S , Kobayashi M , Takasaki C , Ishibashi H , Okubo K . High expression of P‐cadherin is significantly associated with poor prognosis in patients with non‐small‐cell lung cancer. Lung Cancer. 2018;118:13‐19. 29571991 10.1016/j.lungcan.2018.01.018

[136] Cheung LW , Mak AS , Cheung AN , Ngan HY , Leung PC , Wong AS . P‐cadherin cooperates with insulin‐like growth factor‐1 receptor to promote metastatic signaling of gonadotropin‐releasing hormone in ovarian cancer via p120 catenin. Oncogene. 2011;30(26):2964‐2974. 21317933 10.1038/onc.2011.7

[137] Van Marck V , Stove C , Van Den Bossche K , et al. P‐cadherin promotes cell‐cell adhesion and counteracts invasion in human melanoma. Cancer Res. 2005;65(19):8774‐8783. 16204047 10.1158/0008-5472.CAN-04-4414

[138] Jacobs K , Van Gele M , Forsyth R , et al. P‐cadherin counteracts myosin II‐B function: implications in melanoma progression. Mol Cancer. 2010;9:255. 20860798 10.1186/1476-4598-9-255 PMC2949802

[139] Kyuno D , Takasawa A , Kikuchi S , Takemasa I , Osanai M , Kojima T . Role of tight junctions in the epithelial‐to‐mesenchymal transition of cancer cells. Biochim Biophys Acta Biomembr. 2021;1863(3):183503. 33189716 10.1016/j.bbamem.2020.183503

[140] Singh AB , Sharma A , Smith JJ , et al. Claudin‐1 up‐regulates the repressor ZEB‐1 to inhibit E‐cadherin expression in colon cancer cells. Gastroenterology. 2011;141(6):2140‐2153. 21878201 10.1053/j.gastro.2011.08.038 PMC3395068

[141] Chang TL , Ito K , Ko TK , et al. Claudin‐1 has tumor suppressive activity and is a direct target of RUNX3 in gastric epithelial cells. Gastroenterology. 2010;138(1):255‐265. e1‐3. 19706291 10.1053/j.gastro.2009.08.044

[142] Tabaries S , McNulty A , Ouellet V , et al. Afadin cooperates with Claudin‐2 to promote breast cancer metastasis. Genes Dev. 2019;33(3‐4):180‐193. 30692208 10.1101/gad.319194.118 PMC6362814

[143] Paquet‐Fifield S , Koh SL , Cheng L , et al. Tight junction protein claudin‐2 promotes self‐renewal of human colorectal cancer stem‐like cells. Cancer Res. 2018;78(11):2925‐2938. 29510994 10.1158/0008-5472.CAN-17-1869

[144] Hichino A , Okamoto M , Taga S , et al. Down‐regulation of Claudin‐2 expression and proliferation by epigenetic inhibitors in human lung adenocarcinoma A549 cells. J Biol Chem. 2017;292(6):2411‐2421. 28057758 10.1074/jbc.M116.762807 PMC5313110

[145] Buchert M , Papin M , Bonnans C , et al. Symplekin promotes tumorigenicity by up‐regulating claudin‐2 expression. Proc Natl Acad Sci USA. 2010;107(6):2628‐2633. 20133805 10.1073/pnas.0903747107 PMC2823888

[146] Lee JH , Kim KS , Kim TJ , et al. Immunohistochemical analysis of claudin expression in pancreatic cystic tumors. Oncol Rep. 2011;25(4):971‐978. 21206985 10.3892/or.2011.1132

[147] Zhou W , Fong MY , Min Y , et al. Cancer‐secreted miR‐105 destroys vascular endothelial barriers to promote metastasis. Cancer Cell. 2014;25(4):501‐515. 24735924 10.1016/j.ccr.2014.03.007 PMC4016197

[148] Zeng Z , Li Y , Pan Y , et al. Cancer‐derived exosomal miR‐25‐3p promotes pre‐metastatic niche formation by inducing vascular permeability and angiogenesis. Nat Commun. 2018;9(1):5395. 30568162 10.1038/s41467-018-07810-w PMC6300604

[149] Yokota Y , Noda T , Okumura Y , et al. Serum exosomal miR‐638 is a prognostic marker of HCC via downregulation of VE‐cadherin and ZO‐1 of endothelial cells. Cancer Sci. 2021;112(3):1275‐1288. 33426736 10.1111/cas.14807 PMC7935782

[150] Liu M , Yang J , Zhang Y , et al. zip4 promotes pancreatic cancer progression by repressing ZO‐1 and Claudin‐1 through a ZEB1‐dependent transcriptional mechanism. Clin Cancer Res. 2018;24(13):3186‐3196. 29615456 10.1158/1078-0432.CCR-18-0263 PMC7006048

[151] Ghosh D , Dutta A , Kashyap A , Upmanyu N , Datta S . PLP2 drives collective cell migration via ZO‐1‐mediated cytoskeletal remodeling at the leading edge in human colorectal cancer cells. J Cell Sci. 2021;134(18):jcs253468. 34409455 10.1242/jcs.253468

[152] Itoh M , Furuse M , Morita K , Kubota K , Saitou M , Tsukita S . Direct binding of three tight junction‐associated MAGUKs, ZO‐1, ZO‐2, and ZO‐3, with the COOH termini of claudins. J Cell Biol. 1999;147(6):1351‐1363. 10601346 10.1083/jcb.147.6.1351 PMC2168087

[153] Delva E , Tucker DK , Kowalczyk AP . The desmosome. Cold Spring Harb Perspect Biol. 2009;1(2):a002543. 20066089 10.1101/cshperspect.a002543 PMC2742091

[154] Dusek RL , Attardi LD . Desmosomes: new perpetrators in tumour suppression. Nat Rev Cancer. 2011;11(5):317‐323. 21508970 10.1038/nrc3051 PMC3799918

[155] Saxena M , Hisano M , Neutzner M , et al. The long non‐coding RNA ET‐20 mediates EMT by impairing desmosomes in breast cancer cells. J Cell Sci. 2021;134(21):jcs258418. 34633031 10.1242/jcs.258418

[156] Chun MG , Hanahan D . Genetic deletion of the desmosomal component desmoplakin promotes tumor microinvasion in a mouse model of pancreatic neuroendocrine carcinogenesis. PLoS Genet. 2010;6(9):e1001120. 20862307 10.1371/journal.pgen.1001120 PMC2940733

[157] Roberts BJ , Johnson KE , McGuinn KP , et al. Palmitoylation of plakophilin is required for desmosome assembly. J Cell Sci. 2014;127(Pt 17):3782‐3793. 25002405 10.1242/jcs.149849 PMC4150063

[158] Lee P , Jiang S , Li Y , et al. Phosphorylation of Pkp1 by RIPK4 regulates epidermal differentiation and skin tumorigenesis. EMBO J. 2017;36(13):1963‐1980. 28507225 10.15252/embj.201695679 PMC5494465

[159] Nath A , Oak A , Chen KY , et al. Palmitate‐Induced IRE1‐XBP1‐ZEB signaling represses desmoplakin expression and promotes cancer cell migration. Mol Cancer Res. 2021;19(2):240‐248. 33106375 10.1158/1541-7786.MCR-19-0480 PMC7864864

[160] Yang L , Chen Y , Cui T , et al. Desmoplakin acts as a tumor suppressor by inhibition of the Wnt/beta‐catenin signaling pathway in human lung cancer. Carcinogenesis. 2012;33(10):1863‐1870. 22791817 10.1093/carcin/bgs226

[161] Chen YJ , Chang JT , Lee L , et al. DSG3 is overexpressed in head neck cancer and is a potential molecular target for inhibition of oncogenesis. Oncogene. 2007;26(3):467‐476. 16878157 10.1038/sj.onc.1209802

[162] Liu YQ , Zou HY , Xie JJ , Fang WK . Paradoxical roles of desmosomal components in head and neck cancer. Biomolecules. 2021;11(6). 10.3390/biom11060914 PMC8234459 34203070

[163] Aasen T , Mesnil M , Naus CC , Lampe PD , Laird DW . Gap junctions and cancer: communicating for 50 years. Nat Rev Cancer. 2016;16(12):775‐788. 27782134 10.1038/nrc.2016.105 PMC5279857

[164] Kyo N , Yamamoto H , Takeda Y , et al. Overexpression of connexin 26 in carcinoma of the pancreas. Oncol Rep. 2008;19(3):627‐631. 18288393

[165] Ezumi K , Yamamoto H , Murata K , et al. Aberrant expression of connexin 26 is associated with lung metastasis of colorectal cancer. Clin Cancer Res. 2008;14(3):677‐684. 18245526 10.1158/1078-0432.CCR-07-1184

[166] Sirnes S , Bruun J , Kolberg M , et al. Connexin43 acts as a colorectal cancer tumor suppressor and predicts disease outcome. Int J Cancer. 2012;131(3):570‐581. 21866551 10.1002/ijc.26392

[167] Sirnes S , Honne H , Ahmed D , et al. DNA methylation analyses of the connexin gene family reveal silencing of GJC1 (Connexin45) by promoter hypermethylation in colorectal cancer. Epigenetics. 2011;6(5):602‐609. 21406965 10.4161/epi.6.5.15237

[168] Benko G , Spajic B , Demirovic A , Stimac G , Tomas D . Prognostic value of connexin43 expression in patients with clinically localized prostate cancer. Prostate Cancer Prostatic Dis. 2011;14(1):90‐95. 21173791 10.1038/pcan.2010.51

[169] Teleki I , Szasz AM , Maros ME , et al. Correlations of differentially expressed gap junction connexins Cx26, Cx30, Cx32, Cx43 and Cx46 with breast cancer progression and prognosis. PLoS One. 2014;9(11):e112541. 25383624 10.1371/journal.pone.0112541 PMC4226536

[170] Poyet C , Buser L , Roudnicky F , et al. Connexin 43 expression predicts poor progression‐free survival in patients with non‐muscle invasive urothelial bladder cancer. J Clin Pathol. 2015;68(10):819‐824. 26251520 10.1136/jclinpath-2015-202898 PMC4602233

[171] Tanaka T , Kimura M , Ishiguro H , Mizoguchi K , Takeyama H . Connexin 43 expression is associated with poor survival in patients with esophageal squamous cell carcinoma. Mol Clin Oncol. 2016;4(6):989‐993. 27284434 10.3892/mco.2016.828 PMC4887812

[172] Brockmeyer P , Jung K , Perske C , Schliephake H , Hemmerlein B . Membrane connexin 43 acts as an independent prognostic marker in oral squamous cell carcinoma. Int J Oncol. 2014;45(1):273‐281. 24788723 10.3892/ijo.2014.2394

[173] Fukuda S , Akiyama M , Harada H , Nakahama KI . Effect of gap junction‐mediated intercellular communication on TGF‐beta induced epithelial‐to‐mesenchymal transition. Biochem Biophys Res Commun. 2019;508(3):928‐933. 30545634 10.1016/j.bbrc.2018.12.027

[174] Venkatesh HS , Morishita W , Geraghty AC , et al. Electrical and synaptic integration of glioma into neural circuits. Nature. 2019;573(7775):539‐545. 31534222 10.1038/s41586-019-1563-y PMC7038898

[175] Noren NK , Niessen CM , Gumbiner BM , Burridge K . Cadherin engagement regulates Rho family GTPases. J Biol Chem. 2001;276(36):33305‐33308. 11457821 10.1074/jbc.C100306200

[176] Ellenbroek SI , Collard JG . Rho GTPases: functions and association with cancer. Clin Exp Metastasis. 2007;24(8):657‐672. 18000759 10.1007/s10585-007-9119-1

[177] Svensmark JH , Brakebusch C . Rho GTPases in cancer: friend or foe?. Oncogene. 2019;38(50):7447‐7456. 31427738 10.1038/s41388-019-0963-7

[178] Orgaz JL , Herraiz C , Sanz‐Moreno V . Rho GTPases modulate malignant transformation of tumor cells. Small GTPases. 2014;5:e29019. 25036871 10.4161/sgtp.29019 PMC4125382

[179] Fritz G , Just I , Kaina B . Rho GTPases are over‐expressed in human tumors. Int J Cancer. 1999;81(5):682‐687. 10328216 10.1002/(sici)1097-0215(19990531)81:5<682::aid-ijc2>3.0.co;2-b

[180] Horiuchi A , Imai T , Wang C , et al. Up‐regulation of small GTPases, RhoA and RhoC, is associated with tumor progression in ovarian carcinoma. Lab Invest. 2003;83(6):861‐870. 12808121 10.1097/01.lab.0000073128.16098.31

[181] Pan Y , Bi F , Liu N , et al. Expression of seven main Rho family members in gastric carcinoma. Biochem Biophys Res Commun. 2004;315(3):686‐691. 14975755 10.1016/j.bbrc.2004.01.108

[182] Engers R , Ziegler S , Mueller M , Walter A , Willers R , Gabbert HE . Prognostic relevance of increased Rac GTPase expression in prostate carcinomas. Endocr Relat Cancer. 2007;14(2):245‐256. 17639041 10.1677/ERC-06-0036

[183] Wang J , Rao Q , Wang M , et al. Overexpression of Rac1 in leukemia patients and its role in leukemia cell migration and growth. Biochem Biophys Res Commun. 2009;386(4):769‐774. 19563775 10.1016/j.bbrc.2009.06.125

[184] Gomez Del Pulgar T , Valdes‐Mora F , Bandres E , et al. Cdc42 is highly expressed in colorectal adenocarcinoma and downregulates ID4 through an epigenetic mechanism. Int J Oncol. 2008;33(1):185‐193. 18575765

[185] Liu Y , Wang Y , Zhang Y , et al. Abnormal expression of p120‐catenin, E‐cadherin, and small GTPases is significantly associated with malignant phenotype of human lung cancer. Lung Cancer. 2009;63(3):375‐382. 19162367 10.1016/j.lungcan.2008.12.012

[186] Tucci MG , Lucarini G , Brancorsini D , et al. Involvement of E‐cadherin, beta‐catenin, Cdc42 and CXCR4 in the progression and prognosis of cutaneous melanoma. Br J Dermatol. 2007;157(6):1212‐1216. 17970806 10.1111/j.1365-2133.2007.08246.x

[187] Molinie N , Gautreau A . The Arp2/3 regulatory system and its deregulation in cancer. Physiol Rev. 2018;98(1):215‐238. 29212790 10.1152/physrev.00006.2017

[188] Ridley AJ . Rho GTPase signalling in cell migration. Curr Opin Cell Biol. 2015;36:103‐112. 26363959 10.1016/j.ceb.2015.08.005 PMC4728192

[189] Carlier MF , Ducruix A , Pantaloni D . Signalling to actin: the Cdc42‐N‐WASP‐Arp2/3 connection. Chem Biol. 1999;6(9):R235‐R240. 10467124 10.1016/s1074-5521(99)80107-0

[190] Zhang X , Moore SW , Iskratsch T , Sheetz MP . N‐WASP‐directed actin polymerization activates Cas phosphorylation and lamellipodium spreading. J Cell Sci. 2014;127(Pt 7):1394‐1405. 24481817 10.1242/jcs.134692 PMC3970555

[191] Peng JM , Bera R , Chiou CY , et al. Actin cytoskeleton remodeling drives epithelial‐mesenchymal transition for hepatoma invasion and metastasis in mice. Hepatology. 2018;67(6):2226‐2243. 29171033 10.1002/hep.29678

[192] Zhang YL , Li Q , Yang XM , et al. SPON2 promotes M1‐like macrophage recruitment and inhibits hepatocellular carcinoma metastasis by distinct integrin‐Rho GTPase‐Hippo pathways. Cancer Res. 2018;78(9):2305‐2317. 29440144 10.1158/0008-5472.CAN-17-2867

[193] Xie Z , Janczyk PL , Zhang Y , et al. A cytoskeleton regulator AVIL drives tumorigenesis in glioblastoma. Nat Commun. 2020;11(1):3457. 32651364 10.1038/s41467-020-17279-1 PMC7351761

[194] Armacki M , Polaschek S , Waldenmaier M , et al. Protein kinase D1, reduced in human pancreatic tumors, increases secretion of small extracellular vesicles from cancer cells that promote metastasis to lung in mice. Gastroenterology. 2020;159(3):1019‐1035. e22. 32446697 10.1053/j.gastro.2020.05.052

[195] Ge W , Goga A , He Y , et al. miR‐802 suppresses acinar‐to‐ductal reprogramming during early pancreatitis and pancreatic carcinogenesis. Gastroenterology. 2022;162(1):269‐284. 34547282 10.1053/j.gastro.2021.09.029

[196] Liu D , Qiu XY , Wu X , et al. Piperlongumine suppresses bladder cancer invasion via inhibiting epithelial mesenchymal transition and F‐actin reorganization. Biochem Biophys Res Commun. 2017;494(1‐2):165‐172. 29037814 10.1016/j.bbrc.2017.10.061

[197] Zheng L , Xiang C , Li X , et al. STARD13‐correlated ceRNA network‐directed inhibition on YAP/TAZ activity suppresses stemness of breast cancer via co‐regulating Hippo and Rho‐GTPase/F‐actin signaling. J Hematol Oncol. 2018;11(1):72. 29848346 10.1186/s13045-018-0613-5 PMC5977742

[198] Lee S , Kang H , Shin E , Jeon J , Youn H , Youn B . BEX1 and BEX4 induce GBM progression through regulation of actin polymerization and activation of YAP/TAZ signaling. Int J Mol Sci. 2021;22(18):9845. 34576008 10.3390/ijms22189845 PMC8471324

[199] Sayed KA , Khanfar MA , Shallal HM , et al. Latrunculin A and its C‐17‐O‐carbamates inhibit prostate tumor cell invasion and HIF‐1 activation in breast tumor cells. J Nat Prod. 2008;71(3):396‐402. 18298079 10.1021/np070587w PMC2930178

[200] Shin IJ , Ahn YT , Kim Y , Kim JM , An WG . Actin disruption agents induce phosphorylation of histone H2AX in human breast adenocarcinoma MCF‐7 cells. Oncol Rep. 2011;25(5):1313‐1319. 21399880 10.3892/or.2011.1214

[201] Alblazi KM , Siar CH . Cellular protrusions–lamellipodia, filopodia, invadopodia and podosomes–and their roles in progression of orofacial tumours: current understanding. Asian Pac J Cancer Prev. 2015;16(6):2187‐2191. 25824735 10.7314/apjcp.2015.16.6.2187

[202] Sibony‐Benyamini H , Gil‐Henn H . Invadopodia: the leading force. Eur J Cell Biol. 2012;91(11‐12):896‐901. 22633185 10.1016/j.ejcb.2012.04.001

[203] Weaver AM . Invadopodia. Curr Biol. 2008;18(9):R362‐R364. 18460310 10.1016/j.cub.2008.02.028

[204] Murphy DA , Courtneidge SA . The ‘ins’ and ‘outs’ of podosomes and invadopodia: characteristics, formation and function. Nat Rev Mol Cell Biol. 2011;12(7):413‐426. 21697900 10.1038/nrm3141 PMC3423958

[205] Shibue T , Brooks MW , Weinberg RA . An integrin‐linked machinery of cytoskeletal regulation that enables experimental tumor initiation and metastatic colonization. Cancer Cell. 2013;24(4):481‐198. 24035453 10.1016/j.ccr.2013.08.012 PMC3864118

[206] Peng JM , Lin SH , Yu MC , Hsieh SY . CLIC1 recruits PIP5K1A/C to induce cell‐matrix adhesions for tumor metastasis. J Clin Invest. 2021;131(1):e133525. 10.1172/JCI133525 PMC7773349 33079727

[207] Bi X , Lou P , Song Y , et al. Msi1 promotes breast cancer metastasis by regulating invadopodia‐mediated extracellular matrix degradation via the Timp3‐Mmp9 pathway. Oncogene. 2021;40(29):4832‐4845. 34155343 10.1038/s41388-021-01873-8

[208] Chen Z , He S , Zhan Y , et al. TGF‐beta‐induced transgelin promotes bladder cancer metastasis by regulating epithelial‐mesenchymal transition and invadopodia formation. EBioMedicine. 2019;47:208‐220. 31420300 10.1016/j.ebiom.2019.08.012 PMC6796540

[209] Eddy RJ , Weidmann MD , Sharma VP , Condeelis JS . Tumor cell invadopodia: invasive protrusions that orchestrate metastasis. Trends Cell Biol. 2017;27(8):595‐607. 28412099 10.1016/j.tcb.2017.03.003 PMC5524604

[210] Meirson T , Gil‐Henn H . Targeting invadopodia for blocking breast cancer metastasis. Drug Resist Updat. 2018;39:1‐17. 30075834 10.1016/j.drup.2018.05.002

[211] Yoon JR , Whipple RA , Balzer EM , et al. Local anesthetics inhibit kinesin motility and microtentacle protrusions in human epithelial and breast tumor cells. Breast Cancer Res Treat. 2011;129(3):691‐701. 21069453 10.1007/s10549-010-1239-7 PMC4232214

[212] Peinado H , Olmeda D , Cano A . Snail, Zeb and bHLH factors in tumour progression: an alliance against the epithelial phenotype?. Nat Rev Cancer. 2007;7(6):415‐428. 17508028 10.1038/nrc2131

[213] Stemmler MP , Eccles RL , Brabletz S , Brabletz T . Non‐redundant functions of EMT transcription factors. Nat Cell Biol. 2019;21(1):102‐112. 30602760 10.1038/s41556-018-0196-y

[214] Tan TZ , Miow QH , Miki Y , et al. Epithelial‐mesenchymal transition spectrum quantification and its efficacy in deciphering survival and drug responses of cancer patients. EMBO Mol Med. 2014;6(10):1279‐1293. 25214461 10.15252/emmm.201404208 PMC4287932

[215] Larsen JE , Nathan V , Osborne JK , et al. ZEB1 drives epithelial‐to‐mesenchymal transition in lung cancer. J Clin Invest. 2016;126(9):3219‐3235. 27500490 10.1172/JCI76725 PMC5004933

[216] Liu M , Zhang Y , Yang J , et al. Zinc‐dependent regulation of ZEB1 and YAP1 coactivation promotes epithelial‐mesenchymal transition plasticity and metastasis in pancreatic cancer. Gastroenterology. 2021;160(5):1771‐1783. e1. 33421513 10.1053/j.gastro.2020.12.077 PMC8035249

[217] Almotiri A , Alzahrani H , Menendez‐Gonzalez JB , et al. Zeb1 modulates hematopoietic stem cell fates required for suppressing acute myeloid leukemia. J Clin Invest. 2021;131(1):e129115. 10.1172/JCI129115 PMC7773410 33108352

[218] Zhang T , Guo L , Creighton CJ , et al. A genetic cell context‐dependent role for ZEB1 in lung cancer. Nat Commun. 2016;7:12231. 27456471 10.1038/ncomms12231 PMC4963474

[219] Denecker G , Vandamme N , Akay O , et al. Identification of a ZEB2‐MITF‐ZEB1 transcriptional network that controls melanogenesis and melanoma progression. Cell Death Differ. 2014;21(8):1250‐1261. 24769727 10.1038/cdd.2014.44 PMC4085532

[220] Hultgren NW , Fang JS , Ziegler ME , et al. Slug regulates the Dll4‐Notch‐VEGFR2 axis to control endothelial cell activation and angiogenesis. Nat Commun. 2020;11(1):5400. 33106502 10.1038/s41467-020-18633-z PMC7588439

[221] Tran DD , Corsa CA , Biswas H , Aft RL , Longmore GD . Temporal and spatial cooperation of Snail1 and Twist1 during epithelial‐mesenchymal transition predicts for human breast cancer recurrence. Mol Cancer Res. 2011;9(12):1644‐1657. 22006115 10.1158/1541-7786.MCR-11-0371 PMC4922748

[222] Lin Y , Dong C , Zhou BP . Epigenetic regulation of EMT: the Snail story. Curr Pharm Des. 2014;20(11):1698‐1705. 23888971 10.2174/13816128113199990512 PMC4005722

[223] Dong C , Wu Y , Yao J , et al. G9a interacts with Snail and is critical for Snail‐mediated E‐cadherin repression in human breast cancer. J Clin Invest. 2012;122(4):1469‐1486. 22406531 10.1172/JCI57349 PMC3314447

[224] Vincent T , Neve EP , Johnson JR , et al. A SNAIL1‐SMAD3/4 transcriptional repressor complex promotes TGF‐beta mediated epithelial‐mesenchymal transition. Nat Cell Biol. 2009;11(8):943‐950. 19597490 10.1038/ncb1905 PMC3769970

[225] Kim BJ , Hancock BM , Bermudez A , et al. Bacterial induction of Snail1 contributes to blood‐brain barrier disruption. J Clin Invest. 2015;125(6):2473‐2483. 25961453 10.1172/JCI74159 PMC4497739

[226] Wroblewski LE , Piazuelo MB , Chaturvedi R , et al. Helicobacter pylori targets cancer‐associated apical‐junctional constituents in gastroids and gastric epithelial cells. Gut. 2015;64(5):720‐730. 25123931 10.1136/gutjnl-2014-307650 PMC4329117

[227] Zheng Y , Huang G , Silva TC , et al. A pan‐cancer analysis of CpG Island gene regulation reveals extensive plasticity within Polycomb target genes. Nat Commun. 2021;12(1):2485. 33931649 10.1038/s41467-021-22720-0 PMC8087678

[228] Wu J , Chai H , Shan H , et al. Histone methyltransferase SETD1A induces epithelial‐mesenchymal transition to promote invasion and metastasis through epigenetic reprogramming of snail in gastric cancer. Front Cell Dev Biol. 2021;9:657888. 34164392 10.3389/fcell.2021.657888 PMC8215546

[229] Cock‐Rada AM , Medjkane S , Janski N , et al. SMYD3 promotes cancer invasion by epigenetic upregulation of the metalloproteinase MMP‐9. Cancer Res. 2012;72(3):810‐820. 22194464 10.1158/0008-5472.CAN-11-1052 PMC3299564

[230] Peinado H , Ballestar E , Esteller M , Cano A . Snail mediates E‐cadherin repression by the recruitment of the Sin3A/histone deacetylase 1 (HDAC1)/HDAC2 complex. Mol Cell Biol. 2004;24(1):306‐319. 14673164 10.1128/MCB.24.1.306-319.2004 PMC303344

[231] Shi Y , Lan F , Matson C , et al. Histone demethylation mediated by the nuclear amine oxidase homolog LSD1. Cell. 2004;119(7):941‐953. 15620353 10.1016/j.cell.2004.12.012

[232] Shi YJ , Matson C , Lan F , Iwase S , Baba T , Shi Y . Regulation of LSD1 histone demethylase activity by its associated factors. Mol Cell. 2005;19(6):857‐864. 16140033 10.1016/j.molcel.2005.08.027

[233] Zhang N , Zhang H , Liu Y , et al. SREBP1, targeted by miR‐18a‐5p, modulates epithelial‐mesenchymal transition in breast cancer via forming a co‐repressor complex with Snail and HDAC1/2. Cell Death Differ. 2019;26(5):843‐859. 29988076 10.1038/s41418-018-0158-8 PMC6461794

[234] Huang W , Chen J , Liu X , et al. MIER3 induces epithelial‐mesenchymal transition and promotes breast cancer cell aggressiveness via forming a co‐repressor complex with HDAC1/HDAC2/Snail. Exp Cell Res. 2021;406(1):112722. 34242623 10.1016/j.yexcr.2021.112722

[235] von Burstin J , Eser S , Paul MC , et al. E‐cadherin regulates metastasis of pancreatic cancer in vivo and is suppressed by a SNAIL/HDAC1/HDAC2 repressor complex. Gastroenterology. 2009;137(1):361‐371. e1‐5. 19362090 10.1053/j.gastro.2009.04.004

[236] Tong ZT , Cai MY , Wang XG , et al. EZH2 supports nasopharyngeal carcinoma cell aggressiveness by forming a co‐repressor complex with HDAC1/HDAC2 and Snail to inhibit E‐cadherin. Oncogene. 2012;31(5):583‐594. 21685935 10.1038/onc.2011.254

[237] Barbera MJ , Puig I , Dominguez D , et al. Regulation of Snail transcription during epithelial to mesenchymal transition of tumor cells. Oncogene. 2004;23(44):7345‐7354. 15286702 10.1038/sj.onc.1207990

[238] Palmer MB , Majumder P , Cooper JC , Yoon H , Wade PA , Boss JM . Yin yang 1 regulates the expression of snail through a distal enhancer. Mol Cancer Res. 2009;7(2):221‐229. 19208738 10.1158/1541-7786.MCR-08-0229 PMC2819842

[239] Peiro S , Escriva M , Puig I , et al. Snail1 transcriptional repressor binds to its own promoter and controls its expression. Nucleic Acids Res. 2006;34(7):2077‐2084. 16617148 10.1093/nar/gkl141 PMC1440880

[240] Noce V , Battistelli C , Cozzolino AM , et al. YAP integrates the regulatory Snail/HNF4alpha circuitry controlling epithelial/hepatocyte differentiation. Cell Death Dis. 2019;10(10):768. 31601778 10.1038/s41419-019-2000-8 PMC6787001

[241] Li X , Deng W , Nail CD , et al. Snail induction is an early response to Gli1 that determines the efficiency of epithelial transformation. Oncogene. 2006;25(4):609‐621. 16158046 10.1038/sj.onc.1209077 PMC1361531

[242] Leng Z , Li Y , Zhou G , et al. Kruppel‐like factor 4 regulates stemness and mesenchymal properties of colorectal cancer stem cells through the TGF‐beta1/Smad/snail pathway. J Cell Mol Med. 2020;24(2):1866‐1877. 31830379 10.1111/jcmm.14882 PMC6991673

[243] Lin X , Chai G , Wu Y , et al. RNA m(6)A methylation regulates the epithelial mesenchymal transition of cancer cells and translation of Snail. Nat Commun. 2019;10(1):2065. 31061416 10.1038/s41467-019-09865-9 PMC6502834

[244] Wang X , Liu R , Zhu W , et al. UDP‐glucose accelerates SNAI1 mRNA decay and impairs lung cancer metastasis. Nature. 2019;571(7763):127‐131. 31243371 10.1038/s41586-019-1340-y

[245] Zhou BP , Deng J , Xia W , et al. Dual regulation of Snail by GSK‐3beta‐mediated phosphorylation in control of epithelial‐mesenchymal transition. Nat Cell Biol. 2004;6(10):931‐940. 15448698 10.1038/ncb1173

[246] Jung HY , Fattet L , Tsai JH , et al. Apical‐basal polarity inhibits epithelial‐mesenchymal transition and tumour metastasis by PAR‐complex‐mediated SNAI1 degradation. Nat Cell Biol. 2019;21(3):359‐371. 30804505 10.1038/s41556-019-0291-8 PMC6546105

[247] Fan L , Chen Z , Wu X , et al. Ubiquitin‐specific protease 3 promotes glioblastoma cell invasion and epithelial‐mesenchymal transition via stabilizing snail. Mol Cancer Res. 2019;17(10):1975‐1984. 31266817 10.1158/1541-7786.MCR-19-0197

[248] Wu Y , Deng J , Rychahou PG , Qiu S , Evers BM , Zhou BP . Stabilization of snail by NF‐kappaB is required for inflammation‐induced cell migration and invasion. Cancer Cell. 2009;15(5):416‐428. 19411070 10.1016/j.ccr.2009.03.016 PMC2881229

[249] Meng J , Ai X , Lei Y , et al. USP5 promotes epithelial‐mesenchymal transition by stabilizing SLUG in hepatocellular carcinoma. Theranostics. 2019;9(2):573‐587. 30809294 10.7150/thno.27654 PMC6376178

[250] Ouchida AT , Kacal M , Zheng A , et al. USP10 regulates the stability of the EMT‐transcription factor Slug/SNAI2. Biochem Biophys Res Commun. 2018;502(4):429‐434. 29803676 10.1016/j.bbrc.2018.05.156

[251] Li W , Shen M , Jiang YZ , et al. Deubiquitinase USP20 promotes breast cancer metastasis by stabilizing SNAI2. Genes Dev. 2020;34(19‐20):1310‐1315. 32943575 10.1101/gad.339804.120 PMC7528704

[252] Hung PF , Hong TM , Chang CC , et al. Hypoxia‐induced Slug SUMOylation enhances lung cancer metastasis. J Exp Clin Cancer Res. 2019;38(1):5. 30612578 10.1186/s13046-018-0996-8 PMC6322271

[253] Qin Q , Xu Y , He T , Qin C , Xu J . Normal and disease‐related biological functions of Twist1 and underlying molecular mechanisms. Cell Res. 2012;22(1):90‐106. 21876555 10.1038/cr.2011.144 PMC3351934

[254] Franco HL , Casasnovas J , Rodriguez‐Medina JR , Cadilla CL . Redundant or separate entities?–roles of Twist1 and Twist2 as molecular switches during gene transcription. Nucleic Acids Res. 2011;39(4):1177‐1186. 20935057 10.1093/nar/gkq890 PMC3045590

[255] Xu Y , Lee DK , Feng Z , et al. Breast tumor cell‐specific knockout of Twist1 inhibits cancer cell plasticity, dissemination, and lung metastasis in mice. Proc Natl Acad Sci USA. 2017;114(43):11494‐11499. 29073077 10.1073/pnas.1618091114 PMC5664488

[256] Kang Y , Massague J . Epithelial‐mesenchymal transitions: twist in development and metastasis. Cell. 2004;118(3):277‐279. 15294153 10.1016/j.cell.2004.07.011

[257] Cakouros D , Isenmann S , Cooper L , et al. Twist‐1 induces Ezh2 recruitment regulating histone methylation along the Ink4A/Arf locus in mesenchymal stem cells. Mol Cell Biol. 2012;32(8):1433‐1441. 22290439 10.1128/MCB.06315-11 PMC3318575

[258] Yang F , Sun L , Li Q , et al. SET8 promotes epithelial‐mesenchymal transition and confers TWIST dual transcriptional activities. EMBO J. 2012;31(1):110‐123. 21983900 10.1038/emboj.2011.364 PMC3252577

[259] Yang MH , Hsu DS , Wang HW , et al. Bmi1 is essential in Twist1‐induced epithelial‐mesenchymal transition. Nat Cell Biol. 2010;12(10):982‐992. 20818389 10.1038/ncb2099

[260] Xu Y , Qin L , Sun T , et al. Twist1 promotes breast cancer invasion and metastasis by silencing Foxa1 expression. Oncogene. 2017;36(8):1157‐1166. 27524420 10.1038/onc.2016.286 PMC5311074

[261] Selmi A , de Saint‐Jean M , Jallas AC , et al. TWIST1 is a direct transcriptional target of MYCN and MYC in neuroblastoma. Cancer Lett. 2015;357(1):412‐418. 25475555 10.1016/j.canlet.2014.11.056

[262] Huang W , Chen Z , Shang X , et al. Sox12, a direct target of FoxQ1, promotes hepatocellular carcinoma metastasis through up‐regulating Twist1 and FGFBP1. Hepatology. 2015;61(6):1920‐1933. 25704764 10.1002/hep.27756

[263] Feng M , Fang F , Fang T , et al. Sox13 promotes hepatocellular carcinoma metastasis by transcriptionally activating Twist1. Lab Invest. 2020;100(11):1400‐1410. 32461589 10.1038/s41374-020-0445-0

[264] Asanoma K , Liu G , Yamane T , et al. Regulation of the mechanism of TWIST1 transcription by BHLHE40 and BHLHE41 in cancer cells. Mol Cell Biol. 2015;35(24):4096‐4109. 26391953 10.1128/MCB.00678-15 PMC4648814

[265] Hu Q , Li C , Wang S , et al. LncRNAs‐directed PTEN enzymatic switch governs epithelial‐mesenchymal transition. Cell Res. 2019;29(4):286‐304. 30631154 10.1038/s41422-018-0134-3 PMC6461864

[266] Yang‐Hartwich Y , Tedja R , Roberts CM , et al. p53‐Pirh2 complex promotes Twist1 degradation and inhibits EMT. Mol Cancer Res. 2019;17(1):153‐164. 30131448 10.1158/1541-7786.MCR-18-0238 PMC6800184

[267] Lee HJ , Li CF , Ruan D , et al. The DNA damage transducer RNF8 facilitates cancer chemoresistance and progression through twist activation. Mol Cell. 2016;63(6):1021‐1033. 27618486 10.1016/j.molcel.2016.08.009 PMC5026628

[268] Tedja R , Roberts CM , Alvero AB , et al. Protein kinase Calpha‐mediated phosphorylation of Twist1 at Ser‐144 prevents Twist1 ubiquitination and stabilizes it. J Biol Chem. 2019;294(13):5082‐5093. 30733340 10.1074/jbc.RA118.005921 PMC6442047

[269] Li CW , Xia W , Lim SO , et al. AKT1 inhibits epithelial‐to‐mesenchymal transition in breast cancer through phosphorylation‐dependent twist1 degradation. Cancer Res. 2016;76(6):1451‐1462. 26759241 10.1158/0008-5472.CAN-15-1941 PMC4794388

[270] Wei SC , Fattet L , Tsai JH , et al. Matrix stiffness drives epithelial‐mesenchymal transition and tumour metastasis through a TWIST1‐G3BP2 mechanotransduction pathway. Nat Cell Biol. 2015;17(5):678‐688. 25893917 10.1038/ncb3157 PMC4452027

[271] Fattet L , Jung HY , Matsumoto MW , et al. Matrix rigidity controls epithelial‐mesenchymal plasticity and tumor metastasis via a mechanoresponsive EPHA2/LYN complex. Dev Cell. 2020;54(3):302‐316. e7. 32574556 10.1016/j.devcel.2020.05.031 PMC7423770

[272] Parajuli P , Singh P , Wang Z , et al. TGIF1 functions as a tumor suppressor in pancreatic ductal adenocarcinoma. EMBO J. 2019;38(13):e101067. 31268604 10.15252/embj.2018101067 PMC6601038

[273] Palumbo‐Zerr K , Soare A , Zerr P , et al. Composition of TWIST1 dimers regulates fibroblast activation and tissue fibrosis. Ann Rheum Dis. 2017;76(1):244‐251. 27113414 10.1136/annrheumdis-2015-208470

[274] Connerney J , Andreeva V , Leshem Y , Muentener C , Mercado MA , Spicer DB . Twist1 dimer selection regulates cranial suture patterning and fusion. Dev Dyn. 2006;235(5):1345‐1357. 16502419 10.1002/dvdy.20717

[275] Li H , Xu L , Li C , et al. Ubiquitin ligase Cbl‐b represses IGF‐I‐induced epithelial mesenchymal transition via ZEB2 and microRNA‐200c regulation in gastric cancer cells. Mol Cancer. 2014;13:136. 24885194 10.1186/1476-4598-13-136 PMC4052283

[276] Li J , Riedt T , Goossens S , et al. The EMT transcription factor Zeb2 controls adult murine hematopoietic differentiation by regulating cytokine signaling. Blood. 2017;129(4):460‐472. 27683414 10.1182/blood-2016-05-714659

[277] Miro C , Di Cicco E , Ambrosio R , et al. Thyroid hormone induces progression and invasiveness of squamous cell carcinomas by promoting a ZEB‐1/E‐cadherin switch. Nat Commun. 2019;10(1):5410. 31776338 10.1038/s41467-019-13140-2 PMC6881453

[278] Raggi C , Factor VM , Seo D , et al. Epigenetic reprogramming modulates malignant properties of human liver cancer. Hepatology. 2014;59(6):2251‐2262. 24449497 10.1002/hep.27026 PMC4043911

[279] Postigo AA , Depp JL , Taylor JJ , Kroll KL . Regulation of Smad signaling through a differential recruitment of coactivators and corepressors by ZEB proteins. EMBO J. 2003;22(10):2453‐2462. 12743039 10.1093/emboj/cdg226 PMC155984

[280] Sanchez‐Tillo E , Lazaro A , Torrent R , et al. ZEB1 represses E‐cadherin and induces an EMT by recruiting the SWI/SNF chromatin‐remodeling protein BRG1. Oncogene. 2010;29(24):3490‐3500. 20418909 10.1038/onc.2010.102

[281] Aghdassi A , Sendler M , Guenther A , et al. Recruitment of histone deacetylases HDAC1 and HDAC2 by the transcriptional repressor ZEB1 downregulates E‐cadherin expression in pancreatic cancer. Gut. 2012;61(3):439‐448. 22147512 10.1136/gutjnl-2011-300060

[282] Manshouri R , Coyaud E , Kundu ST , et al. ZEB1/NuRD complex suppresses TBC1D2b to stimulate E‐cadherin internalization and promote metastasis in lung cancer. Nat Commun. 2019;10(1):5125. 31719531 10.1038/s41467-019-12832-z PMC6851102

[283] Si W , Huang W , Zheng Y , et al. Dysfunction of the reciprocal feedback loop between GATA3‐ and ZEB2‐nucleated repression programs contributes to breast cancer metastasis. Cancer Cell. 2015;27(6):822‐836. 26028330 10.1016/j.ccell.2015.04.011

[284] Wu LM , Wang J , Conidi A , et al. Zeb2 recruits HDAC‐NuRD to inhibit Notch and controls Schwann cell differentiation and remyelination. Nat Neurosci. 2016;19(8):1060‐1072. 27294509 10.1038/nn.4322 PMC4961522

[285] Dave N , Guaita‐Esteruelas S , Gutarra S , et al. Functional cooperation between Snail1 and twist in the regulation of ZEB1 expression during epithelial to mesenchymal transition. J Biol Chem. 2011;286(14):12024‐12032. 21317430 10.1074/jbc.M110.168625 PMC3069405

[286] Xiao Q , Gan Y , Li Y , et al. MEF2A transcriptionally upregulates the expression of ZEB2 and CTNNB1 in colorectal cancer to promote tumor progression. Oncogene. 2021;40(19):3364‐3377. 33863999 10.1038/s41388-021-01774-w PMC8116210

[287] Llorens MC , Lorenzatti G , Cavallo NL , et al. Phosphorylation regulates functions of ZEB1 transcription factor. J Cell Physiol. 2016;231(10):2205‐2217. 26868487 10.1002/jcp.25338 PMC5902805

[288] Chiu LY , Hsin IL , Yang TY , et al. The ERK‐ZEB1 pathway mediates epithelial‐mesenchymal transition in pemetrexed resistant lung cancer cells with suppression by vinca alkaloids. Oncogene. 2017;36(2):242‐253. 27270426 10.1038/onc.2016.195 PMC5241427

[289] Aceto N , Sausgruber N , Brinkhaus H , et al. Tyrosine phosphatase SHP2 promotes breast cancer progression and maintains tumor‐initiating cells via activation of key transcription factors and a positive feedback signaling loop. Nat Med. 2012;18(4):529‐537. 22388088 10.1038/nm.2645

[290] Cui Y , Liang S , Zhang S , et al. ABCA8 is regulated by miR‐374b‐5p and inhibits proliferation and metastasis of hepatocellular carcinoma through the ERK/ZEB1 pathway. J Exp Clin Cancer Res. 2020;39(1):90. 32430024 10.1186/s13046-020-01591-1 PMC7236190

[291] Graham TR , Zhau HE , Odero‐Marah VA , et al. Insulin‐like growth factor‐I‐dependent up‐regulation of ZEB1 drives epithelial‐to‐mesenchymal transition in human prostate cancer cells. Cancer Res. 2008;68(7):2479‐2488. 18381457 10.1158/0008-5472.CAN-07-2559

[292] Lau MT , So WK , Leung PC . Fibroblast growth factor 2 induces E‐cadherin down‐regulation via PI3K/Akt/mTOR and MAPK/ERK signaling in ovarian cancer cells. PLoS One. 2013;8(3):e59083. 23554977 10.1371/journal.pone.0059083 PMC3598697

[293] Jimenez‐Pascual A , Hale JS , Kordowski A , et al. ADAMDEC1 maintains a growth factor signaling loop in cancer stem cells. Cancer Discov. 2019;9(11):1574‐1589. 31434712 10.1158/2159-8290.CD-18-1308 PMC7400732

[294] Sinh ND , Endo K , Miyazawa K , Saitoh M . Ets1 and ESE1 reciprocally regulate expression of ZEB1/ZEB2, dependent on ERK1/2 activity, in breast cancer cells. Cancer Sci. 2017;108(5):952‐960. 28247944 10.1111/cas.13214 PMC5448599

[295] Zhang P , Wei Y , Wang L , et al. ATM‐mediated stabilization of ZEB1 promotes DNA damage response and radioresistance through CHK1. Nat Cell Biol. 2014;16(9):864‐875. 25086746 10.1038/ncb3013 PMC4150825

[296] Li X , Yuan J , Song C , et al. Deubiquitinase USP39 and E3 ligase TRIM26 balance the level of ZEB1 ubiquitination and thereby determine the progression of hepatocellular carcinoma. Cell Death Differ. 2021;28(8):2315‐2332. 33649471 10.1038/s41418-021-00754-7 PMC8329202

[297] Luo H , Zhou Z , Huang S , et al. CHFR regulates chemoresistance in triple‐negative breast cancer through destabilizing ZEB1. Cell Death Dis. 2021;12(9):820. 34462429 10.1038/s41419-021-04114-8 PMC8405615

[298] Kim HY , Kim YM , Hong S . DNAJB9 suppresses the metastasis of triple‐negative breast cancer by promoting FBXO45‐mediated degradation of ZEB1. Cell Death Dis. 2021;12(5):461. 33966034 10.1038/s41419-021-03757-x PMC8106677

[299] Zhou Z , Zhang P , Hu X , et al. USP51 promotes deubiquitination and stabilization of ZEB1. Am J Cancer Res. 2017;7(10):2020‐2031. 29119051 PMC5665849

[300] Ye DX , Wang SS , Huang Y , Wang XJ , Chi P . USP43 directly regulates ZEB1 protein, mediating proliferation and metastasis of colorectal cancer. J Cancer. 2021;12(2):404‐416. 33391437 10.7150/jca.48056 PMC7738986

[301] Li N , Babaei‐Jadidi R , Lorenzi F , et al. An FBXW7‐ZEB2 axis links EMT and tumour microenvironment to promote colorectal cancer stem cells and chemoresistance. Oncogenesis. 2019;8(3):13. 30783098 10.1038/s41389-019-0125-3 PMC6381143

[302] Feng S , Cai X , Li Y , Jian X , Zhang L , Li B . Tripartite motif‐containing 14 (TRIM14) promotes epithelial‐mesenchymal transition via ZEB2 in glioblastoma cells. J Exp Clin Cancer Res. 2019;38(1):57. 30728039 10.1186/s13046-019-1070-x PMC6364431

[303] Shi L , Tang X , Qian M , et al. A SIRT1‐centered circuitry regulates breast cancer stemness and metastasis. Oncogene. 2018;37(49):6299‐6315. 30038266 10.1038/s41388-018-0370-5 PMC6283862

[304] Takano S , Reichert M , Bakir B , et al. Prrx1 isoform switching regulates pancreatic cancer invasion and metastatic colonization. Genes Dev. 2016;30(2):233‐247. 26773005 10.1101/gad.263327.115 PMC4719312

[305] Grimm D , Bauer J , Wise P , et al. The role of SOX family members in solid tumours and metastasis. Semin Cancer Biol. 2020;67(Pt 1):122‐153. 30914279 10.1016/j.semcancer.2019.03.004

[306] Inoue H , Takahashi H , Hashimura M , et al. Cooperation of Sox4 with beta‐catenin/p300 complex in transcriptional regulation of the Slug gene during divergent sarcomatous differentiation in uterine carcinosarcoma. BMC Cancer. 2016;16:53. 26841870 10.1186/s12885-016-2090-y PMC4739330

[307] Klatt Shaw D , Saraswathy VM , Zhou L , et al. Localized EMT reprograms glial progenitors to promote spinal cord repair. Dev Cell. 2021;56(5):613‐626. e7. 33609461 10.1016/j.devcel.2021.01.017 PMC8044706

[308] Eichmuller OL , Corsini NS , Vertesy A , et al. Amplification of human interneuron progenitors promotes brain tumors and neurological defects. Science. 2022;375(6579):eabf5546. 35084981 10.1126/science.abf5546 PMC7613689

[309] Ocana OH , Coskun H , Minguillon C , et al. A right‐handed signalling pathway drives heart looping in vertebrates. Nature. 2017;549(7670):86‐90. 28880281 10.1038/nature23454 PMC5590727

[310] Rago L , Castroviejo N , Fazilaty H , et al. MicroRNAs establish the right‐handed dominance of the heart laterality pathway in vertebrates. Dev Cell. 2019;51(4):446‐459. e5. 31630980 10.1016/j.devcel.2019.09.012

[311] Zinski J , Tajer B , Mullins MC . TGF‐beta family signaling in early vertebrate development. Cold Spring Harb Perspect Biol. 2018;10(6):a033274. 28600394 10.1101/cshperspect.a033274 PMC5983195

[312] Clevers H . Wnt/beta‐catenin signaling in development and disease. Cell. 2006;127(3):469‐480. 17081971 10.1016/j.cell.2006.10.018

[313] van den Brink GR . Hedgehog signaling in development and homeostasis of the gastrointestinal tract. Physiol Rev. 2007;87(4):1343‐1375. 17928586 10.1152/physrev.00054.2006

[314] Wu Z , Guan KL . Hippo signaling in embryogenesis and development. Trends Biochem Sci. 2021;46(1):51‐63. 32928629 10.1016/j.tibs.2020.08.008 PMC7749079

[315] Shi Y , Massague J . Mechanisms of TGF‐beta signaling from cell membrane to the nucleus. Cell. 2003;113(6):685‐700. 12809600 10.1016/s0092-8674(03)00432-x

[316] Derynck R , Turley SJ , Akhurst RJ . TGFbeta biology in cancer progression and immunotherapy. Nat Rev Clin Oncol. 2021;18(1):9‐34. 32710082 10.1038/s41571-020-0403-1 PMC9721352

[317] Fang Y , Chen Y , Yu L , et al. Inhibition of breast cancer metastases by a novel inhibitor of TGFbeta receptor 1. J Natl Cancer Inst. 2013;105(1):47‐58. 23178439 10.1093/jnci/djs485

[318] Fuxe J , Vincent T , Garcia de Herreros A . Transcriptional crosstalk between TGF‐beta and stem cell pathways in tumor cell invasion: role of EMT promoting Smad complexes. Cell Cycle. 2010;9(12):2363‐2374. 20519943 10.4161/cc.9.12.12050

[319] Yuan JH , Yang F , Wang F , et al. A long noncoding RNA activated by TGF‐beta promotes the invasion‐metastasis cascade in hepatocellular carcinoma. Cancer Cell. 2014;25(5):666‐681. 24768205 10.1016/j.ccr.2014.03.010

[320] Wang L , Tong X , Zhou Z , et al. Circular RNA hsa_circ_0008305 (circPTK2) inhibits TGF‐beta‐induced epithelial‐mesenchymal transition and metastasis by controlling TIF1gamma in non‐small cell lung cancer. Mol Cancer. 2018;17(1):140. 30261900 10.1186/s12943-018-0889-7 PMC6161470

[321] Xu L , Liu W , Li T , et al. Long non‐coding RNA SMASR inhibits the EMT by negatively regulating TGF‐beta/Smad signaling pathway in lung cancer. Oncogene. 2021;40(20):3578‐3592. 33931741 10.1038/s41388-021-01760-2

[322] David CJ , Huang YH , Chen M , et al. TGF‐beta tumor suppression through a lethal EMT. Cell. 2016;164(5):1015‐1030. 26898331 10.1016/j.cell.2016.01.009 PMC4801341

[323] Nusse R , Clevers H . Wnt/beta‐catenin signaling, disease, and emerging therapeutic modalities. Cell. 2017;169(6):985‐999. 28575679 10.1016/j.cell.2017.05.016

[324] Angers S , Moon RT . Proximal events in Wnt signal transduction. Nat Rev Mol Cell Biol. 2009;10(7):468‐477. 19536106 10.1038/nrm2717

[325] Clevers H , Nusse R . Wnt/beta‐catenin signaling and disease. Cell. 2012;149(6):1192‐1205. 22682243 10.1016/j.cell.2012.05.012

[326] Reya T , Clevers H . Wnt signalling in stem cells and cancer. Nature. 2005;434(7035):843‐850. 15829953 10.1038/nature03319

[327] Harper KL , Sosa MS , Entenberg D , et al. Mechanism of early dissemination and metastasis in Her2(+) mammary cancer. Nature. 2016;540(7634):588‐592. 27974798 10.1038/nature20609 PMC5471138

[328] Sanchez‐Tillo E , de Barrios O , Siles L , Cuatrecasas M , Castells A , Postigo A . beta‐catenin/TCF4 complex induces the epithelial‐to‐mesenchymal transition (EMT)‐activator ZEB1 to regulate tumor invasiveness. Proc Natl Acad Sci USA. 2011;108(48):19204‐19209. 22080605 10.1073/pnas.1108977108 PMC3228467

[329] Xiong W , Zhang L , Liu H , et al. E2 ‐mediated EMT by activation of beta‐catenin/Snail signalling during the development of ovarian endometriosis. J Cell Mol Med. 2019;23(12):8035‐8045. 31560827 10.1111/jcmm.14668 PMC6850947

[330] Howe LR , Watanabe O , Leonard J , Brown AM . Twist is up‐regulated in response to Wnt1 and inhibits mouse mammary cell differentiation. Cancer Res. 2003;63(8):1906‐1913. 12702582

[331] Crawford HC , Fingleton B , Gustavson MD , et al. The PEA3 subfamily of Ets transcription factors synergizes with beta‐catenin‐LEF‐1 to activate matrilysin transcription in intestinal tumors. Mol Cell Biol. 2001;21(4):1370‐1383. 11158322 10.1128/MCB.21.4.1370-1383.2001 PMC99589

[332] Stemmer V , de Craene B , Berx G , Behrens J . Snail promotes Wnt target gene expression and interacts with beta‐catenin. Oncogene. 2008;27(37):5075‐5080. 18469861 10.1038/onc.2008.140

[333] Wu H , Lu XX , Wang JR , et al. TRAF6 inhibits colorectal cancer metastasis through regulating selective autophagic CTNNB1/beta‐catenin degradation and is targeted for GSK3B/GSK3beta‐mediated phosphorylation and degradation. Autophagy. 2019;15(9):1506‐1522. 30806153 10.1080/15548627.2019.1586250 PMC6693460

[334] Su J , Zhang A , Shi Z , et al. MicroRNA‐200a suppresses the Wnt/beta‐catenin signaling pathway by interacting with beta‐catenin. Int J Oncol. 2012;40(4):1162‐1170. 22211245 10.3892/ijo.2011.1322 PMC3584589

[335] Cong N , Du P , Zhang A , et al. Downregulated microRNA‐200a promotes EMT and tumor growth through the wnt/beta‐catenin pathway by targeting the E‐cadherin repressors ZEB1/ZEB2 in gastric adenocarcinoma. Oncol Rep. 2013;29(4):1579‐1587. 23381389 10.3892/or.2013.2267

[336] Shi J , Wang Y , Zeng L , et al. Disrupting the interaction of BRD4 with diacetylated Twist suppresses tumorigenesis in basal‐like breast cancer. Cancer Cell. 2014;25(2):210‐225. 24525235 10.1016/j.ccr.2014.01.028 PMC4004960

[337] Briscoe J , Therond PP . The mechanisms of Hedgehog signalling and its roles in development and disease. Nat Rev Mol Cell Biol. 2013;14(7):416‐429. 23719536 10.1038/nrm3598

[338] Ingham PW , Nakano Y , Seger C . Mechanisms and functions of Hedgehog signalling across the metazoa. Nat Rev Genet. 2011;12(6):393‐406. 21502959 10.1038/nrg2984

[339] Omenetti A , Bass LM , Anders RA , et al. Hedgehog activity, epithelial‐mesenchymal transitions, and biliary dysmorphogenesis in biliary atresia. Hepatology. 2011;53(4):1246‐1258. 21480329 10.1002/hep.24156 PMC3074103

[340] Syn WK , Jung Y , Omenetti A , et al. Hedgehog‐mediated epithelial‐to‐mesenchymal transition and fibrogenic repair in nonalcoholic fatty liver disease. Gastroenterology. 2009;137(4):1478‐1488. e8. 19577569 10.1053/j.gastro.2009.06.051 PMC2757536

[341] Rubin LL , de Sauvage FJ . Targeting the Hedgehog pathway in cancer. Nat Rev Drug Discov. 2006;5(12):1026‐1033. 17139287 10.1038/nrd2086

[342] Amakye D , Jagani Z , Dorsch M . Unraveling the therapeutic potential of the Hedgehog pathway in cancer. Nat Med. 2013;19(11):1410‐1422. 24202394 10.1038/nm.3389

[343] Chen X , Lingala S , Khoobyari S , Nolta J , Zern MA , Wu J . Epithelial mesenchymal transition and hedgehog signaling activation are associated with chemoresistance and invasion of hepatoma subpopulations. J Hepatol. 2011;55(4):838‐845. 21334406 10.1016/j.jhep.2010.12.043 PMC3177032

[344] El Khatib M , Kalnytska A , Palagani V , et al. Inhibition of hedgehog signaling attenuates carcinogenesis in vitro and increases necrosis of cholangiocellular carcinoma. Hepatology. 2013;57(3):1035‐1045. 23172661 10.1002/hep.26147

[345] Islam SS , Mokhtari RB , Noman AS , et al. Sonic hedgehog (Shh) signaling promotes tumorigenicity and stemness via activation of epithelial‐to‐mesenchymal transition (EMT) in bladder cancer. Mol Carcinog. 2016;55(5):537‐551. 25728352 10.1002/mc.22300

[346] Riaz SK , Ke Y , Wang F , Kayani MA , Malik MFA . Influence of SHH/GLI1 axis on EMT mediated migration and invasion of breast cancer cells. Sci Rep. 2019;9(1):6620. 31036836 10.1038/s41598-019-43093-x PMC6488587

[347] Ishii A , Shigemura K , Kitagawa K , et al. Anti‐tumor effect of hedgehog signaling inhibitor, vismodegib, on castration‐resistant prostate cancer. Anticancer Res. 2020;40(9):5107‐5114. 32878799 10.21873/anticanres.14514

[348] Qin T , Li B , Feng X , et al. Abnormally elevated USP37 expression in breast cancer stem cells regulates stemness, epithelial‐mesenchymal transition and cisplatin sensitivity. J Exp Clin Cancer Res. 2018;37(1):287. 30482232 10.1186/s13046-018-0934-9 PMC6258492

[349] Xiao Z , Chang L , Kim J , et al. USP37 is a SNAI1 deubiquitinase. Am J Cancer Res. 2019;9(12):2749‐2759. 31911859 PMC6943346

[350] Bhatia N , Thiyagarajan S , Elcheva I , et al. Gli2 is targeted for ubiquitination and degradation by beta‐TrCP ubiquitin ligase. J Biol Chem. 2006;281(28):19320‐19326. 16651270 10.1074/jbc.M513203200

[351] Sharma A , De R , Javed S , Srinivasan R , Pal A , Bhattacharyya S . Sonic hedgehog pathway activation regulates cervical cancer stem cell characteristics during epithelial to mesenchymal transition. J Cell Physiol. 2019. doi:10.1002/jcp.28231 10.1002/jcp.28231 30714153

[352] Neelakantan D , Zhou H , Oliphant MUJ , et al. EMT cells increase breast cancer metastasis via paracrine GLI activation in neighbouring tumour cells. Nat Commun. 2017;8:15773. 28604738 10.1038/ncomms15773 PMC5472791

[353] Joost S , Almada LL , Rohnalter V , et al. GLI1 inhibition promotes epithelial‐to‐mesenchymal transition in pancreatic cancer cells. Cancer Res. 2012;72(1):88‐99. 22086851 10.1158/0008-5472.CAN-10-4621 PMC3251693

[354] Zhao B , Li L , Lei Q , Guan KL . The Hippo‐YAP pathway in organ size control and tumorigenesis: an updated version. Genes Dev. 2010;24(9):862‐874. 20439427 10.1101/gad.1909210 PMC2861185

[355] Zhao B , Tumaneng K , Guan KL . The Hippo pathway in organ size control, tissue regeneration and stem cell self‐renewal. Nat Cell Biol. 2011;13(8):877‐883. 21808241 10.1038/ncb2303 PMC3987945

[356] Park J , Kim DH , Shah SR , et al. Switch‐like enhancement of epithelial‐mesenchymal transition by YAP through feedback regulation of WT1 and Rho‐family GTPases. Nat Commun. 2019;10(1):2797. 31243273 10.1038/s41467-019-10729-5 PMC6594963

[357] Liu SC , Hsu T , Chang YS , et al. Cytoplasmic LIF reprograms invasive mode to enhance NPC dissemination through modulating YAP1‐FAK/PXN signaling. Nat Commun. 2018;9(1):5105. 30504771 10.1038/s41467-018-07660-6 PMC6269507

[358] Lehmann W , Mossmann D , Kleemann J , et al. ZEB1 turns into a transcriptional activator by interacting with YAP1 in aggressive cancer types. Nat Commun. 2016;7:10498. 26876920 10.1038/ncomms10498 PMC4756710

[359] Feldker N , Ferrazzi F , Schuhwerk H , et al. Genome‐wide cooperation of EMT transcription factor ZEB1 with YAP and AP‐1 in breast cancer. EMBO J. 2020;39(17):e103209. 32692442 10.15252/embj.2019103209 PMC7459422

[360] Tang Y , Feinberg T , Keller ET , Li XY , Weiss SJ . Snail/Slug binding interactions with YAP/TAZ control skeletal stem cell self‐renewal and differentiation. Nat Cell Biol. 2016;18(9):917‐929. 27479603 10.1038/ncb3394 PMC5007193

[361] Tang Y , Weiss SJ . Snail/Slug‐YAP/TAZ complexes cooperatively regulate mesenchymal stem cell function and bone formation. Cell Cycle. 2017;16(5):399‐405. 28112996 10.1080/15384101.2017.1280643 PMC5351930

[362] Sun B , Zhong FJ , Xu C , et al. Programmed cell death 10 promotes metastasis and epithelial‐mesenchymal transition of hepatocellular carcinoma via PP2Ac‐mediated YAP activation. Cell Death Dis. 2021;12(9):849. 34521817 10.1038/s41419-021-04139-z PMC8440642

[363] Shi C , Cai Y , Li Y , et al. Yap promotes hepatocellular carcinoma metastasis and mobilization via governing cofilin/F‐actin/lamellipodium axis by regulation of JNK/Bnip3/SERCA/CaMKII pathways. Redox Biol. 2018;14:59‐71. 28869833 10.1016/j.redox.2017.08.013 PMC5582718

[364] Qiao Y , Chen J , Lim YB , et al. YAP regulates actin dynamics through ARHGAP29 and promotes metastasis. Cell Rep. 2017;19(8):1495‐1502. 28538170 10.1016/j.celrep.2017.04.075

[365] Jin D , Guo J , Wu Y , et al. m(6)A demethylase ALKBH5 inhibits tumor growth and metastasis by reducing YTHDFs‐mediated YAP expression and inhibiting miR‐107/LATS2‐mediated YAP activity in NSCLC. Mol Cancer. 2020;19(1):40. 32106857 10.1186/s12943-020-01161-1 PMC7045432

[366] Savorani C , Malinverno M , Seccia R , et al. A dual role of YAP in driving TGFbeta‐mediated endothelial‐to‐mesenchymal transition. J Cell Sci. 2021;134(15). 10.1242/jcs.251371 PMC8353525 34338295

[367] Quinn HM , Vogel R , Popp O , et al. YAP and beta‐catenin cooperate to drive oncogenesis in basal breast cancer. Cancer Res. 2021;81(8):2116‐2127. 33574090 10.1158/0008-5472.CAN-20-2801

[368] Liu Y , Wang G , Yang Y , et al. Increased TEAD4 expression and nuclear localization in colorectal cancer promote epithelial‐mesenchymal transition and metastasis in a YAP‐independent manner. Oncogene. 2016;35(21):2789‐2800. 26387538 10.1038/onc.2015.342

[369] Reuter S , Gupta SC , Chaturvedi MM , Aggarwal BB . Oxidative stress, inflammation, and cancer: how are they linked?. Free Radic Biol Med. 2010;49(11):1603‐16016. 20840865 10.1016/j.freeradbiomed.2010.09.006 PMC2990475

[370] Moloney JN , Cotter TG . ROS signalling in the biology of cancer. Semin Cell Dev Biol. 2018;80:50‐64. 28587975 10.1016/j.semcdb.2017.05.023

[371] Kumar B , Koul S , Khandrika L , Meacham RB , Koul HK . Oxidative stress is inherent in prostate cancer cells and is required for aggressive phenotype. Cancer Res. 2008;68(6):1777‐17785. 18339858 10.1158/0008-5472.CAN-07-5259

[372] Hayes JD , Dinkova‐Kostova AT , Tew KD . Oxidative stress in cancer. Cancer Cell. 2020;38(2):167‐197. 32649885 10.1016/j.ccell.2020.06.001 PMC7439808

[373] Ubellacker JM , Tasdogan A , Ramesh V , et al. Lymph protects metastasizing melanoma cells from ferroptosis. Nature. 2020;585(7823):113‐118. 32814895 10.1038/s41586-020-2623-z PMC7484468

[374] Wiel C , Le Gal K , Ibrahim MX , et al. BACH1 stabilization by antioxidants stimulates lung cancer metastasis. Cell. 2019;178(2):330‐345. e22. 31257027 10.1016/j.cell.2019.06.005

[375] Rojo de la Vega M , Chapman E , Zhang DD . NRF2 and the hallmarks of cancer. Cancer Cell. 2018;34(1):21‐43. 29731393 10.1016/j.ccell.2018.03.022 PMC6039250

[376] Morgan MJ , Liu ZG . Crosstalk of reactive oxygen species and NF‐kappaB signaling. Cell Res. 2011;21(1):103‐115. 21187859 10.1038/cr.2010.178 PMC3193400

[377] Sablina AA , Budanov AV , Ilyinskaya GV , Agapova LS , Kravchenko JE , Chumakov PM . The antioxidant function of the p53 tumor suppressor. Nat Med. 2005;11(12):1306‐1313. 16286925 10.1038/nm1320 PMC2637821

[378] Klotz LO , Sanchez‐Ramos C , Prieto‐Arroyo I , Urbanek P , Steinbrenner H , Monsalve M . Redox regulation of FoxO transcription factors. Redox Biol. 2015;6:51‐72. 26184557 10.1016/j.redox.2015.06.019 PMC4511623

[379] Griess B , Tom E , Domann F , Teoh‐Fitzgerald M . Extracellular superoxide dismutase and its role in cancer. Free Radic Biol Med. 2017;112:464‐479. 28842347 10.1016/j.freeradbiomed.2017.08.013 PMC5685559

[380] Galasso M , Gambino S , Romanelli MG , Donadelli M , Scupoli MT . Browsing the oldest antioxidant enzyme: catalase and its multiple regulation in cancer. Free Radic Biol Med. 2021;172:264‐272. 34129927 10.1016/j.freeradbiomed.2021.06.010

[381] Hampton MB , Vick KA , Skoko JJ , Neumann CA . Peroxiredoxin involvement in the initiation and progression of human cancer. Antioxid Redox Signal. 2018;28(7):591‐608. 29237274 10.1089/ars.2017.7422 PMC9836708

[382] Zhang J , Li X , Han X , Liu R , Fang J . Targeting the thioredoxin system for cancer therapy. Trends Pharmacol Sci. 2017;38(9):794‐808. 28648527 10.1016/j.tips.2017.06.001

[383] Brigelius‐Flohe R , Flohe L . Regulatory phenomena in the glutathione peroxidase superfamily. Antioxid Redox Signal. 2020;33(7):498‐516. 31822117 10.1089/ars.2019.7905

[384] Bansal A , Simon MC . Glutathione metabolism in cancer progression and treatment resistance. J Cell Biol. 2018;217(7):2291‐2298. 29915025 10.1083/jcb.201804161 PMC6028537

[385] Cimmino L , Neel BG , Aifantis I . Vitamin C in stem cell reprogramming and cancer. Trends Cell Biol. 2018;28(9):698‐708. 29724526 10.1016/j.tcb.2018.04.001 PMC6102081

[386] Jiang Q . Natural forms of vitamin E: metabolism, antioxidant, and anti‐inflammatory activities and their role in disease prevention and therapy. Free Radic Biol Med. 2014;72:76‐90. 24704972 10.1016/j.freeradbiomed.2014.03.035 PMC4120831

[387] Gorrini C , Harris IS , Mak TW . Modulation of oxidative stress as an anticancer strategy. Nat Rev Drug Discov. 2013;12(12):931‐947. 24287781 10.1038/nrd4002

[388] Glasauer A , Chandel NS . Targeting antioxidants for cancer therapy. Biochem Pharmacol. 2014;92(1):90‐101. 25078786 10.1016/j.bcp.2014.07.017

[389] Poprac P , Jomova K , Simunkova M , Kollar V , Rhodes CJ , Valko M . Targeting free radicals in oxidative stress‐related human diseases. Trends Pharmacol Sci. 2017;38(7):592‐607. 28551354 10.1016/j.tips.2017.04.005

[390] Sun L , Dong H , Zhang W , et al. Lipid peroxidation, GSH depletion, and SLC7A11 inhibition are common causes of EMT and ferroptosis in A549 cells, but different in specific mechanisms. DNA Cell Biol. 2021;40(2):172‐183. 33351681 10.1089/dna.2020.5730

[391] Haley JA , Ruiz CF , Montal ED , Wang D , Haley JD , Girnun GD . Decoupling of Nrf2 expression promotes mesenchymal state maintenance in non‐small cell lung cancer. Cancers (Basel). 2019;11(10). 10.3390/cancers11101488 PMC6826656 31581742

[392] Kong D , Zhang Z , Chen L , et al. Curcumin blunts epithelial‐mesenchymal transition of hepatocytes to alleviate hepatic fibrosis through regulating oxidative stress and autophagy. Redox Biol. 2020;36:101600. 32526690 10.1016/j.redox.2020.101600 PMC7287144

[393] Xu J , Liu D , Niu H , et al. Resveratrol reverses doxorubicin resistance by inhibiting epithelial‐mesenchymal transition (EMT) through modulating PTEN/Akt signaling pathway in gastric cancer. J Exp Clin Cancer Res. 2017;36(1):19. 28126034 10.1186/s13046-016-0487-8 PMC5270306

[394] Luo M , Shang L , Brooks MD , et al. Targeting breast cancer stem cell state equilibrium through modulation of redox signaling. Cell Metab. 2018;28(1):69‐86. e6. 29972798 10.1016/j.cmet.2018.06.006 PMC6037414

[395] Watson J . Oxidants, antioxidants and the current incurability of metastatic cancers. Open Biol. 2013;3(1):120144. 23303309 10.1098/rsob.120144 PMC3603456

[396] Wang Y , Yang J , Yi J . Redox sensing by proteins: oxidative modifications on cysteines and the consequent events. Antioxid Redox Signal. 2012;16(7):649‐657. 21967570 10.1089/ars.2011.4313

[397] Lan J , Huang Z , Han J , Shao J , Huang C . Redox regulation of microRNAs in cancer. Cancer Lett. 2018;418:250‐259. 29330105 10.1016/j.canlet.2018.01.010

[398] Forman HJ . Redox signaling: an evolution from free radicals to aging. Free Radic Biol Med. 2016;97:398‐407. 27393004 10.1016/j.freeradbiomed.2016.07.003 PMC4996735

[399] Anastasiou D , Poulogiannis G , Asara JM , et al. Inhibition of pyruvate kinase M2 by reactive oxygen species contributes to cellular antioxidant responses. Science. 2011;334(6060):1278‐1283. 22052977 10.1126/science.1211485 PMC3471535

[400] Giannoni E , Parri M , Chiarugi P . EMT and oxidative stress: a bidirectional interplay affecting tumor malignancy. Antioxid Redox Signal. 2012;16(11):1248‐1263. 21929373 10.1089/ars.2011.4280

[401] Yang IH , Lee JJ , Wu PC , Kuo HK , Kuo YH , Huang HM . Oxidative stress enhanced the transforming growth factor‐beta2‐induced epithelial‐mesenchymal transition through chemokine ligand 1 on ARPE‐19 cell. Sci Rep. 2020;10(1):4000. 32132577 10.1038/s41598-020-60785-x PMC7055234

[402] Shi Y , Wang S , Yang R , et al. ros promote hypoxia‐induced keratinocyte epithelial‐mesenchymal transition by inducing SOX2 expression and subsequent activation of Wnt/beta‐catenin. Oxid Med Cell Longev. 2022;2022:1084006. 35035654 10.1155/2022/1084006 PMC8758332

[403] Gandhirajan RK , Jain M , Walla B , et al. Cysteine S‐glutathionylation promotes stability and activation of the hippo downstream effector transcriptional co‐activator with PDZ‐binding Motif (TAZ). J Biol Chem. 2016;291(22):11596‐11607. 27048650 10.1074/jbc.M115.712539 PMC4882430

[404] Usatyuk PV , Parinandi NL , Natarajan V . Redox regulation of 4‐hydroxy‐2‐nonenal‐mediated endothelial barrier dysfunction by focal adhesion, adherens, and tight junction proteins. J Biol Chem. 2006;281(46):35554‐35566. 16982627 10.1074/jbc.M607305200

[405] Chattopadhyay R , Raghavan S , Rao GN . Resolvin D1 via prevention of ROS‐mediated SHP2 inactivation protects endothelial adherens junction integrity and barrier function. Redox Biol. 2017;12:438‐455. 28319894 10.1016/j.redox.2017.02.023 PMC5357675

[406] Han J , Weisbrod RM , Shao D , et al. The redox mechanism for vascular barrier dysfunction associated with metabolic disorders: glutathionylation of Rac1 in endothelial cells. Redox Biol. 2016;9:306‐319. 27693992 10.1016/j.redox.2016.09.003 PMC5045950

[407] Garcia‐Ortiz A , Martin‐Cofreces NB , Ibiza S , et al. eNOS S‐nitrosylates beta‐actin on Cys374 and regulates PKC‐theta at the immune synapse by impairing actin binding to profilin‐1. PLoS Biol. 2017;15(4):e2000653. 28394935 10.1371/journal.pbio.2000653 PMC5386235

[408] Kawai N , Tsuji S , Tsujii M , et al. Tumor necrosis factor alpha stimulates invasion of Src‐activated intestinal cells. Gastroenterology. 2002;122(2):331‐339. 11832448 10.1053/gast.2002.31023

[409] Yamamoto N , Kan OK , Tatsuta M , et al. Incense smoke‐induced oxidative stress disrupts tight junctions and bronchial epithelial barrier integrity and induces airway hyperresponsiveness in mouse lungs. Sci Rep. 2021;11(1):7222. 33790367 10.1038/s41598-021-86745-7 PMC8012366

[410] Chen L , Zhu Y , Zhou J , et al. Luteolin alleviates epithelial‐mesenchymal transformation induced by oxidative injury in ARPE‐19 cell via Nrf2 and AKT/GSK‐3beta pathway. Oxid Med Cell Longev. 2022;2022:2265725. 35198094 10.1155/2022/2265725 PMC8860553

[411] Abate C , Patel L , Rauscher FJ 3rd , Curran T . Redox regulation of fos and jun DNA‐binding activity in vitro. Science. 1990;249(4973):1157‐1161. 2118682 10.1126/science.2118682

[412] Bannister AJ , Cook A , Kouzarides T . In vitro DNA binding activity of Fos/Jun and BZLF1 but not C/EBP is affected by redox changes. Oncogene. 1991;6(7):1243‐1250. 1907361

[413] Yin Z , Machius M , Nestler EJ , Rudenko G . Activator Protein‐1: redox switch controlling structure and DNA‐binding. Nucleic Acids Res. 2017;45(19):11425‐11436. 28981703 10.1093/nar/gkx795 PMC5737521

[414] Li Y , Liu Y , Xu Y , Voorhees JJ , Fisher GJ . UV irradiation induces Snail expression by AP‐1 dependent mechanism in human skin keratinocytes. J Dermatol Sci. 2010;60(2):105‐113. 20851575 10.1016/j.jdermsci.2010.08.003

[415] Lin CS , Lin CL , Ying TH , et al. beta‐Mangostin inhibits the metastatic power of cervical cancer cells attributing to suppression of JNK2/AP‐1/Snail cascade. J Cell Physiol. 2020;235(11):8446‐8460. 32324277 10.1002/jcp.29688

[416] Bakiri L , Macho‐Maschler S , Custic I , et al. Fra‐1/AP‐1 induces EMT in mammary epithelial cells by modulating Zeb1/2 and TGFbeta expression. Cell Death Differ. 2015;22(2):336‐350. 25301070 10.1038/cdd.2014.157 PMC4291495

[417] Zhao C , Qiao Y , Jonsson P , et al. Genome‐wide profiling of AP‐1‐regulated transcription provides insights into the invasiveness of triple‐negative breast cancer. Cancer Res. 2014;74(14):3983‐3994. 24830720 10.1158/0008-5472.CAN-13-3396

[418] Dong Q , Zhou C , Ren H , et al. Lactate‐induced MRP1 expression contributes to metabolism‐based etoposide resistance in non‐small cell lung cancer cells. Cell Commun Signal. 2020;18(1):167. 33097055 10.1186/s12964-020-00653-3 PMC7583203

[419] Nam EH , Lee Y , Moon B , Lee JW , Kim S . Twist1 and AP‐1 cooperatively upregulate integrin alpha5 expression to induce invasion and the epithelial‐mesenchymal transition. Carcinogenesis. 2015;36(3):327‐337. 25600770 10.1093/carcin/bgv005

[420] Briggs KJ , Koivunen P , Cao S , et al. Paracrine induction of HIF by glutamate in breast cancer: eglN1 senses cysteine. Cell. 2016;166(1):126‐139. 27368101 10.1016/j.cell.2016.05.042 PMC4930557

[421] Zhang W , Shi X , Peng Y , et al. HIF‐1alpha promotes epithelial‐mesenchymal transition and metastasis through direct regulation of ZEB1 in colorectal cancer. PLoS One. 2015;10(6):e0129603. 26057751 10.1371/journal.pone.0129603 PMC4461314

[422] Joseph JV , Conroy S , Pavlov K , et al. Hypoxia enhances migration and invasion in glioblastoma by promoting a mesenchymal shift mediated by the HIF1alpha‐ZEB1 axis. Cancer Lett. 2015;359(1):107‐116. 25592037 10.1016/j.canlet.2015.01.010

[423] Yang MH , Wu MZ , Chiou SH , et al. Direct regulation of TWIST by HIF‐1alpha promotes metastasis. Nat Cell Biol. 2008;10(3):295‐305. 18297062 10.1038/ncb1691

[424] Lv L , Yuan J , Huang T , et al. Stabilization of Snail by HIF‐1alpha and TNF‐alpha is required for hypoxia‐induced invasion in prostate cancer PC3 cells. Mol Biol Rep. 2014;41(7):4573‐4582. 24610352 10.1007/s11033-014-3328-x

[425] Herscovitch M , Comb W , Ennis T , et al. Intermolecular disulfide bond formation in the NEMO dimer requires Cys54 and Cys347. Biochem Biophys Res Commun. 2008;367(1):103‐108. 18164680 10.1016/j.bbrc.2007.12.123 PMC2277332

[426] Min C , Eddy SF , Sherr DH , Sonenshein GE . NF‐kappaB and epithelial to mesenchymal transition of cancer. J Cell Biochem. 2008;104(3):733‐744. 18253935 10.1002/jcb.21695

[427] de Keizer PL , Burgering BM , Dansen TB . Forkhead box o as a sensor, mediator, and regulator of redox signaling. Antioxid Redox Signal. 2011;14(6):1093‐1106. 20626320 10.1089/ars.2010.3403

[428] Putker M , Madl T , Vos HR , et al. Redox‐dependent control of FOXO/DAF‐16 by transportin‐1. Mol Cell. 2013;49(4):730‐742. 23333309 10.1016/j.molcel.2012.12.014

[429] Jiramongkol Y , Lam EW . FOXO transcription factor family in cancer and metastasis. Cancer Metastasis Rev. 2020;39(3):681‐709. 32372224 10.1007/s10555-020-09883-w PMC7497309

[430] Wu JB , Shao C , Li X , et al. Monoamine oxidase A mediates prostate tumorigenesis and cancer metastasis. J Clin Invest. 2014;124(7):2891‐2908. 24865426 10.1172/JCI70982 PMC4071401

[431] Shin S , Buel GR , Nagiec MJ , et al. ERK2 regulates epithelial‐to‐mesenchymal plasticity through DOCK10‐dependent Rac1/FoxO1 activation. Proc Natl Acad Sci USA. 2019;116(8):2967‐2976. 30728292 10.1073/pnas.1811923116 PMC6386703

[432] Liu H , Yin J , Wang H , et al. FOXO3a modulates WNT/beta‐catenin signaling and suppresses epithelial‐to‐mesenchymal transition in prostate cancer cells. Cell Signal. 2015;27(3):510‐518. 25578861 10.1016/j.cellsig.2015.01.001

[433] Yang J , Li T , Gao C , et al. FOXO1 3'UTR functions as a ceRNA in repressing the metastases of breast cancer cells via regulating miRNA activity. FEBS Lett. 2014;588(17):3218‐3224. 25017439 10.1016/j.febslet.2014.07.003

[434] Hanahan D , Weinberg RA . Hallmarks of cancer: the next generation. Cell. 2011;144(5):646‐674. 21376230 10.1016/j.cell.2011.02.013

[435] Lunt SY , Vander Heiden MG . Aerobic glycolysis: meeting the metabolic requirements of cell proliferation. Annu Rev Cell Dev Biol. 2011;27:441‐464. 21985671 10.1146/annurev-cellbio-092910-154237

[436] Kim NH , Cha YH , Lee J , et al. Snail reprograms glucose metabolism by repressing phosphofructokinase PFKP allowing cancer cell survival under metabolic stress. Nat Commun. 2017;8:14374. 28176759 10.1038/ncomms14374 PMC5309788

[437] Dong C , Yuan T , Wu Y , et al. Loss of FBP1 by Snail‐mediated repression provides metabolic advantages in basal‐like breast cancer. Cancer Cell. 2013;23(3):316‐331. 23453623 10.1016/j.ccr.2013.01.022 PMC3703516

[438] Schafer ZT , Grassian AR , Song L , et al. Antioxidant and oncogene rescue of metabolic defects caused by loss of matrix attachment. Nature. 2009;461(7260):109‐113. 19693011 10.1038/nature08268 PMC2931797

[439] Cha YH , Yook JI , Kim HS , Kim NH . Catabolic metabolism during cancer EMT. Arch Pharm Res. 2015;38(3):313‐320. 25634102 10.1007/s12272-015-0567-x

[440] Liang C , Shi S , Qin Y , et al. Localisation of PGK1 determines metabolic phenotype to balance metastasis and proliferation in patients with SMAD4‐negative pancreatic cancer. Gut. 2020;69(5):888‐900. 31611300 10.1136/gutjnl-2018-317163

[441] Park SY , Kim HS , Kim NH , et al. Snail1 is stabilized by O‐GlcNAc modification in hyperglycaemic condition. EMBO J. 2010;29(22):3787‐3796. 20959806 10.1038/emboj.2010.254 PMC2989108

[442] Lee CK , Jeong SH , Jang C , et al. Tumor metastasis to lymph nodes requires YAP‐dependent metabolic adaptation. Science. 2019;363(6427):644‐649. 30733421 10.1126/science.aav0173

[443] Corbet C , Bastien E , Santiago de Jesus JP , et al. TGFbeta2‐induced formation of lipid droplets supports acidosis‐driven EMT and the metastatic spreading of cancer cells. Nat Commun. 2020;11(1):454. 31974393 10.1038/s41467-019-14262-3 PMC6978517

[444] Nath A , Li I , Roberts LR , Chan C . Elevated free fatty acid uptake via CD36 promotes epithelial‐mesenchymal transition in hepatocellular carcinoma. Sci Rep. 2015;5:14752. 26424075 10.1038/srep14752 PMC4589791

[445] Bergers G , Fendt SM . The metabolism of cancer cells during metastasis. Nat Rev Cancer. 2021;21(3):162‐180. 33462499 10.1038/s41568-020-00320-2 PMC8733955

[446] Liu Y , Lu LL , Wen D , et al. MiR‐612 regulates invadopodia of hepatocellular carcinoma by HADHA‐mediated lipid reprogramming. J Hematol Oncol. 2020;13(1):12. 32033570 10.1186/s13045-019-0841-3 PMC7006096

[447] Wen J , Min X , Shen M , et al. ACLY facilitates colon cancer cell metastasis by CTNNB1. J Exp Clin Cancer Res. 2019;38(1):401. 31511060 10.1186/s13046-019-1391-9 PMC6740040

[448] Liu D , Zhang T , Chen X , et al. ONECUT2 facilitates hepatocellular carcinoma metastasis by transcriptionally upregulating FGF2 and ACLY. Cell Death Dis. 2021;12(12):1113. 34839358 10.1038/s41419-021-04410-3 PMC8627506

[449] Doll S , Proneth B , Tyurina YY , et al. ACSL4 dictates ferroptosis sensitivity by shaping cellular lipid composition. Nat Chem Biol. 2017;13(1):91‐98. 27842070 10.1038/nchembio.2239 PMC5610546

[450] Jin H , He Y , Zhao P , et al. Targeting lipid metabolism to overcome EMT‐associated drug resistance via integrin beta3/FAK pathway and tumor‐associated macrophage repolarization using legumain‐activatable delivery. Theranostics. 2019;9(1):265‐278. 30662566 10.7150/thno.27246 PMC6332796

[451] Beck B , Lapouge G , Rorive S , et al. Different levels of Twist1 regulate skin tumor initiation, stemness, and progression. Cell Stem Cell. 2015;16(1):67‐79. 25575080 10.1016/j.stem.2014.12.002

[452] Siebzehnrubl FA , Silver DJ , Tugertimur B , et al. The ZEB1 pathway links glioblastoma initiation, invasion and chemoresistance. EMBO Mol Med. 2013;5(8):1196‐1212. 23818228 10.1002/emmm.201302827 PMC3944461

[453] Song SJ , Poliseno L , Song MS , et al. MicroRNA‐antagonism regulates breast cancer stemness and metastasis via TET‐family‐dependent chromatin remodeling. Cell. 2013;154(2):311‐324. 23830207 10.1016/j.cell.2013.06.026 PMC3767157

[454] Lopez‐Menendez C , Vazquez‐Naharro A , Santos V , et al. E2A modulates stemness, metastasis, and therapeutic resistance of breast cancer. Cancer Res. 2021;81(17):4529‐4544. 34145034 10.1158/0008-5472.CAN-20-2685 PMC7611611

[455] Sayan AE , Griffiths TR , Pal R , et al. SIP1 protein protects cells from DNA damage‐induced apoptosis and has independent prognostic value in bladder cancer. Proc Natl Acad Sci USA. 2009;106(35):14884‐14889. 19706487 10.1073/pnas.0902042106 PMC2736415

[456] Zhou S , Schuetz JD , Bunting KD , et al. The ABC transporter Bcrp1/ABCG2 is expressed in a wide variety of stem cells and is a molecular determinant of the side‐population phenotype. Nat Med. 2001;7(9):1028‐1034. 11533706 10.1038/nm0901-1028

[457] Liu M , Zhang Y , Yang J , et al. ZIP4 increases expression of transcription factor ZEB1 to promote integrin alpha3beta1 signaling and inhibit expression of the gemcitabine transporter ENT1 in pancreatic cancer cells. Gastroenterology. 2020;158(3):679‐692. e1. 31711924 10.1053/j.gastro.2019.10.038 PMC7837454

[458] Lionarons DA , Hancock DC , Rana S , et al. RAC1(P29S) induces a mesenchymal phenotypic switch via serum response factor to promote melanoma development and therapy resistance. Cancer Cell. 2019;36(1):68‐83. e9. 31257073 10.1016/j.ccell.2019.05.015 PMC6617390

[459] Shen S , Vagner S , Robert C . Persistent cancer cells: the deadly survivors. Cell. 2020;183(4):860‐874. 33186528 10.1016/j.cell.2020.10.027

[460] Recasens A , Munoz L . Targeting cancer cell dormancy. Trends Pharmacol Sci. 2019;40(2):128‐141. 30612715 10.1016/j.tips.2018.12.004

[461] Hangauer MJ , Viswanathan VS , Ryan MJ , et al. Drug‐tolerant persister cancer cells are vulnerable to GPX4 inhibition. Nature. 2017;551(7679):247‐250. 29088702 10.1038/nature24297 PMC5933935

[462] Zhang Z , Qin S , Chen Y , et al. Inhibition of NPC1L1 disrupts adaptive responses of drug‐tolerant persister cells to chemotherapy. EMBO Mol Med. 2022;14(2):e14903. 35023619 10.15252/emmm.202114903 PMC8819355

[463] Creighton CJ , Li X , Landis M , et al. Residual breast cancers after conventional therapy display mesenchymal as well as tumor‐initiating features. Proc Natl Acad Sci USA. 2009;106(33):13820‐13825. 19666588 10.1073/pnas.0905718106 PMC2720409

[464] Rhim AD , Mirek ET , Aiello NM , et al. EMT and dissemination precede pancreatic tumor formation. Cell. 2012;148(1‐2):349‐361. 22265420 10.1016/j.cell.2011.11.025 PMC3266542

[465] Perusina Lanfranca M , Zhang Y , Girgis A , et al. Interleukin 22 signaling regulates acinar cell plasticity to promote pancreatic tumor development in mice. Gastroenterology. 2020;158(5):1417‐1432. e11. 31843590 10.1053/j.gastro.2019.12.010 PMC7197347

[466] Mitra A , Yan J , Xia X , et al. IL6‐mediated inflammatory loop reprograms normal to epithelial‐mesenchymal transition(+) metastatic cancer stem cells in preneoplastic liver of transforming growth factor beta‐deficient beta2‐spectrin(+/‐) mice. Hepatology. 2017;65(4):1222‐1236. 27863449 10.1002/hep.28951 PMC5360560

[467] Lee CC , Lin JC , Hwang WL , et al. Macrophage‐secreted interleukin‐35 regulates cancer cell plasticity to facilitate metastatic colonization. Nat Commun. 2018;9(1):3763. 30218063 10.1038/s41467-018-06268-0 PMC6138674

[468] Yeung OW , Lo CM , Ling CC , et al. Alternatively activated (M2) macrophages promote tumour growth and invasiveness in hepatocellular carcinoma. J Hepatol. 2015;62(3):607‐616. 25450711 10.1016/j.jhep.2014.10.029

[469] Chen Y , Hao X , Sun R , Wei H , Tian Z . Natural killer cell‐derived interferon‐gamma promotes hepatocellular carcinoma through the epithelial cell adhesion molecule‐epithelial‐to‐mesenchymal transition axis in Hepatitis B Virus Transgenic Mice. Hepatology. 2019;69(4):1735‐1750. 30329167 10.1002/hep.30317

[470] Dongre A , Rashidian M , Reinhardt F , et al. Epithelial‐to‐mesenchymal transition contributes to immunosuppression in breast carcinomas. Cancer Res. 2017;77(15):3982‐3989. 28428275 10.1158/0008-5472.CAN-16-3292 PMC5541771

[471] Su S , Liu Q , Chen J , et al. A positive feedback loop between mesenchymal‐like cancer cells and macrophages is essential to breast cancer metastasis. Cancer Cell. 2014;25(5):605‐620. 24823638 10.1016/j.ccr.2014.03.021

[472] Chockley PJ , Chen J , Chen G , Beer DG , Standiford TJ , Keshamouni VG . Epithelial‐mesenchymal transition leads to NK cell‐mediated metastasis‐specific immunosurveillance in lung cancer. J Clin Invest. 2018;128(4):1384‐1396. 29324443 10.1172/JCI97611 PMC5873856

[473] Grigore AD , Jolly MK , Jia D , Farach‐Carson MC , Levine H . Tumor budding: the name is EMT. Partial EMT. J Clin Med. 2016;5(5). 10.3390/jcm5050051 PMC4882480 27136592

[474] Navas T , Kinders RJ , Lawrence SM , et al. Clinical evolution of epithelial‐mesenchymal transition in human carcinomas. Cancer Res. 2020;80(2):304‐318. 31732654 10.1158/0008-5472.CAN-18-3539 PMC8170833

[475] Hur K , Toiyama Y , Takahashi M , et al. MicroRNA‐200c modulates epithelial‐to‐mesenchymal transition (EMT) in human colorectal cancer metastasis. Gut. 2013;62(9):1315‐1326. 22735571 10.1136/gutjnl-2011-301846 PMC3787864

[476] Roseweir AK , Kong CY , Park JH , et al. A novel tumor‐based epithelial‐to‐mesenchymal transition score that associates with prognosis and metastasis in patients with Stage II/III colorectal cancer. Int J Cancer. 2019;144(1):150‐159. 29992570 10.1002/ijc.31739

[477] Knudsen ES , Ertel A , Davicioni E , Kline J , Schwartz GF , Witkiewicz AK . Progression of ductal carcinoma in situ to invasive breast cancer is associated with gene expression programs of EMT and myoepithelia. Breast Cancer Res Treat. 2012;133(3):1009‐1024. 22134623 10.1007/s10549-011-1894-3

[478] Stark TW , Hensley PJ , Spear A , Pu H , Strup SS , Kyprianou N . Predictive value of epithelial‐mesenchymal‐transition (EMT) signature and PARP‐1 in prostate cancer radioresistance. Prostate. 2017;77(16):1583‐1591. 29063620 10.1002/pros.23435

[479] Aktas B , Tewes M , Fehm T , Hauch S , Kimmig R , Kasimir‐Bauer S . Stem cell and epithelial‐mesenchymal transition markers are frequently overexpressed in circulating tumor cells of metastatic breast cancer patients. Breast Cancer Res. 2009;11(4):R46. 19589136 10.1186/bcr2333 PMC2750105

[480] Ning Y , Zhang W , Hanna DL , et al. Clinical relevance of EMT and stem‐like gene expression in circulating tumor cells of metastatic colorectal cancer patients. Pharmacogenomics J. 2018;18(1):29‐34. 27503579 10.1038/tpj.2016.62 PMC7505126

[481] Song B , Park SH , Zhao JC , et al. Targeting FOXA1‐mediated repression of TGF‐beta signaling suppresses castration‐resistant prostate cancer progression. J Clin Invest. 2019;129(2):569‐582. 30511964 10.1172/JCI122367 PMC6355239

[482] Sim WJ , Iyengar PV , Lama D , et al. c‐Met activation leads to the establishment of a TGFbeta‐receptor regulatory network in bladder cancer progression. Nat Commun. 2019;10(1): 4349. 31554791 10.1038/s41467-019-12241-2 PMC6761206

[483] Giannelli G , Villa E , Lahn M . Transforming growth factor‐beta as a therapeutic target in hepatocellular carcinoma. Cancer Res. 2014;74(7):1890‐1894. 24638984 10.1158/0008-5472.CAN-14-0243

[484] Melisi D , Garcia‐Carbonero R , Macarulla T , et al. TGFbeta receptor inhibitor galunisertib is linked to inflammation‐ and remodeling‐related proteins in patients with pancreatic cancer. Cancer Chemother Pharmacol. 2019;83(5):975‐991. 30887178 10.1007/s00280-019-03807-4

[485] Kelley RK , Gane E , Assenat E , et al. A phase 2 study of galunisertib (TGF‐beta1 Receptor Type I Inhibitor) and sorafenib in patients with advanced hepatocellular carcinoma. Clin Transl Gastroenterol. 2019;10(7):e00056. 31295152 10.14309/ctg.0000000000000056 PMC6708671

[486] Kothari AN , Mi Z , Zapf M , Kuo PC . Novel clinical therapeutics targeting the epithelial to mesenchymal transition. Clin Transl Med. 2014;3:35. 25343018 10.1186/s40169-014-0035-0 PMC4198571

[487] Uckun FM , Qazi S , Hwang L , Trieu VN . Recurrent or refractory high‐grade gliomas treated by convection‐enhanced delivery of a TGFbeta2‐Targeting RNA therapeutic: a post‐hoc analysis with long‐term follow‐up. Cancers (Basel). 2019;11(12). 10.3390/cancers11121892 PMC6966490 31795071

[488] Birch JL , Coull BJ , Spender LC , et al. Multifaceted transforming growth factor‐beta (TGFbeta) signalling in glioblastoma. Cell Signal. 2020;72:109638. 32320860 10.1016/j.cellsig.2020.109638

[489] Haider C , Hnat J , Wagner R , et al. Transforming growth factor‐beta and Axl induce CXCL5 and neutrophil recruitment in hepatocellular carcinoma. Hepatology. 2019;69(1):222‐236. 30014484 10.1002/hep.30166 PMC6590451

[490] Gherardi E , Birchmeier W , Birchmeier C , Vande Woude G . Targeting MET in cancer: rationale and progress. Nat Rev Cancer. 2012;12(2):89‐103. 22270953 10.1038/nrc3205

[491] Wolf J , Seto T , Han JY , et al. Capmatinib in MET exon 14‐mutated or MET‐amplified non‐small‐cell lung cancer. N Engl J Med. 2020;383(10):944‐957. 32877583 10.1056/NEJMoa2002787

[492] Guo R , Luo J , Chang J , Rekhtman N , Arcila M , Drilon A . MET‐dependent solid tumours ‐ molecular diagnosis and targeted therapy. Nat Rev Clin Oncol. 2020;17(9):569‐587. 32514147 10.1038/s41571-020-0377-z PMC7478851

[493] Wu YL , Cheng Y , Zhou J , et al. Tepotinib plus gefitinib in patients with EGFR‐mutant non‐small‐cell lung cancer with MET overexpression or MET amplification and acquired resistance to previous EGFR inhibitor (INSIGHT study): an open‐label, phase 1b/2, multicentre, randomised trial. Lancet Respir Med. 2020;8(11):1132‐1143. 32479794 10.1016/S2213-2600(20)30154-5

[494] Ryoo BY , Cheng AL , Ren Z , et al. Randomised Phase 1b/2 trial of tepotinib vs sorafenib in Asian patients with advanced hepatocellular carcinoma with MET overexpression. Br J Cancer. 2021;125(2):200‐208. 33972742 10.1038/s41416-021-01380-3 PMC8292411

[495] Bocca C , Bozzo F , Cannito S , Parola M , Miglietta A . Celecoxib inactivates epithelial‐mesenchymal transition stimulated by hypoxia and/or epidermal growth factor in colon cancer cells. Mol Carcinog. 2012;51(10):783‐795. 21882253 10.1002/mc.20846

[496] Chiang SL , Velmurugan BK , Chung CM , et al. Preventive effect of celecoxib use against cancer progression and occurrence of oral squamous cell carcinoma. Sci Rep. 2017;7(1):6235. 28740192 10.1038/s41598-017-06673-3 PMC5524966

[497] Zhou L , Liu XD , Sun M , et al. Targeting MET and AXL overcomes resistance to sunitinib therapy in renal cell carcinoma. Oncogene. 2016;35(21):2687‐2697. 26364599 10.1038/onc.2015.343 PMC4791213

[498] Namba K , Shien K , Takahashi Y , et al. Activation of AXL as a preclinical acquired resistance mechanism against osimertinib treatment in EGFR‐mutant non‐small cell lung cancer cells. Mol Cancer Res. 2019;17(2):499‐507. 30463991 10.1158/1541-7786.MCR-18-0628

[499] Pal SK , McGregor B , Suarez C , et al. Cabozantinib in combination with atezolizumab for advanced renal cell carcinoma: results from the COSMIC‐021 study. J Clin Oncol. 2021;39(33):3725‐3736. 34491815 10.1200/JCO.21.00939 PMC8601305

[500] Augustine CK , Yoshimoto Y , Gupta M , et al. Targeting N‐cadherin enhances antitumor activity of cytotoxic therapies in melanoma treatment. Cancer Res. 2008;68(10):3777‐3784. 18483261 10.1158/0008-5472.CAN-07-5949

[501] Bargagna‐Mohan P , Hamza A , Kim YE , et al. The tumor inhibitor and antiangiogenic agent withaferin A targets the intermediate filament protein vimentin. Chem Biol. 2007;14(6):623‐634. 17584610 10.1016/j.chembiol.2007.04.010 PMC3228641

[502] Thaiparambil JT , Bender L , Ganesh T , et al. Withaferin A inhibits breast cancer invasion and metastasis at sub‐cytotoxic doses by inducing vimentin disassembly and serine 56 phosphorylation. Int J Cancer. 2011;129(11):2744‐2755.2153835010.1002/ijc.25938

[503] Glassy MC , Hagiwara H . Summary analysis of the pre‐clinical and clinical results of brain tumor patients treated with pritumumab. Hum Antibodies. 2009;18(4):127‐137. 19996527 10.3233/HAB-2009-0209

[504] Peng DH , Kundu ST , Fradette JJ , et al. ZEB1 suppression sensitizes KRAS mutant cancers to MEK inhibition by an IL17RD‐dependent mechanism. Sci Transl Med. 2019;11(483). 10.1126/scitranslmed.aaq1238 PMC6878763 30867319

[505] Meidhof S , Brabletz S , Lehmann W , et al. ZEB1‐associated drug resistance in cancer cells is reversed by the class I HDAC inhibitor mocetinostat. EMBO Mol Med. 2015;7(6):831‐847. 25872941 10.15252/emmm.201404396 PMC4459821

[506] Lee SH , Shen GN , Jung YS , et al. Antitumor effect of novel small chemical inhibitors of Snail‐p53 binding in K‐Ras‐mutated cancer cells. Oncogene. 2010;29(32):4576‐4587. 20531295 10.1038/onc.2010.208

[507] Tanaka K , Yu HA , Yang S , et al. Targeting Aurora B kinase prevents and overcomes resistance to EGFR inhibitors in lung cancer by enhancing BIM‐ and PUMA‐mediated apoptosis. Cancer Cell. 2021;39(9):1245‐1261. e6. 34388376 10.1016/j.ccell.2021.07.006 PMC8440494

[508] Feng YX , Sokol ES , Del Vecchio CA , et al. Epithelial‐to‐mesenchymal transition activates PERK‐eIF2alpha and sensitizes cells to endoplasmic reticulum stress. Cancer Discov. 2014;4(6):702‐715. 24705811 10.1158/2159-8290.CD-13-0945

[509] Ishay‐Ronen D , Diepenbruck M , Kalathur RKR , et al. Gain fat‐lose metastasis: converting invasive breast cancer cells into adipocytes inhibits cancer metastasis. Cancer Cell. 2019;35(1):17‐32. e6. 30645973 10.1016/j.ccell.2018.12.002

[510] Gupta PB , Onder TT , Jiang G , et al. Identification of selective inhibitors of cancer stem cells by high‐throughput screening. Cell. 2009;138(4):645‐659. 19682730 10.1016/j.cell.2009.06.034 PMC4892125

[511] Lei Z , Tan IB , Das K , et al. Identification of molecular subtypes of gastric cancer with different responses to PI3‐kinase inhibitors and 5‐fluorouracil. Gastroenterology. 2013;145(3):554‐565. 23684942 10.1053/j.gastro.2013.05.010

[512] She X , Gao Y , Zhao Y , Yin Y , Dong Z . A high‐throughput screen identifies inhibitors of lung cancer stem cells. Biomed Pharmacother. 2021;140:111748. 34044271 10.1016/j.biopha.2021.111748

[513] Lewis AC , Kats LM . Non‐genetic heterogeneity, altered cell fate and differentiation therapy. EMBO Mol Med. 2021;13(3):e12670. 33555144 10.15252/emmm.202012670 PMC7933953

[514] Stavropoulou V , Kaspar S , Brault L , et al. MLL‐AF9 expression in hematopoietic stem cells drives a highly invasive AML expressing EMT‐related genes linked to poor outcome. Cancer Cell. 2016;30(1):43‐58. 27344946 10.1016/j.ccell.2016.05.011

[515] Zanetti A , Affatato R , Centritto F , et al. All‐trans‐retinoic acid modulates the plasticity and inhibits the motility of breast cancer cells: role of notch1 and transforming growth factor (TGFbeta). J Biol Chem. 2015;290(29):17690‐17709. 26018078 10.1074/jbc.M115.638510 PMC4505019

[516] Shi G , Zheng X , Wu X , Wang S , Wang Y , Xing F . All‐trans retinoic acid reverses epithelial‐mesenchymal transition in paclitaxel‐resistant cells by inhibiting nuclear factor kappa B and upregulating gap junctions. Cancer Sci. 2019;110(1):379‐388. 30375704 10.1111/cas.13855 PMC6317959

[517] Latil M , Nassar D , Beck B , et al. Cell‐type‐specific chromatin states differentially prime squamous cell carcinoma tumor‐initiating cells for epithelial to mesenchymal transition. Cell Stem Cell. 2017;20(2):191‐204. e5. 27889319 10.1016/j.stem.2016.10.018 PMC5939571

[518] Pattabiraman DR , Bierie B , Kober KI , et al. Activation of PKA leads to mesenchymal‐to‐epithelial transition and loss of tumor‐initiating ability. Science. 2016;351(6277):aad3680. 26941323 10.1126/science.aad3680 PMC5131720

[519] Loo SY , Toh LP , Xie WH , et al. Fatty acid oxidation is a druggable gateway regulating cellular plasticity for driving metastasis in breast cancer. Sci Adv. 2021;7(41):eabh2443. 34613780 10.1126/sciadv.abh2443 PMC8494440

[520] Zhao N , Powell RT , Yuan X , et al. Morphological screening of mesenchymal mammary tumor organoids to identify drugs that reverse epithelial‐mesenchymal transition. Nat Commun. 2021;12(1):4262. 34253738 10.1038/s41467-021-24545-3 PMC8275587

[521] Mak MP , Tong P , Diao L , et al. A patient‐derived, pan‐cancer EMT signature identifies global molecular alterations and immune target enrichment following epithelial‐to‐mesenchymal transition. Clin Cancer Res. 2016;22(3):609‐620. 26420858 10.1158/1078-0432.CCR-15-0876 PMC4737991

[522] Cao R , Yuan L , Ma B , Wang G , Qiu W , Tian Y . An EMT‐related gene signature for the prognosis of human bladder cancer. J Cell Mol Med. 2020;24(1):605‐617. 31657881 10.1111/jcmm.14767 PMC6933372

[523] Simeonov KP , Byrns CN , Clark ML , et al. Single‐cell lineage tracing of metastatic cancer reveals selection of hybrid EMT states. Cancer Cell. 2021;39(8):1150‐1162. e9. 34115987 10.1016/j.ccell.2021.05.005 PMC8782207

[524] Bracken CP , Khew‐Goodall Y , Goodall GJ . Network‐based approaches to understand the roles of miR‐200 and other microRNAs in cancer. Cancer Res. 2015;75(13):2594‐2599. 26069247 10.1158/0008-5472.CAN-15-0287

[525] Kulkarni P , Dasgupta P , Hashimoto Y , et al. A lncRNA TCL6‐miR‐155 interaction regulates the Src‐Akt‐EMT network to mediate kidney cancer progression and metastasis. Cancer Res. 2021;81(6):1500‐1512. 33500248 10.1158/0008-5472.CAN-20-0832 PMC7969457

[526] Liu YN , Yin JJ , Abou‐Kheir W , et al. MiR‐1 and miR‐200 inhibit EMT via Slug‐dependent and tumorigenesis via Slug‐independent mechanisms. Oncogene. 2013;32(3):296‐306. 22370643 10.1038/onc.2012.58 PMC7580497

[527] Ru P , Steele R , Newhall P , Phillips NJ , Toth K , Ray RB . miRNA‐29b suppresses prostate cancer metastasis by regulating epithelial‐mesenchymal transition signaling. Mol Cancer Ther. 2012;11(5):1166‐1173. 22402125 10.1158/1535-7163.MCT-12-0100

[528] Zhang J , Zhang H , Liu J , et al. miR‐30 inhibits TGF‐beta1‐induced epithelial‐to‐mesenchymal transition in hepatocyte by targeting Snail1. Biochem Biophys Res Commun. 2012;417(3):1100‐1105. 22227196 10.1016/j.bbrc.2011.12.121

[529] Siemens H , Jackstadt R , Hunten S , et al. miR‐34 and SNAIL form a double‐negative feedback loop to regulate epithelial‐mesenchymal transitions. Cell Cycle. 2011;10(24):4256‐42571. 22134354 10.4161/cc.10.24.18552

[530] Zhou JN , Zeng Q , Wang HY , et al. MicroRNA‐125b attenuates epithelial‐mesenchymal transitions and targets stem‐like liver cancer cells through small mothers against decapentaplegic 2 and 4. Hepatology. 2015;62(3):801‐815. 25953743 10.1002/hep.27887

[531] Lin YH , Wu MH , Liao CJ , et al. Repression of microRNA‐130b by thyroid hormone enhances cell motility. J Hepatol. 2015;62(6):1328‐1340. 25617495 10.1016/j.jhep.2014.12.035

[532] Yu J , Lei R , Zhuang X , et al. MicroRNA‐182 targets SMAD7 to potentiate TGFbeta‐induced epithelial‐mesenchymal transition and metastasis of cancer cells. Nat Commun. 2016;7:13884. 27996004 10.1038/ncomms13884 PMC5187443

[533] Kim T , Veronese A , Pichiorri F , et al. p53 regulates epithelial‐mesenchymal transition through microRNAs targeting ZEB1 and ZEB2. J Exp Med. 2011;208(5):875‐883. 21518799 10.1084/jem.20110235 PMC3092351

[534] Moes M , Le Bechec A , Crespo I , et al. A novel network integrating a miRNA‐203/SNAI1 feedback loop which regulates epithelial to mesenchymal transition. PLoS One. 2012;7(4):e35440. 22514743 10.1371/journal.pone.0035440 PMC3325969

[535] Xia H , Ooi LL , Hui KM . MicroRNA‐216a/217‐induced epithelial‐mesenchymal transition targets PTEN and SMAD7 to promote drug resistance and recurrence of liver cancer. Hepatology. 2013;58(2):629‐641. 23471579 10.1002/hep.26369

[536] Chang RM , Yang H , Fang F , Xu JF , LY Yang . MicroRNA‐331‐3p promotes proliferation and metastasis of hepatocellular carcinoma by targeting PH domain and leucine‐rich repeat protein phosphatase. Hepatology. 2014;60(4):1251‐1263. 24825302 10.1002/hep.27221

[537] Qi J , Rice SJ , Salzberg AC , et al. MiR‐365 regulates lung cancer and developmental gene thyroid transcription factor 1. Cell Cycle. 2012;11(1):177‐186. 22185756 10.4161/cc.11.1.18576 PMC3272236

[538] Liu G , Yang D , Rupaimoole R , et al. Augmentation of response to chemotherapy by microRNA‐506 through regulation of RAD51 in serous ovarian cancers. J Natl Cancer Inst. 2015;107(7):djv108. 25995442 10.1093/jnci/djv108 PMC4554255

[539] Lu Y , Xiao L , Liu Y , et al. MIR517C inhibits autophagy and the epithelial‐to‐mesenchymal (‐like) transition phenotype in human glioblastoma through KPNA2‐dependent disruption of TP53 nuclear translocation. Autophagy. 2015;11(12):2213‐2232. 26553592 10.1080/15548627.2015.1108507 PMC4835194

[540] Diepenbruck M , Tiede S , Saxena M , et al. miR‐1199‐5p and Zeb1 function in a double‐negative feedback loop potentially coordinating EMT and tumour metastasis. Nat Commun. 2017;8(1):1168. 29079737 10.1038/s41467-017-01197-w PMC5660124

[541] Grelet S , Link LA , Howley B , et al. A regulated PNUTS mRNA to lncRNA splice switch mediates EMT and tumour progression. Nat Cell Biol. 2017;19(9):1105‐1115. 28825698 10.1038/ncb3595 PMC5578890

[542] Dhar S , Kumar A , Gomez CR , et al. MTA1‐activated Epi‐microRNA‐22 regulates E‐cadherin and prostate cancer invasiveness. FEBS Lett. 2017;591(6):924‐933. 28231399 10.1002/1873-3468.12603

[543] Li W , Zhang Z , Liu X , et al. The FOXN3‐NEAT1‐SIN3A repressor complex promotes progression of hormonally responsive breast cancer. J Clin Invest. 2017;127(9):3421‐3440. 28805661 10.1172/JCI94233 PMC5669564

[544] Meyer‐Schaller N , Cardner M , Diepenbruck M , et al. A hierarchical regulatory landscape during the multiple stages of EMT. Dev Cell. 2019;48(4):539‐553. e6. 30713070 10.1016/j.devcel.2018.12.023

[545] Capra J , Eskelinen S . Correlation between E‐cadherin interactions, survivin expression, and apoptosis in MDCK and ts‐Src MDCK cell culture models. Lab Invest. 2017;97(12):1453‐1470. 28892098 10.1038/labinvest.2017.89

[546] Han LL , Jia L , Wu F , Huang C . Sirtuin6 (SIRT6) promotes the EMT of hepatocellular carcinoma by stimulating autophagic degradation of E‐cadherin. Mol Cancer Res. 2019;17(11):2267‐2280. 31551254 10.1158/1541-7786.MCR-19-0321

[547] Liu H , Ma Y , He HW , Zhao WL , Shao RG . SPHK1 (sphingosine kinase 1) induces epithelial‐mesenchymal transition by promoting the autophagy‐linked lysosomal degradation of CDH1/E‐cadherin in hepatoma cells. Autophagy. 2017;13(5):900‐913. 28521610 10.1080/15548627.2017.1291479 PMC5446059

